# Radiocarbon dating and cultural dynamics across Mongolia’s early pastoral transition

**DOI:** 10.1371/journal.pone.0224241

**Published:** 2019-11-06

**Authors:** William Taylor, Shevan Wilkin, Joshua Wright, Michael Dee, Myagmar Erdene, Julia Clark, Tumurbaatar Tuvshinjargal, Jamsranjav Bayarsaikhan, William Fitzhugh, Nicole Boivin

**Affiliations:** 1 Max Planck Institute for the Science of Human History, Department of Archaeology, Jena, Germany; 2 University of Colorado Museum of Natural History, Boulder, United States of America; 3 University of Aberdeen, Department of Archaeology, Aberdeen, Scotland; 4 University of Groningen, Center for Isotope Research, Groningen, Netherlands; 5 National University of Mongolia, Department of Archaeology, Ulaanbaatar, Mongolia; 6 Flinders University, Adelaide, Australia; 7 Christian Albrechts University, Institute of Prehistoric and Protohistoric Archaeology, Keil, Germany; 8 National Museum of Mongolia, Ulaanbaatar, Mongolia; 9 Smithsonian Institute, Department of Archaeology, Arctic Studies Center, Washington D.C., United States of America; 10 University of Queensland, School of Social Science, Brisbane, Australia; 11 University of Calgary, Department of Anthropology and Archaeology, Calgary, Canada; 12 Smithsonian Institution, National Museum of Natural History, Washington D.C., United States of America; University at Buffalo - The State University of New York, UNITED STATES

## Abstract

The emergence of mobile herding lifeways in Mongolia and eastern Eurasia was one of the most crucial economic and cultural transitions in human prehistory. Understanding the process by which this played out, however, has been impeded by the absence of a precise chronological framework for the prehistoric era in Mongolia. One rare source of empirically dateable material useful for understanding eastern Eurasia’s pastoral tradition comes from the stone burial mounds and monumental constructions that began to appear across the landscape of Mongolia and adjacent regions during the Bronze Age (ca. 3000–700 BCE). Here, along with presenting 28 new radiocarbon dates from Mongolia’s earliest pastoral monumental burials, we synthesise, critically analyse, and model existing dates to present the first precision Bayesian radiocarbon model for the emergence and geographic spread of Bronze Age monument and burial forms. Model results demonstrate a cultural succession between ambiguously dated Afanasievo, Chemurchek, and Munkhkhairkhan traditions. Geographic patterning reveals the existence of important cultural frontiers during the second millennium BCE. This work demonstrates the utility of a Bayesian approach for investigating prehistoric cultural dynamics during the emergence of pastoral economies.

## Introduction

While archaeological data pertaining to Mongolia’s early pastoral lifeways are rare, a number of early mounded burial traditions provide an important exception. Recent paleoproteomic research (Jeong et al 2018) indicates that the people who produced these burials kept domestic livestock and made dairy products, and may represent the region’s initial pastoral occupants. By the late second millennium BCE, emerging research using ancient DNA [1,2] and proteins [3,4], stable isotope analysis [5], archaeozoology [6,7], and radiocarbon dating [8] –have helped to identify early pastoral management and key subsistence changes in Mongolia throughout the Bronze Age. These changes include the introduction of domestic livestock and their first use in milk production [3,4], as well as the adoption of reliable mounted horseback riding [8], processes that may have had important social and cultural impacts. However, the lack of chronological precision from this period–particularly the earliest archaeological features–deprives researchers of the most basic chrono-typological units necessary to explore how these processes unfolded. Here, we produce a comprehensive radiocarbon model and geographic visualization of 28 new AMS radiocarbon dates (on 26 individuals) along with a review of published data for a total of 200 AMS dates on human and animal bone from Mongolia’s early pastoral mounded burial and monument traditions, and explore their implications for understanding the emergence of pastoralism.

### Early evidence for pastoralism in Mongolia

**Fig 1:**
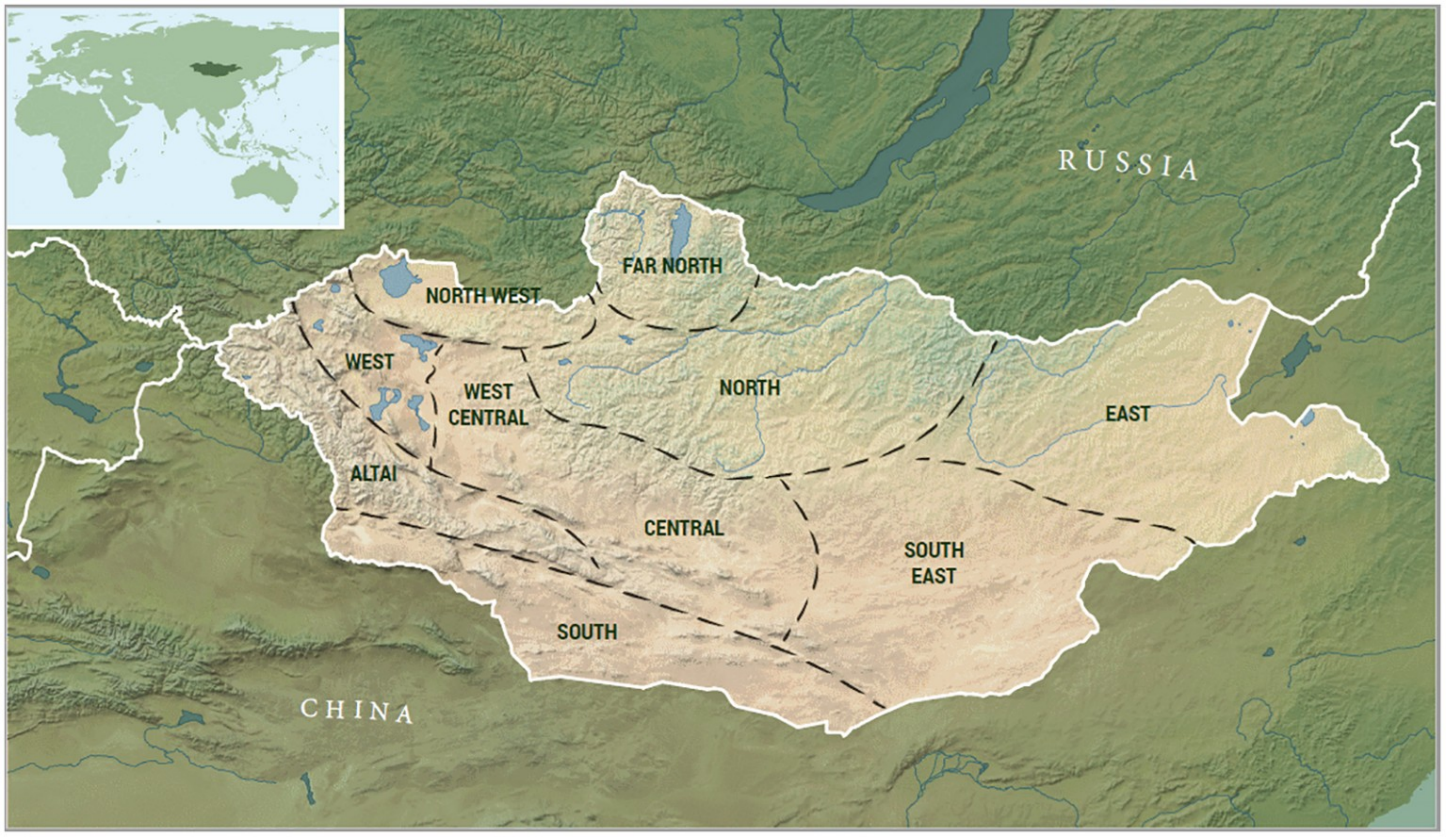
Study area and general regions referred to in the analysis.

While some influential scholarship [9,10] has linked the adoption of pastoralism in Mongolia (Fig 1) with the emergence of state-level, agricultural societies in China during the first millennium BCE, more prescient authors [11] and a large body of archaeological research over recent decades [3,6, 12, 13, 14; 15; 16] has established that herding economies emerged centuries earlier, with no apparent connection to agricultural zones of riverine East Asia. In fact, the earliest evidence for domestic animals in the eastern Eurasian steppe comes from burial features of the Afanasievo culture, dating to the late 4^th^ and early 3^rd^ millennia BCE. These features are characterized by stone burial mounds, often containing disassembled carts and the remains of domestic animals such as cattle, sheep/goat, and dogs [17,18]. In the nearby Altai region of Russia, genetic research on human remains from the Afanasievo culture show a close affinity with the Yamnaya of eastern Europe, pointing to a migration deep into the Eurasian interior during the mid to late 4^th^ millennium BCE [19, 20]. Similar Afanasievo-style archaeological features are also found within the territory of Mongolia [17], and preliminary mitochondrial DNA research supports their links with Yamnaya [20]. Recently, archaeological milk proteins discovered in dental calculus from these Afanasievo burials provide the first unequivocal evidence that these early people participated in a dairy-based, livestock economy [4] strengthening the plausibility of the idea that Afanasievo people were a vector for the initial introduction of domestic ruminants into the Mongolian steppe [21].

**Fig 2:**
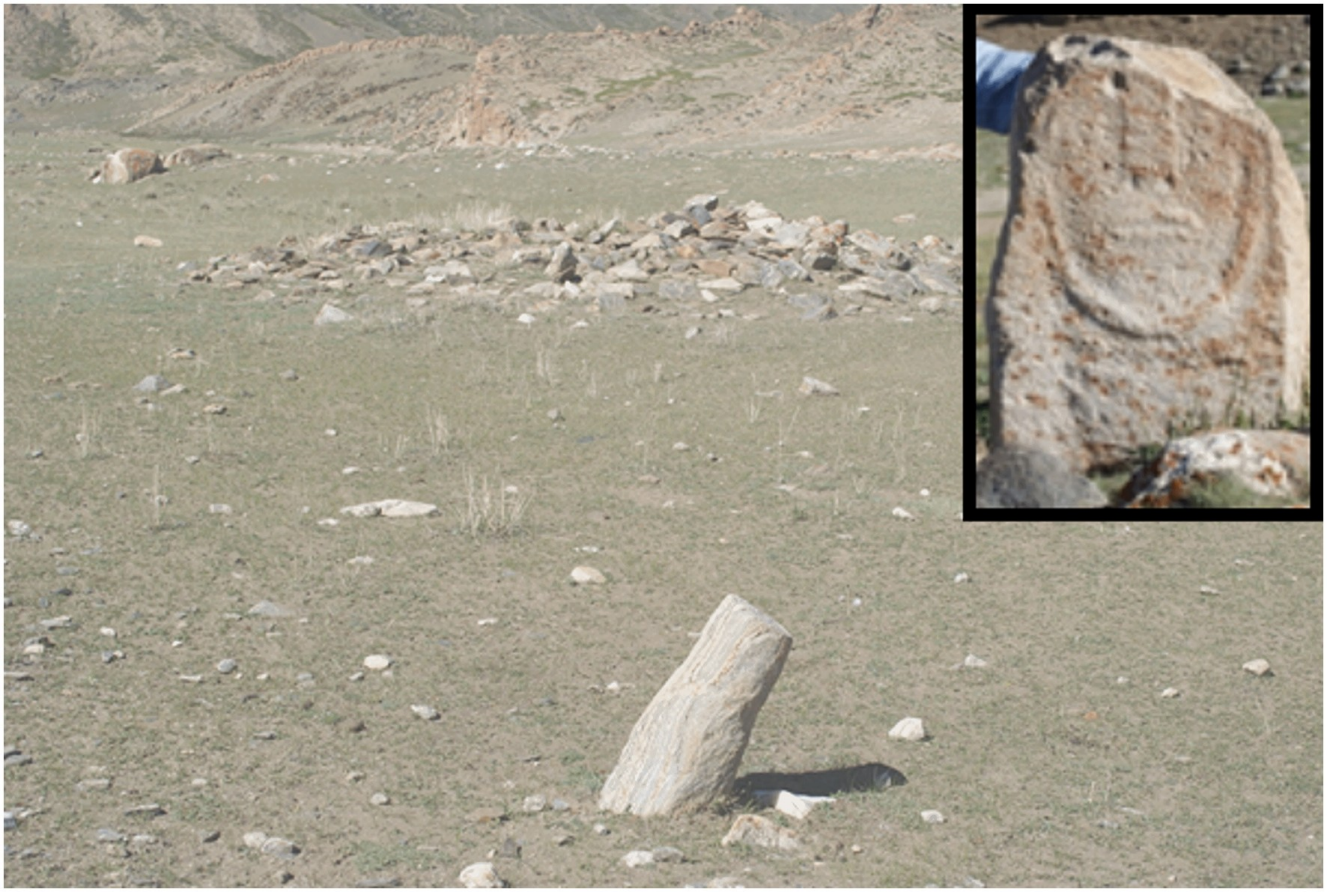
Chemurchek burial mound in western Mongolia, along with accompanying standing stone Fig. Photo: T. Tuvshinjargal.

Also accompanying the Afanasievo in the early Bronze Age is the Chemurchek burial tradition (sometimes referred to using the Chinese *Qiemuerqieke* or the Mongolian *Hemtseg*). The Chemurchek mortuary tradition is characterized by rectangular burials accompanied by standing stones, which often show an anthropomorphic Fig, sometimes holding a ‘shepherd’s crook’-like object (Fig 2). Dental calculus from Chemurchek burials in western Mongolia also clearly demonstrate the participation in a pastoral or partially pastoral subsistence system [3]. Sites affiliated with this tradition are also found in China and Russia [18,22].

Within Mongolia, there remains disagreement as to which burial traits distinguish Chemurchek from Afanasievo–as some early features show Afanasievo-like construction (flat mounds with curbed walls) but display a rectangular shape. Published radiocarbon dates from both Afanasievo and Chemurchek contexts suffer from incredibly broad error ranges, leading to ambiguity in interpreting the initiation and duration of associated cultural events [18, 22], and some doubt as to whether Chemurchek may precede, overlap with, or follow the Afanasievo tradition [22]. However, despite their poor chronological resolution, these traditions appear to represent the earliest groups linked with herding lifeways in the eastern Steppes.

By the end of the second millennium a wide range of archaeological data demonstrate a florescence of mobile herding lifeways, and suggest a major transition in the use of horses as both transport and livestock. At ca. 1200 BCE, horses become a ubiquitous feature of ritual and burial sites known as deer stones (carved standing stones) and *khirigsuurs* (stone mounds surrounded by a ground-level, square or circular stone ‘fence’), collectively referred to as the Deer Stone Khirigsuur Complex [23]. These monument types are often, though not universally, considered a single cultural horizon, along with the khirigsuur-like *Sagsai* monuments often found on hillslopes [6], creating some terminological difficulty in referencing this period.

A diverse body of data point to important changes in Mongolia’s pastoral economy at the end of the second millennium BCE, linked to horses, but leave a murkier picture of pastoralism before the horse-riding era. Horse remains from ritual features at both deer stones and *khirigsuurs* show that these animals were bridled [24], heavily exerted and likely ridden [7], managed as a breeding herd [25] and even cared for using new forms of veterinary dentistry [26]. Horse remains are also found in isolated features of other Bronze Age cultures [8]. This evidence aligns with the appearance of horse in the few known dietary assemblages dating to the late Bronze Age [6]. Most recently, milk proteins from human dental calculus link the end of the second millennium BCE with the first consumption of horse milk, which remains a significant component of subsistence in the region even today [4]. All told, the end of the second millennium BCE marked a major transition in mobility and the use of horses as livestock, likely assisted by the adoption of mounted horseback riding [27]. However, these changes appear to date to relatively late in the Bronze Age ca. 1200 BCE [8], several millennia after the first direct evidence of livestock-based economies [4, 21]. Understanding the development of pastoral economies in eastern Eurasia requires bridging these key Late Bronze Age phenomena with the cultural traditions of the 3rd and early 2nd millennia BCE.

**Fig 3:**
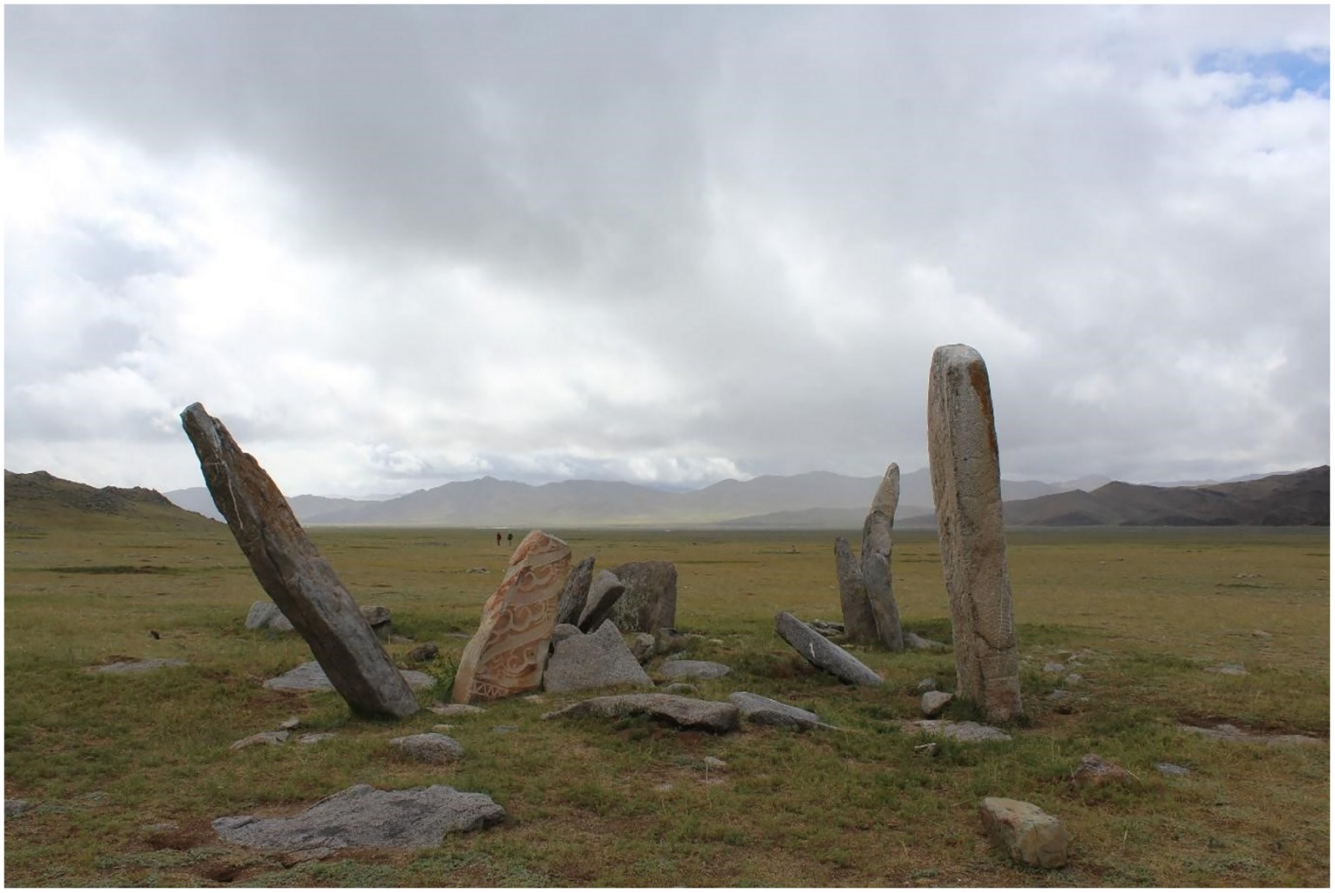
Slab burial built of uprooted deer stones at the site of Shatar Chuluu in Bayankhongor, central Mongolia.

The intricacy of the monumental record of the late Bronze Age complicates assessment of how this economic transition may have influenced early pastoral cultures. It is possible that any given valley or even a single cemetery might contain a variety of distinct burial and monument traditions—such as *khirigsuurs*, Sagsai mounds, rows of “Ulaanzuukh” square burials, large square burials built from stone slabs (“slab burials”), hourglass-shaped constructions (“shape burials”), and/or other prehistoric and early historic features—all occupying a narrow window in the late second and early first millennium BCE (see [28,29,30] for examples). Nonetheless, researchers have identified important archaeological patterns that may reflect cultural dynamics and help construct chronological sequences, such as slab burials built from uprooted deer stones (Fig 3) that postdate DSK constructions in areas of their occurrence [31,32].

### Radiocarbon modeling Mongolia’s early pastoral traditions

To use radiocarbon dates to make meaningful inferences regarding cultural dynamics in early Mongolia, a number of key challenges must first be addressed. First, and most problematically, is the relationship between monument construction and cultural activity. The territory of Mongolia appears to have been occupied by modern humans since at least the Upper Paleolithic [33]. However, due to a combination of taphonomic, geologic, and cultural factors, intact and dateable habitation sites from early prehistory in the region are nearly absent [34]. Burial mounds with visible above-surface features appear only in the late 4th and early 3rd millennium BCE [35]. Bronze Age monument types exhibit a wide range of morphological traits, leading to typological debate and ambiguity. Without accompanying habitation sites, it is also impossible to be sure whether mounded burials accurately reflect the spatial distribution of early pastoralists on the landscape. Nonetheless, these monumental constructions provide a useful proxy for cultural activity. Imperfect as they are, their construction co-occurs with the first direct evidence of livestock economies, and the construction of monumental features continues to be a defining trait of many pastoral cultures well into the historic era. These features also provide the only large, spatially expansive, and scientifically dateable archaeological dataset with which to explore early cultural dynamics at this time.

Another series of methodological concerns relate to the process of radiocarbon dating itself, and how to connect radiocarbon measurements with cultural phenomena of interest. In regions of Mongolia with expansive lakes (particularly the northern and western regions), AMS radiocarbon measurements taken on human bone could appear earlier than the true date of construction due to the “freshwater” reservoir effect, wherein the consumption of fish resources from waterways with carbonates leached from the surrounding geology biases the radiocarbon concentration, and hence the age, of human tissue [36]. Conversely, dates drawn from wood can be problematised by the “old wood” effect, wherein the measured age of the sample does not reflect the last growth years of the plant, but rather material laid down much earlier, which could be offset from the cultural event of interest by tens or even hundreds of years [37]. This challenge can be addressed when dates are obtained from wood samples that have been identified to species level, but in Mongolia such an approach has rarely been taken. Radiocarbon dates drawn from large herbivore tissue are generally considered to be reliable material for dating the animal’s death [38] but recent work by Zazzo et al. [39] shows that porous bone buried at shallow depths in Bronze Age Mongolian features can be contaminated by intrusive organic material, biasing radiocarbon dates toward younger ages and potentially influencing archaeological interpretations.

## Materials and methods

In order to address these concerns and produce a robust chronological framework for Mongolia’s early pastoral cultures useful for making inferences about the hunting/herding transition, we compiled and critically analysed published radiocarbon dates from mounded burial/monument constructions corresponding roughly to the Bronze and Early Iron Ages (ca. 3000–500 BCE). In partnership with the National University of Mongolia’s Department of Archaeology, we also produced 28 new radiocarbon dates from 26 individuals, testing human bone and teeth from burials spanning this period in the University’s archaeological repository (see Acknowledgments for details).

Sampling was conducted at the National University of Mongolia on human tooth and bone. For samples of bone, pieces weighing between 0.5 and 2 g were collected and labeled in individual plastic bags. When available, whole teeth were sent intact for analysis, and when teeth had preexisting fractures, dentine samples of ~200 mg were drilled with a Dremel 3000 tool and collected into Eppendorf tubes within a clean laboratory environment. When drilling was necessary, all associated laboratory surfaces and Dremel parts were cleaned with methanol between each sample. Analyzed samples included four specimens dated at the Center for Stable Isotope Research at the University of Groningen and 20 at the Oxford Radiocarbon Accelerator Unit (S1 Appendix, S1 Table).

To combat ambiguity in the definition of cultural categories, we produced a trait-based typological classification that uses specific features of construction of burial and monument features to create cultural units (Table 1). We deliberately selected those construction features that can be most widely assessed using published data (body position, structure shape, edge construction, mound height, burial chamber type, and presence or absence of ‘fence’ features). We excluded other traits, such as cardinal direction orientation or the inclusion of burial goods, which may be important but were sometimes difficult to trace in the available literature, or difficult to assess due to post-interment disruption of graves. In cases where published dates did not reliably fit these criteria, we excluded them from our model. In the case of “satellite” features that come from a closed context but are of uncertain chronological association with the associated monument [39], we grouped these features as a separate phase. All dates included in our model are summarized in S1 Appendix.

**Table 1:** Burial traits used in classification scheme. For detailed description of architectural traits see Eregzen 2016, Wright 2007, Wright 2014, Kovalev and Erdenebaatar 2009, Fitzhugh 2009.

A	B	C	D	E	F	G	H
Body position	Stone structure shape	Edge construction	Mound height	Burial chamber type	Ground -level features	Burial tradition designation	Other terms in use
Flexed	Circular	Curbed	Flat	Pit	No fence	Afanasievo	
Flexed	Rectangular	Curbed	Flat	Pit	No fence	Afanasievo, Chemurchek	Hemtseg
Flexed	Rectangular		Mounded	Slab cist	No fence	Chemurchek	Hemtseg, Qiemuerqieke, Shamirshak
Flexed	Rectangular OR Circular		Flat	Pit	No fence	Munkhkhairkhan	
Prone	Rectangular	Leaned slabs, layered stone	Flat	Pit	No fence	Ulaanzuukh	
Prone	Waisted/hourglass	Layered stone	Flat	Pit	No fence	Shape Burial	Helbert Bulsh, Shorgooljin Bulsh, Ant-Shape Grave, Tevsh
Prone	Half-circle	Layered stone	Flat	Pit	No fence	D-Shape	Tevsh, Stirrup
Supine	Circular		Mounded		Fence	Khirigsuur	Kereksur, Deer Stone-Khirigsuur Complex
Supine	Rectangular OR Circular	Four corner posts		Pit	No fence	Sagsai	Slope burials, Munguntaiga
Supine with knees up	Hollow circle		Flat	Pit	No fence	Baitag	
Supine	Rectangular	Upright slabs	Flat	Pit	No fence	Slab Burial	Slab Grave, Duruvljin Bulsh
						Unassigned stone feature	

For new radiocarbon dates, samples were decalcified over at least a 24-hour period using mild acid (HCl, 2–4% w/vol; RT) at the Center for Stable Isotope Research at the University of Groningen. For each sample still not fully decalcified, we refreshed the solution, removing and storing soft portions separately in demineralised water until further preparation. Soft and pliable fragments were rinsed thoroughly with demineralised water. Extracts were then exposed to NaOH (1%, ~30 min) to eliminate humic acids, rinsed to neutrality and treated once more with acid (HCl, 4% w/vol, 15 min). The raw collagen fraction was denatured to gelatin in acidified demineralised water (pH 3) at 80 °C for 18 hours. Before drying, the dissolved gelatin was filtered through a 50 μm mesh to eliminate any remaining foreign particulates, and the crystalline collagen scraped from the glass. Approximately 4 mg aliquots of the reduced carbon fraction were then weighed into tin capsules for combustion in an Elemental Analyser (EA, IsotopeCube NCS, Elementar^®^). The EA was coupled to an Isotope Ratio Mass Spectrometer (IRMS, Isoprime^®^ 100), allowing the d^13^C value of the sample to be measured, as well as a fully automated cryogenic system to trap CO_2_ liberated on combustion. After run completion, the individual reaction vessels were transferred to a graphitisation manifold, where a stoichiometric excess of H_2_ gas (1: 2.5) was added, and the CO_2_ gas reduced to graphite over an Fe(s) catalyst. The graphite samples were then pressed, and the radioisotopic ratio determined on a MICADAS accelerator mass spectrometer.

The ORAU followed routine pretreatment and measurement procedures [40]. For each specimen, between 200–600mg of bone or dentine was drilled using a handheld dentist drill, and collagen was extracted through as series of chemical steps that involved immersion in HCl, removal of humic acids using NaOH and removal of adsorbed CO_2_ via a final HCl wash. Only 4 of the samples prepared at the ORAU (OxA-36230, -36231, -36232, -36233; indicated with an asterisk) underwent ultrafiltration using Vivaspin ultrafilters, due to initial indications of poor collagen perseveration. Extracted collagen was frozen overnight and was lyophilized. Between 2–5 mg of collagen was combusted in an elemental analyser (EA) and its C and N stable isotopes were measured at an IRMS instrument linked to the EA, before excess gas CO_2_ was collected, graphitized and measured at an HVEE accelerator, alongside blanks and standards. These were used for contamination calculation and final correction of the data. Collagen yields ranged greatly from 0.8% to 17.8%, and the C:N ratio of the extracted collagen fell within expected ranges (3.2–3.4) with the exception of OxA-36233 (C:N = 3.6) and %C in the combusted collagen was between 37–46%.

To combat contamination and analytical bias in our data set, we included only direct dates on human and domestic animal bone from primary, closed archaeological contexts (e.g. within a burial chamber) in our models. In practice, this meant excluding dates that came from upper stratigraphic layers which might be considered intrusive, as well as wood material from early Afanasievo burials, and charcoal dates from later Bronze Age features (eliminating any concern over the old wood effect). Our resulting sample is still at risk of bias towards younger dates in porous bone from shallow burials, as well as bias towards older dates as a result of a potential dietary freshwater effect among some human samples. To assess the potential impact of poor quality control and radiocarbon contamination, for those phases with suitable dates, we also produced a QC restricted model using only dates that pass criteria recommended by Zazzo et al. [39] i.e. collagen yields greater than 5% and C:N ratio lower than 3.30. For those phases with dates from both human and herbivore bone (*khirigsuur* and slab burials), we also produced separate phases for each material type to compare offsets.

To test the relative ordering of existing typological categories and assess underlying cultural processes, we modeled burials according to both trait-based types (Table 1, column G) and body position within the burials (Table 1, column A). For each burial tradition or monument category, we produced a Bayesian uniform phase model in the program OxCal, in order to estimate the onset, duration, end date, and relative ordering of these monument and mounded burial traditions in Mongolia. Although in our first iteration of the burial tradition model we included all published dates from the site of Tsatsyn Ereg, a large deer stone and *khirigsuur* site in central Mongolia that has been extensively radiocarbon dated [39], the inclusion of nearly 100 dates from a single locality and restricted time interval caused our models to be dominated by this locality, and such models failed standard measures of agreement(A = 15). In our final model, we included two dates on horse remains from *khirigsuur* KTS01, three dates on horse remains from deer stone 38, and ten dates on animal remains sampled from different structural components of *khirigsuur* B10– including Feature 5 (3 dates), Feature 11 (2 dates), Feature 107, Feature 111, Feature 118, and Feature 666.

For features with multiple published or newly produced dates on human or animal bone from the same skeleton, we combined all published dates using the R_Combine function and employing an outlier model distributed according to a Student’s t-distribution, with 5 degrees of freedom [as outlined in [41]. Three pairs of dates taken from single specimens failed the chi-square test for goodness of fit, and their inclusion significantly impacted model agreement. These specimens originated from either the ambiguous Chemurchek/Afanasievo features (Khuurai Gobi Kurgan 2, Khundii Gobi Kurgan 1) or Munkhkhairkhan features (Ulaan Goviin Uzuur 2), wherein the previous measured date was several centuries younger than our newly produced date–presumably due to contamination. In our final model, we excluded the older date in favor of our new measurement, although all dates are summarized in Supplementary Material, and rerunning models with their inclusion did not meaningfully alter model outcomes.

In order to evaluate whether our models were influenced by outliers, we repeated these analyses using a trapezoidal prior probability distribution, and using a simple outlier model. As these outlier models did not provide significantly different results, beyond the exclusion criteria described above, no samples were removed from our final model. For each modeled parameter of interest (e.g. start and end dates for each group), we performed further tests of their relative ordering using the ‘Order’ function [42]. Finally, in order to explore possible connections transecting burial traditions, we repeated our analyses (uniform, trapezoid, and outlier models) with our dates grouped according to burial position, which has been identified as a comparatively stable marker of cultural or biological connections [43]. For these ‘burial position’ analyses, only direct dates on human bones were included in the model, and all animal bones and satellite features were excluded. All OxCal code used in analyses is provided in S1 Appendix.

## Results

**Fig 4:**
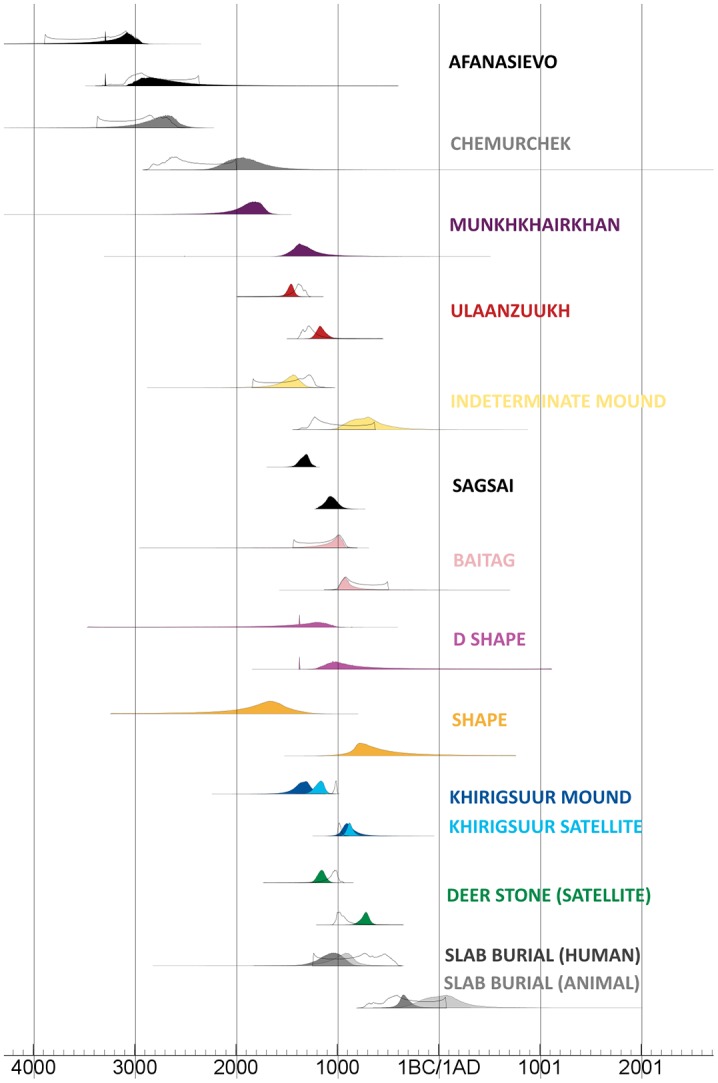
Modeled start boundaries for Bronze and Early Iron Age traditions in Mongolia, showing relative ordering according to posterior results. For each tradition with available data, the quality control-restricted model estimates are provided as an uncolored distribution outlined in black.

Of our 28 new dates, all but four produced successful AMS measurements (including two specimens that failed at Oxford but produced successful results at Groningen). However, only eight of these 24 dates produced both a collagen yield of 5% or greater and a C:N ratio of less than 3.30, as recommended by Zazzo et al. [39]. All models ran successfully to completion. Repeating our analysis using an outlier model did not significantly alter or improve our results. Of the three models, the trapezoidal prior provided the best model agreement when dates were grouped according to burial traditions (A = 64) and burial position (A = 91.3). Although all three approaches provided good agreement for the burial position model (A = 89.2 for uniform, A = 66.7 for outlier model), when broken up according to burial traditions the uniform prior and outlier model provided results below the arbitrary agreement threshold of A = 60. Our resulting trapezoidal prior models, used in subsequent analyses, provides a high-precision chronology for the onset and termination of key cultural events in Mongolian prehistory with important implications for the origins of pastoralism in the region. Our results from relative cultural chronology and ordering are summarized in S1 Appendix, S2 Table and Fig 4.

### Burial tradition chronology model

Our model results reveal a chronological sequence that provides broad agreement with previous models [17,35, 44] augmenting these with greater precision while also revealing important clarifications regarding the relative ordering of early pastoral traditions. Our new radiocarbon dating reveals that the oldest known monumental burial features in the territory of Mongolia are Afanasievo type burial mounds in the Southwestern Khangai Mountains of central Mongolia (Bayankhongor province, Shatar Chuluu), newly dated to 4410 +/-31 and 4415 +/- 31 radiocarbon years BP (S1 Appendix, S1 Table), with a modeled start boundary of 3341–2954 cal BCE (1 sigma range). Chronologically, the modeled end boundary for the Afanasievo culture in Mongolia overlaps with the onset of the Chemurchek tradition which began ca. 2923–2576 cal. BCE (1 sigma cal. range), and terminated by ca. 2136–1755 cal. BCE. Following Chemurchek, the initial second millennium BCE is represented by a handful of features from the Munkkhairkhan (1959–1724 cal. BCE, 1 sigma) culture. Table 2 uses Oxcal’s ‘Order’ function to examine the likelihood of overlap and definition between two sets of dates. Despite the small sample of available dates, and assuming that these patterns are not influenced by dietary freshwater reservoir effects, our analysis (Table 2, Fig 4) suggests a high probability that Afanasievo culture predates the onset of Chemurchek feature construction (92.2%), and that the onset of Chemurchek features preceded that of Munkkhairkhan (99.5%).

**Table 2:** Results of Oxcal “Order” function test for culture start (3A, top) and end (3B, bottom) boundaries, showing probability that t_1_ (y axis) start and end boundary event preceded t_2_ (x axis) start and end boundary event, based on posterior model results. Results are shaded in red according to significance, with the deepest red indicating lowest probability.

***Probability t***_**1 >**_***t***_**2**_	***t***_**2**_	Afanasievo Start	Chemurchek Start	Afanasievo End	Chemurchek End	Munkkhairkhan Start	Shape Burial Start	Ulaanzuukh Start	D Shape Start	Khirigsuur M Start	Munkhkhairkhan End	Sagsai Start	Khirigsuur S Start	DS S Start	Ulaanzuukh End	Sagsai End	Baitag Start	Slab Burial Start	Slab Burial Animal Start	Baitag End	Khirigsuur M End	Khirigsuur S End	D Shape End	DS S End	Shape Burial End	Slab Burial End	Slab Burial Animal End
***t***_**1**_																											
**Afanasievo Start**			0.9221	0.9858	1	0.9989	0.994	1	0.9663	1	1	1	1	1	1	1	1	1	1	1	1	1	1	1	1	1	1
**Chemurchek Start**		0.07786		0.6042	1	0.9956	0.9666	1	0.9169	1	1	1	1	1	1	1	0.9959	1	0.9999	1	1	1	1	1	1	1	1
**Afanasievo End**		0.014185	0.3958		0.9114	0.9088	0.8868	0.9527	0.8573	0.959	0.9613	0.961	0.9685	0.9695	0.9694	0.9736	0.9642	0.9731	0.9768	0.9828	0.9816	0.9815	0.9847	0.9871	0.9911	0.9999	1
**Chemurchek End**		0	0	0.08857		0.5556	0.6396	0.9463	0.7321	0.9611	0.9679	0.9704	0.9835	0.9848	0.9846	0.9891	0.9532	0.9879	0.987	0.9946	0.9943	0.9944	0.9942	0.9964	0.997	0.9989	0.9996
**Munkkhairkhan Start**		0.00113	0.004398	0.09118	0.4444		0.6617	1	0.7498	0.9959	1	1	1	1	1	1	0.9668	1	0.9966	1	1	1	1	1	1	1	1
**Shape Burial Start**		0.005991	0.03336	0.11315	0.3604	0.3383		0.9076	0.7021	0.9537	0.9653	0.9782	0.9978	0.9986	0.9986	0.9996	0.953	0.9979	0.9918	1	1	1	0.9997	1	1	1	1
**Ulaanzuukh Start**		0	0	0.04735	0.05374	0	0.0924		0.553	0.8767	0.8927	0.9865	1	1	1	1	0.9165	0.9975	0.981	1	1	1	0.9994	1	1	1	1
**D Shape Start**		0.03371	0.08312	0.14273	0.26787	0.25022	0.29794	0.447		0.5645	0.6078	0.5883	0.8052	0.8384	0.8353	0.9428	0.8578	0.9104	0.9431	0.9983	0.998	0.9989	0.9764	0.9997	0.9988	1	1
**Khirigsuur M Start**		0	0	0.04101	0.03894	0.00415	0.04629	0.12327	0.4355		0.5393	0.5842	0.9678	0.9842	0.9821	0.9984	0.8724	0.9692	0.96	1	1	1	0.9965	1	0.9999	1	1
**Munkkhairkhan End**		0	0	0.03867	0.03215	0	0.03467	0.1073	0.3922	0.4607		0.5425	0.8553	0.8793	0.8763	0.9337	0.8188	0.9056	0.921	0.9783	0.9772	0.9789	0.9669	0.9892	0.9905	0.9975	0.9991
**Sagsai Start**		0	0	0.03902	0.029581	0	0.021794	0.013506	0.4117	0.4158	0.4575		0.9824	0.9948	0.9939	0.9998	0.8654	0.9692	0.9574	1	1	1	0.997	1	0.9999	1	1
**Khirigsuur S Start**		0	0	0.031527	0.016482	0	0.002156	0	0.19476	0.0322	0.14466	0.017575		0.6272	0.6014	0.9363	0.7474	0.8146	0.8931	0.9998	0.9999	1	0.9653	1	0.9987	1	1
**DS S Start**		0	0	0.030489	0.015208	0	0.001371	0	0.16161	0.015797	0.12066	0.005227	0.3728		0.4799	0.8946	0.7203	0.7717	0.8762	0.9998	0.9998	1	0.9507	1	0.9982	1	1
**Ulaanzuukh End**		0	0	0.030599	0.015373	0	0.0014	0	0.16467	0.017893	0.12374	0.006086	0.3986	0.5201		0.8791	0.7149	0.7677	0.8734	0.9992	0.9989	0.9998	0.9469	1	0.998	1	1
**Sagsai End**		0	0	0.026373	0.010857	0	0.000368	0	0.05722	0.001633	0.06633	0	0.06374	0.1054	0.12088		0.5065	0.5042	0.7505	0.9803	0.9767	0.9937	0.8224	0.9999	0.991	1	1
**Baitag Start**		0	0.004122	0.03583	0.04678	0.03318	0.04701	0.08347	0.14219	0.12765	0.18116	0.13464	0.25262	0.27974	0.28513	0.4935		0.5272	0.7499	0.9713	0.9666	0.9971	0.8178	1	0.9897	1	1
**Slab Burial Start**		0	0	0.026851	0.01212	0	0.002069	0.002462	0.08955	0.030819	0.09442	0.030845	0.18536	0.2283	0.23229	0.4958	0.4728		0.7198	0.9312	0.9328	0.9675	0.8077	0.9997	0.9868	1	1
**Slab Burial animal Start**		0	0	0.023238	0.01303	0.003366	0.008216	0.019025	0.05692	0.03998	0.07902	0.04257	0.10693	0.12375	0.12665	0.24954	0.25015	0.28024		0.7107	0.7109	0.7533	0.6671	0.9679	0.945	1	1
**Baitag End**		0	0	0.017174	0.005442	0	0	0	0.00172	0	0.021689	0	0	0	0	0.019704	0.028679	0.06882	0.28928		0.504	0.579	0.5135	0.8364	0.8409	0.955	0.9786
**Khirigsuur M End**		0	0	0.018354	0.00569	0	0	0	0.002043	0	0.02279	0	0	0	0.001056	0.023303	0.03337	0.06718	0.28908	0.496		0.5598	0.5315	0.9227	0.8971	0.9977	0.9998
**Khirigsuur S End**		0	0	0.018477	0.00559	0	0	0	0.001069	0	0.021102	0	0	0	0	0.006284	0.002904	0.03249	0.24667	0.421	0.4402		0.5214	0.9921	0.928	1	1
**D Shape End**		0	0	0.015338	0.005843	0	0	0	0.023568	0.003515	0.03306	0.003014	0.03474	0.04929	0.05313	0.17764	0.18219	0.19234	0.3329	0.4865	0.4685	0.4786		0.6061	0.6597	0.7744	0.8514
**DS S End**		0	0	0.012855	0.003558	0	0	0	0	0	0.010779	0	0	0	0	0	0	0	0.03206	0.16359	0.07734	0.007876	0.3939		0.663	1	1
**Shape Burial End**		0	0	0.008903	0.002953	0	0	0	0.001158	0	0.009525	0	0.001265	0.001776	0.002032	0.009049	0.010281	0.013182	0.05502	0.15912	0.10293	0.07197	0.3403	0.337		0.8086	0.9181
**Slab Burial End**		0	0	0	0.001101	0	0	0	0	0	0.002522	0	0	0	0	0	0	0	0	0.04498	0.002252	0	0.22562	0	0.19139		0.9783
**Slab Burial animal End**		0	0	0	0	0	0	0	0	0	0	0	0	0	0	0	0	0	0	0.021441	0	0	0.14857	0	0.08186	0.021671	

Model start dates for shape burials, which are represented by a comparatively small sample (n = 5), span a wide range across the early second millennium BCE (ca. 1989–1453 cal. BCE, 1 sigma range) but interestingly appear to predate the onset of other LBA forms, including the Ulaanzuukh (ca. 1501–1434 cal. BCE), D Shape (1732–1031 cal. BCE, 1 sigma range) and Khirigsuur (1430–1265 cal. BCE, 1 sigma range). D-Shape and Baitag type, which are only known from a small corner of southern Mongolia, seem to date towards the turn of the first millennium BCE (Modeled Baitag start boundary dating to 1145–940 cal. BCE, 1 sigma range). The D-Shape burial tradition offers such a small sample size (n = 2) that its chronological position in not as clear as the others. The ‘Order’ function (Table 2) demonstrates that these regional forms are not easily differentiable in model start or end dates, though they are bracketed on either end by shape burials. Also falling within modeled start and end boundaries for shape burials are slab burials as well as Khirigsuur mound interments and Sagsai type graves, which are often linked with Khirigsuurs.

Chronologically, the only burial forms to persist beyond the 2nd millennium BCE are Shape Burials and Slab Burials. Slab Burials postdate both Khirisguurs (Order function probability 97%) and Sagsai type graves (97%). Slab Burials persist until the end of the 1st millenium BC (341 BCE median end boundary date). Importantly, those LBA features which produced dates on both human and animal bone (Khirigsuurs and Slab Burials) demonstrate a discrepancy of several centuries between these two groups (Fig 4), although Zazzo et al [39] have demonstrated that published radiocarbon ages from many horse remains from deer stones and *khirigsuurs* are likely impacted by contamination from shallow burials. In summary, while issues of quality control are difficult to resolve conclusively, our results reveal a likely chronological sequence of the first Bronze Age traditions (Afanasievo—Chemurchek—Munkkhairkhan), and a high degree of overlap between late Bronze Age traditions that can be further distinguished using burial position and spatial distribution.

### Burial positions

Burial positions provide a clearer picture of chronological difference and monumental forms and hint at important links and separations between Bronze Age monument traditions (Table 3). Initial Bronze Age features (Afanasievo, Chemurchek, Munkkhairkhan) are united by a common flexed burial position. In contrast, two alternative burial traditions emerge in the mid-2nd millennium BCE and people are mainly interred in either prone or supine positions. Beginning ca. 1567–1482 cal. BCE (1 sigma range), prone body position burials are the first of these new practices to emerge. Thereafter, ca. 1418–1343 cal. BCE (1 sigma range), supine burials also appear. These two burial positions are associated (for the most part) with particular burial forms: prone with Ulaanzuukh, Shape, and D-Shaped, and supine with Khirigsuur, Sagsai, and Slab Burials. Flexed burial types, while decreasing in overall spatial distribution, nonetheless persisted in the Altai until the turn of the millennium (ca. 1445–1128 cal. BCE, 1 sigma).

**Table 3:** Results of Oxcal “Order” function test for burial position start and end boundaries, showing probability that t_1_ (y axis) start and end boundary event preceded t_2_ (x axis) start and end boundary event, based on posterior model results. Results are shaded in red according to significance, with the deepest red indicating lowest probability.

***Probability t***_**1 >**_***t***_**2**_	***t***_**2**_	Flexed Start	Prone Start	Supine Start	Supine with knees bent Start	Flexed End	Prone End	Supine with knees bent End	Supine End
***t***_**1**_									
Flexed Start			1	1	0.991	1	1	1	1
Prone Start		0		0.995	0.841	0.895	1	1	1
Supine Start		0	0.005		0.634	0.715	1	1	1
Supine knees Start		0	0.158	0.365		0.587	0.989	1	1
Flexed End		0	0.105	0.3285	0.436		0.929	0.999	0.998
Prone End		0	0	0	0.011	0.071		0.999	0.999
Supine w/knees bent End		0	0	0	0	0.003	0.001		0.605
Supine End		0	0	0	0	0.003	0.001	0.395	

The inception of new prone and supine burial traditions, clearly associated with particular burial forms, is a key change in the mortuary record of Mongolia. Combined with monumental forms this suggests clear groupings of mortuary practice.

### Geographic visualizations

**Fig 5:**
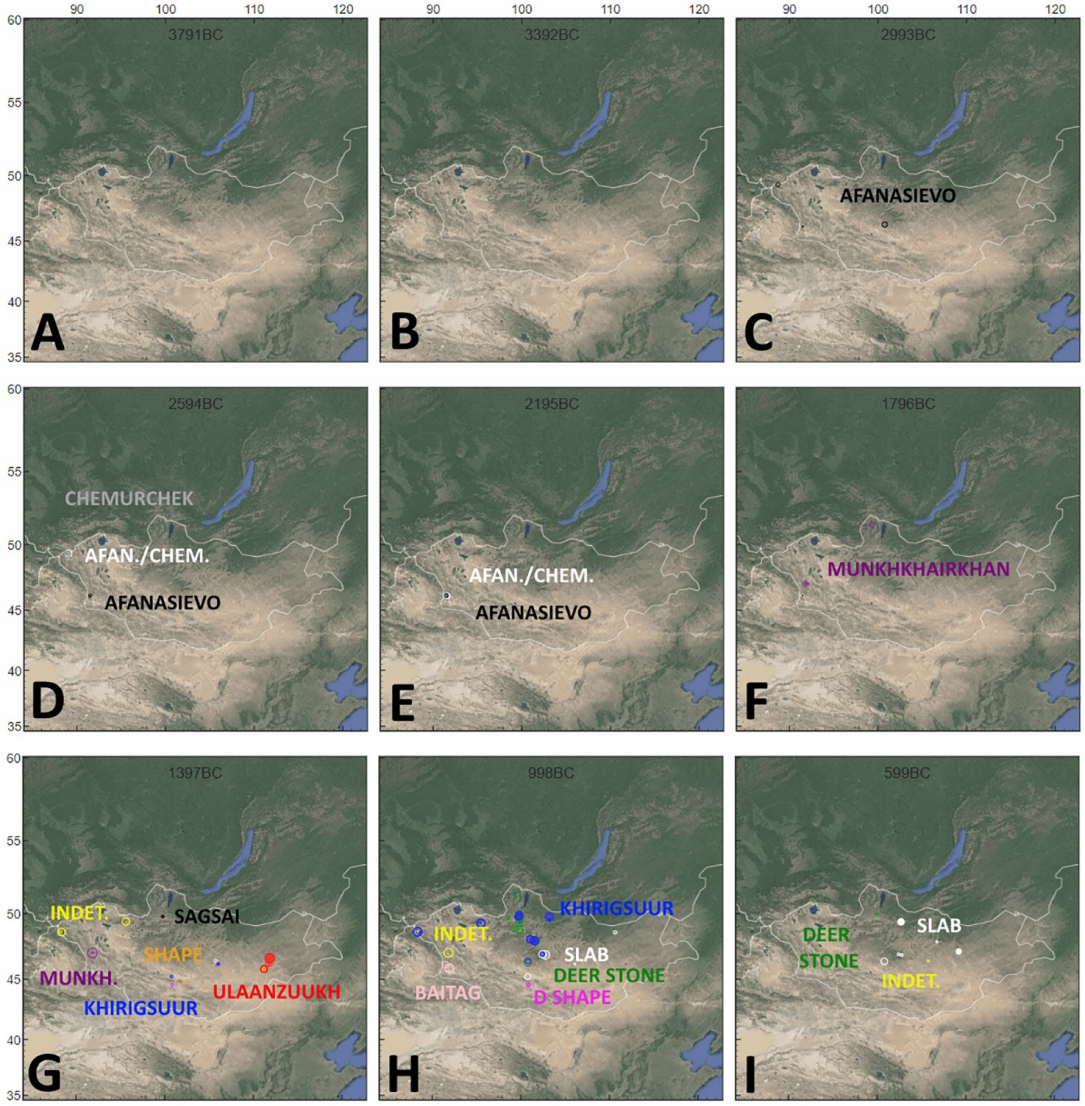
Posterior probability estimates for modeled dates according to cultural group. For each timeslice, the width of each circle corresponds to the portion of modeled posterior distribution that falls within the timeslice. Mounds with a published radiocarbon date, but not conforming to available cultural categories are marked in yellow as “Indet".

**Fig 6:**
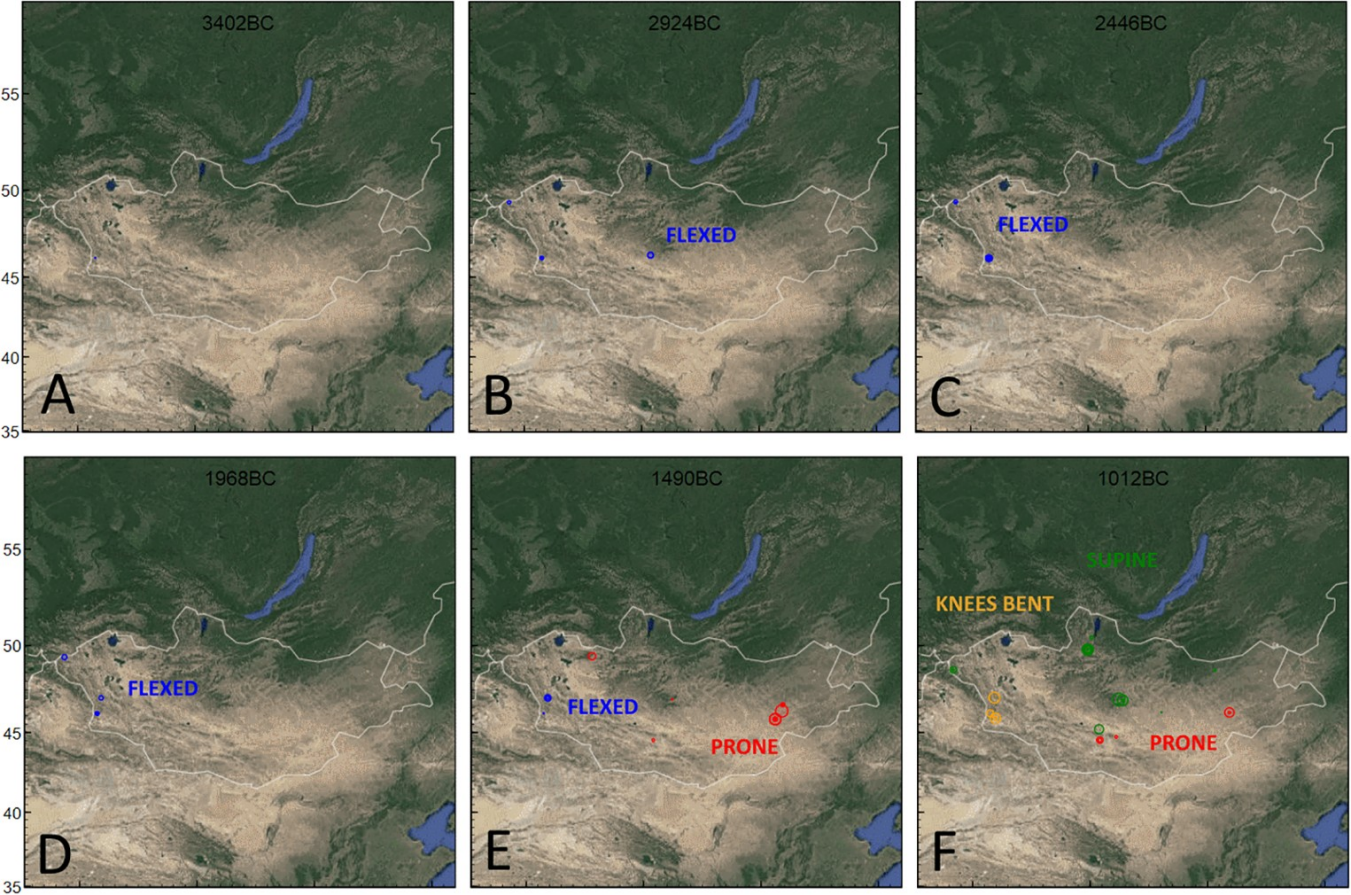
Posterior probability estimates for modeled dates according to burial position. For each box, the width of each circle corresponds to the portion of modeled posterior distribution that falls within the timeslice.

Some of the most interesting model results emerge when considered in terms of their geographical patterning using OxCal’s posterior probability mapping tool on dated burial monuments (Figs 5 and 6). Firstly, our results recapitulate findings [17,35] of a geographic concentration of Mongolia’s earliest monumental traditions (Afanasievo, Chemurchek and Munkhkhairkhan) to the western, southwestern and Altai regions of Mongolia (Fig 1A–1G). Combined with their shared flexed burial pattern (Fig 6A–6D) and overlapping cultural boundaries (Fig 5), these results suggest shared cultural traditions for the people who built Mongolia’s early Bronze Age burial monuments, as well as a likely entry of these traditions from western Eurasia.

Visualizations show that, beginning in the 16th century BCE, the earliest dated non-flexed burials appear in the archaeological record (Fig 6). Shape, Ulaanzuukh, and even untyped monument features in northern, central, west-central and southeastern regions of Mongolia (Fig 1) were united by a shared prone burial position, with an eventual concentration of these features in the central and southeastern parts of the country (Fig 5G). Interestingly, the earliest features from a number of supine burial groups (Khirigsuurs—Bor Ovoo in Bayankhongor province, southern part of central region, Slab Burials- Baga Gazriin Chuluu in Dundgovi province, southeastern region) occur in the same region as the earliest prone burials, a century or more later. Modeled probability distributions shown in Fig 4 and map visualizations shown in Fig 5 also show overlap in the spatial and chronological distribution of Sagsai Burials (often referred to as ‘slope burials’) and *khirigsuur*s, which could support previous inferences of a cultural link [6, 45]. Map patterns also exhibit a rough spatial segregation of the various other overlapping LBA monument traditions (Ulaanzuukh and Shape to the south), which points to the existence of a prone/supine cultural frontier (Figs 5G, 5H and 6F). During the early first millennium BCE, visualizations demonstrate the proliferation of supine burial types across the central and north regions (Fig 1) while prone burial tradition continues in the central and southeast regions—with dated Slab Burials outlasting all other traditions. (Fig 5I).

Notable across both visualisations is the proliferation of mortuary traditions and burial types between ca. 1200–1100 BCE. In the mid-2nd millennium BCE, there are only three recognized mortuary traditions, while just a few centuries later this Fig has more than doubled. In the same period, flexed burial positions apparently disappear, while several new traditions in burial position emerge. These findings speak to potential socio-cultural processes, perhaps groups differentiating themselves across an increasingly more densely occupied landscape. The emergence of horse-riding c.1200 BCE may also have brought groups in closer contact with one another, and increased demands for grazing land. The use of burial features as territorial markers, expressing ownership over or appropriation of resources—in this case land for grazing increasingly large and diverse herds—has been suggested in diverse cultural contexts globally [46,47].

## Discussion

### Diagenesis and quality control

Examination of quality control criteria indicates that only a portion (8 of 24) of newly acquired dates, and no published dates beyond those for deer stones and *khirigsuurs* at Tsatsyn Ereg, meet the benchmarks for ensuring the absence of contamination from Mongolian burial features advocated by Zazzo et al. [39] Therefore, modeled start and end boundaries are at risk of producing estimates that may be younger than the true phenomenon of interest, particularly those features that come from shallow contexts such as deer stone/*khirigsuur* horse remains. Our QC-restricted model, shown in Fig 4 (with modeled boundaries as white outlines) shows that after excluding dates of unknown collagen yield and C/N ratio, model estimates are greatly influenced by small sample sizes–producing either extremely wide error ranges (e.g. Afanasievo, Chemurchek), or narrow error ranges that may reflect the age of a single site rather than a broad tradition (e.g. *khirigsuurs* and Tsatsyn Ereg). Thus, while the anticipated effect of contamination is a rightward-shift (towards younger estimates) in both start and end boundaries, the observed impact of QC restriction in the available dataset is an ‘inward’ shift towards the few available datapoints. Future reporting of complete radiocarbon dataset will enable the creation of models that are robust to contamination issues and less impacted by sample size. In the current model, all inferences made must be considered at risk of bias in the younger direction from modern contamination (especially deer stone/*khirigsuur* animal features) and in the older direction from dietary reservoir impacts (for human bone).

### Implications

Model results reify the chronological primacy of the Afanasievo among the burial traditions linked with early pastoralists in eastern Eurasia, consistent with the idea that its spread into the eastern steppe may have introduced the region’s first domestic animals [21]. Despite the small available sample size of these early burials and the potential for diagenetic impacts on our modeled estimates, a test using the ‘Order function provides some statistical support (92.2% probability) that the Chemurchek tradition postdates the onset of Afanasievo cultural construction. While most early Afanasievo monuments are concentrated in the southwest, our new precision dates from Shatar Chuluu (Bayankhongor province) in the central region represent the oldest dated instances of Afanasievo culture in Mongolia (Fig 5). This new result suggests that the initial dispersal of the Afanasievo tradition reached into the southern slopes of the Khangai Mountains.

In addition to providing increased chronological precision for the burial traditions of these first pastoral inhabitants, our model indicates that the dated examples of the Chemurchek burial tradition came to an end by ca. 1917 cal. BCE (median date, 2136–1755 cal. BCE, 1 sigma). If model dates are not influenced by diagenetic processes, this timing would appear significant, as it suggests little overlap between Chemurchek and the earliest evidence for domestic horse (*E*. *caballus*) in Mongolia. Domestic horses definitely used for transport are found in Sintashta burials dated roughly ca. 2000 BCE in the steppes of adjoining areas of southern Russia and northern Kazakhstan [48]. However, domestic horse remains do not unequivocally appear in Mongolia until the mid-second millennium BCE, when have been found as occasional finds in ritual structures described as ‘Chemurchek’ [18]. Based on our model results, it seems unlikely that these relate to the culture that produced Chemurchek-style burial mounds.

The geographic patterning apparent in these first Bronze Age pastoral traditions corroborates inferences of clustering by early herders in mountains and mountain piedmonts, which, among other potential advantages, receive more reliable levels of orographic precipitation [27]. Palaeoenvironmental data suggest both a general pattern of drought across this period, as well as a shift towards a topography-dominated pattern of precipitation [49], which may have limited the regions viable for pastoral lifeways—particularly prior to the widespread adoption of mounted horseback riding. This western concentration in mountainous areas also appears to hold true for the subsequent Munkhkhairkhan culture (Fig 5), which is linked to Chemurchek and Afanasievo features through a common flexed burial position, and other structural traits such as curbed walls (Table 1). Even after the florescence of other monumental burial traditions in southeastern and central Mongolia–shape burials, Ulaanzuukh, Sagsai, Khirigsuur, and others–during the mid-second millennium BCE, flexed burial traditions persisted in the far west in the style of their regional predecessors.

Model results show supine and prone burial traditions anticipate or slightly predate the widespread appearance of horses in ritual burials, which raises the possibility that the shifting geographic distributions of these burial styles (Fig 6) was influenced by the introduction and adoption of horses into the pastoral economy. Although supine burials with bent knees (Fig 6F) have received little attention in the Mongolian cultural record, similar body positions are reported from undated Bronze Age features in Xinjiang [22] and southeastern Mongolia [50] and it is likely that their occurrence in Mongolia was part of a broader tradition extending across the arid southern Gobi region and Djungar Basin during the final second millennium BCE.

Finally, our data help to trace shifting cultural boundaries during the terminal Bronze Age, showing chronospatial segregation between first the prone/supine burial spheres, and the late persistence of deer stone features in northern Mongolia following the proliferation of slab burials across most of central Mongolia during the early first millennium BCE (Fig 5, bottom). At their zenith, deer stones were distributed across not only many areas of Mongolia but across the Sayan-Altai and Khangai mountains, reaching areas of Xinjiang, Tuva, and even Kyrgyzstan [51]. Small deer stones with the unique “Sayan-Altai” carving style found on the most recent Khuvsgul stones were included in the grand kurgans of Arzhan, in southern Tuva, dated to the same period [52]. Zazzo et al [39] have rightly pointed out that because deer stones are dated entirely through the faunal elements of their satellite features, their late persistence must be scrutinized for the possibility of radiocarbon contamination. It is noteworthy that the DSK burial types (khirigsuurs and Sagsai type) are largely superseded by slab burials, and that no dated slab burials have been identified in the pocket of northern Mongolia where deer stone features appear to persist during the early first millennium BCE (Fig 5I). Although further work must be done to assess the impact of taphonomy and diagenesis on this pattern, our analysis supports the suggestion of an emergent and northward-shifting cultural frontier between slab burial and deer stone-producing populations during the first millennium BCE.

Together, our model results present the first precision chronology for Mongolia’s early monumental burial traditions and their associated pastoralist subsistence evidence. While current datasets do not permit the application of robust quality control standards that would exclude the impacts of contamination and other diagenetic processes, our results support the chronological primacy of Afanasievo traditions and demonstrate a penetration of monumental burial traditions into the Mongolian steppe by the late 4th millennium BCE. Model results suggest an overlapping temporal succession of the earliest burial traditions (Chemurchek, Munkkhairkhan) in westerly areas of the region, following their initial appearance after Afanasievo migration. Our model provides a precision framework for the new burial traditions that appeared in the region toward the later second millennium BCE, and reveal important chronogeographic consistencies in burial position that cross-cut tradition cultural designations. The expansion of new prone and supine burial traditions overlapped key economic changes in the role of horses towards the final second millennium BCE, and future work using our chronological framework may help understand the causal impact of horses on LBA cultural and social dynamics. Future application of our multi-methodological approach, considering not only typology but also aspects of burial form and body position may help identify other meaningful cultural frontiers, in order to better understand monumental behaviors and social processes, and assess implications for emergent pastoralism in eastern Eurasia.

## Conclusion

Our analyses reveal Bayesian modeling as a crucial component of cultural history framework development in the investigation of pastoral origins and mortuary traditions in eastern Eurasia. Although increasing evidence is emerging for pastoral lifeways in Mongolia as early as 3000 BCE, ambiguity in cultural designations and lack of precision in published radiocarbon dates has made it difficult to assess early cultural dynamics in this region or assess their implications for the transition from hunting to herding economies. Our model results provide the first precision estimates for a number of Bronze Age burial traditions, helping to resolve the chronological sequence for the earliest pastoral-linked burial traditions within modern Mongolia. We provide a precise and empirical chronological model for the new wave of second millennium BCE burial traditions, demonstrating spatial and temporal patterns in burial traits that transect traditional typological categories and may help understand broader social dynamics during the emergence of pastoral societies in eastern Eurasia.

## Supporting information

S1 Appendix Radiocarbon dates from archaeological sites used in this study (includes dates excluded from final model because of failed chi-square tests, and oversampling as described in the methods section). Dates highlighted gray have published DC data (C/N ratio and collagen yield) that ensure reliability per Zazzo et al (2019), while those in dark gray have been included in the quality-control restricted model (light gray dates from Tsatsyn Ereg excluded because of oversampling).
(DOCX)

S2 Appendix OxCal code used in the analysis. (DOCX)

S1 Table New radiocarbon dates produced through this study. Specimens highlighted in dark gray fail both of the QC criteria recommended by Zazzo et al (2019), while those highlighted in light gray fail have a collagen yield below 5% but a C/N ratio of less than 3.3.
(DOCX)

S2 Table Model output for start boundaries, end boundaries, and average dates (“sum”) for each culture unit analyzed in this study, sorted according to median values. (DOCX)

S3 Table Model output for start boundaries, end boundaries, and average dates (“sum”) for each burial position unit analyzed in this study, sorted according to median values. (DOCX)
