# Supplementary material for: Radiocarbon dating and cultural dynamics across Mongolia’s early pastoral transition
Source: PLoS One. 2019 Nov 6;14(11):e0224241. doi: 10.1371/journal.pone.0224241 (PMC6834239; doi:10.1371/journal.pone.0224241)
Supplement: S1 Appendix — Dates highlighted gray have published DC data (C/N ratio and collagen yield) that ensure reliability per Zazzo et al (2019), while those in dark gray have been included in the quality-control restricted model (light gray dates from Tsatsyn Ereg excluded because of oversampling). (DOCX) [file pone.0224241.s001.docx]

**S1 Appendix.** Radiocarbon dates from archaeological sites used in this study (includes dates excluded from final model because of failed chi-square tests, and oversampling as described in the methods section). Dates highlighted gray have published DC data (C/N ratio and collagen yield) that ensure reliability per Zazzo et al (2019), while those in dark gray have been included in the quality-control restricted model (light gray dates from Tsatsyn Ereg excluded because of oversampling).

| **ID** | **^14^C Date Ref** | **^14^C Date (BP)** | **Uncertainty (σ)** | **Sample Material** | **Monument type** | **Reference** |
| --- | --- | --- | --- | --- | --- | --- |
| Bayan-Ulgii aimag, Ulaankhus sum, Khuurai Gobi 1 | Le-7219 | 4180 | 100 | Unspec. Bone | Afanasievo | Kovalev and Erdenebaatar 2009 |
|  |  |  |  |  |  |  |
| Bayankhongor aimag, Erdenetsogt sum, Shatar Chuluu, AT-26, Grave 2 | OxA-36222 | 4410 | 31 | Human bone | Afanasievo | This study |
| Bayankhongor aimag, Erdenetsogt sum, Shatar Chuluu, AT-25, Grave 5 | OxA-36221 | 4415 | 31 | Human tooth | Afanasievo | This study |
| Bayan-Ulgii aimag, Ulaankhus sum, Khundii Gobi AT-628, Kurgan 1, Burial 2 | OxA-36230 | 4114 | 29 | Bone (human) - rib | Chemurchek/Afanasievo | This study |
| Bayan-Ulgii aimag, Ulaankhus sum, Khuurai Gobi, AT-635, Kurgan 2 | GrM-12938 | 4034 | 16 | Human tooth | Chemurchek/Afanasievo | This study |
| Bayan-Ulgii aimag, Ulaankhus sum, Khul Uul | Le-7220 | 3725 | 115 | Unspec. Bone | Chemurchek/Afanasievo | Kovalev and Erdenebaatar 2009 |
|  |  |  |  |  |  |  |
| Bayan-Ulgii aimag, Ulaankhus sum, Khuurai Gobi 2 | Le-7215 | 3825 | 70 | Unspec. Bone | Chemurchek/Afanasievo | Kovalev and Erdenebaatar 2009 |
|  |  |  |  |  |  |  |
| Khundii Gobi | Le-7212 | 3340 | 70 | Unspec. Bone | Chemurchek/Afanasievo | Kovalev and Erdenebaatar 2009 |
| Khovd aimag, Bulgan sum, Yagshiin Khuduu, AT-590B, Grave 1 | GrM-12984 | 3983 | 17 | Human tooth | Chemurchek | This study |
| Khovd aimag, Bulgan sum,Yagshiin Khuduu 1 | Le-6937 | 3790 | 120 | Unspec. Bone | Chemurchek | Kovalev and Erdenebaatar 2009 |
| Khovd aimag, Bulgan sum,Yagshiin Khuduu 2 | Le-6942 | 3880 | 100 | Unspec. Bone | Chemurchek | Kovalev and Erdenebaatar 2009 |
| Khovd aimag, Bulgan sum,Yagshiin Khuduu 3 | Le-6932 | 3770 | 60 | Unspec. Bone | Chemurchek | Kovalev and Erdenebaatar 2009 |
| Khovd aimag, Bulgan sum,Yagshiin Khuduu 3 | Le-6933 | 4000 | 80 | Unspec. Bone | Chemurchek | Kovalev and Erdenebaatar 2009 |
| Khovd aimag, Bulgan sum,Yagshiin Khuduu 3 | Le-6939 | 3800 | 70 | Unspec. Bone | Chemurchek | Kovalev and Erdenebaatar 2009 |
| Khovd aimag, Bulgan sum, Kheviin am 1 | Le-7217 | 3560 | 105 | Unspec. Bone | Chemurchek | Kovalev and Erdenebaatar 2009 |
| Khovd aimag, Bulgan sum, Kheviin am 1 | Le-7222 | 3440 | 120 | Unspec. Bone | Chemurchek | Kovalev and Erdenebaatar 2009 |
| Khovd aimag, Bulgan sum, Kheviin am 1 | Le-7224 | 3800 | 200 | Unspec. Bone | Chemurchek | Kovalev and Erdenebaatar 2009 |
| Khovd aimag, Bulgan sum, Kheviin am 2 | Le-7214 | 3830 | 120 | Unspec. Bone | Chemurchek | Kovalev and Erdenebaatar 2009 |
| Khovd aimag, Bulgan sum, Buural Khariin Ar | Le-7225 | 4250 | 500 | Unspec. Bone | Chemurchek | Kovalev and Erdenebaatar 2009 |
|  |  |  |  |  |  |  |
| Khuvsgul aimag, Tsaganuur sum, Khogorgo-3 | Not reported | 3450 | 50 | Human tooth | Munkkhairkhan | Fitzhugh and Bayarsaikhan 2010 |
|  |  |  |  |  |  |  |
| Khovd aimag, Munkhkhairkhan sum, Ulaan Goviin Uzuur 1 | Le-6941 | 3310 | 90 | Unspec. Bone | Munkkhairkhan | Kovalev and Erdenebaatar 2009 |
|  |  |  |  |  |  |  |
| Khovd aimag, Munkhkhairkhan sum, Ulaan Goviin Uzuur 2 | Le-6636 | 3150 | 70 | Unspec. Bone | Munkkhairkhan | Kovalev and Erdenebaatar 2009 |
|  |  |  |  |  |  |  |
| Khovd aimag, Munkhkhairkhan sum, Ulaan Goviin Uzuur AT-614, Grave 2 | OxA-X-2737-53 | 3412 | 32 | Human tooth | Munkkhairkhan | Kovalev and Erdenebaatar 2009 |
|  |  |  |  |  |  |  |
| Khovd aimag, Munkhkhairkhan sum, Khotuu Davaa 1 | Le-6935 | 3270 | 60 | Unspec. Bone | Munkkhairkhan | Kovalev and Erdenebaatar 2009 |
|  |  |  |  |  |  |  |
| Khovd aimag, Munkhkhairkhan sum, Artua | Le-6934 | 3480 | 90 | Unspec. Bone | Munkkhairkhan | Kovalev and Erdenebaatar 2009 |
|  |  |  |  |  |  |  |
| Khovd aimag, Munkhkhairkhan sum, Shar Gobi 3, AT-960, Kurgan 1 | OxA-36455 | 3108 | 31 | Human tooth | Munkkhairkhan | Kovalev and Erdenebaatar 2009 |
|  |  |  |  |  |  |  |
| Dundgovie aimag, Delgertsogt sum. Baga Gazaryn Chuluu, Baga Mongol EX 07.23 | Not reported | 2990 | 40 | Human bone | Ulaanzuukh | Nelson et al. 2009 |
| Sukhbaatar aimag, Tuvshinshiree sum, Ulaanzuukh At-823, Grave 2 | OxA-36231 | 3028 | 25 | Human bone | Ulaanzuukh | This study |
| Sukhbaatar aimag, Tuvshinshiree sum, Ulaanzuukh At-769, Grave 42 | OxA-36459 | 3215 | 40 | Human tooth | Ulaanzuukh | This study |
| Sukhbaatar aimag, Tuvshinshiree sum, Ulaanzuukh At-824, Grave 1 | OxA-36460 | 3110 | 31 | Human bone | Ulaanzuukh | This study |
| Sukhbaatar aimag, Tuvshinshiree sum, Ulaanzuukh AT-921, Grave 33 | OxA-36232 | 3075 | 27 | Human bone | Ulaanzuukh | This study |
| Sukhbaatar aimag, Tuvshinshiree sum, Ulaanzuukh Burial 6 | IAAA-103372 | 3127 | 29 | Human bone | Ulaanzuukh | Tumen et al. 2012 |
| Sukhbaatar aimag, Tuvshinshiree sum, Ulaanzuukh Burial 2 | IAAA-103374 | 3115 | 28 | Human bone | Ulaanzuukh | Tumen et al. 2012 |
| Sukhbaatar aimag, Tuvshinshiree sum, Ulaanzuukh Burial A | IAAA-103369 | 3101 | 30 | Human bone | Ulaanzuukh | Tumen et al. 2012 |
| Sukhbaatar aimag, Tuvshinshiree sum, Ulaanzuukh Burial B | IAAA-103368 | 3082 | 31 | Human bone | Ulaanzuukh | Tumen et al. 2012 |
| Sukhbaatar aimag, Tuvshinshiree sum, Ulaanzuukh Burial C | IAAA-103370 | 3054 | 29 | Human bone | Ulaanzuukh | Tumen et al. 2012 |
| Sukhbaatar aimag, Tuvshinshiree sum, Ulaanzuukh Burial D | IAAA-103371 | 3015 | 28 | Human bone | Ulaanzuukh | Tumen et al. 2012 |
| Sukhbaatar aimag, Tuvshinshiree sum, Ulaanzuukh Burial 3 | IAAA-103373 | 3006 | 30 | Human bone | Ulaanzuukh | Tumen et al. 2012 |
| Sukhbaatar aimag, Delgerkh sum  CKU burial 33 | AA100870 | 3092 | 52 | Bone | Ulaanzuukh | Amartuvshin *et al*. 2015 |
| Sukhbaatar aimag, Tuvshinshiree sum  DMS 657a | UGAMS  28309 | 2930 | 30 | Bone | Ulaanzuukh | Wright *et al.* 2019 |
| Sukhbaatar aimag, Tuvshinshiree sum  DMS 657c | UGAMS  28310 | 2880 | 25 | Tooth | Ulaanzuukh | Wright *et al.* 2019 |
| Sukhbaatar aimag, Delgerkh sum  CKU Burial 2-2004 | OS-68952 | 3230 | 40 | Bone | Ulaanzuukh | Amartuvshin *et al*. 2015 |
| Sukhbaatar aimag, Delgerkh sum  CKU Burial 4-2004 | OS-68953 | 3170 | 40 | Bone | Ulaanzuukh | Amartuvshin *et al*. 2015 |
| Sukhbaatar aimag, Delgerkh sum  CKU Burial 31 | AA100860 | 3100 | 55 | Bone | Ulaanzuukh | Amartuvshin *et al*. 2015 |
| Sukhbaatar aimag, Delgerkh sum  CKU Burial 41 | AA100861 | 3057 | 52 | Bone | Ulaanzuukh | Amartuvshin *et al*. 2015 |
| Dundgovi aimag  Adaatsag sum  Baga Mongol 07.23 | NA | 2990 | 40 | Bone | Ulaanzuukh | Nelson et al. 2009 |
| Sukhbaatar aimag, Delgerkh sum  CKU Burial 211 | AA100863 | 3065 | 41 | Bone | Ulaanzuukh | Amartuvshin *et al*. 2015 |
| Bayan Ulgii aimag, Tsengel sum, Khoton Nuur East Bay 4 | Not reported | 3080 | 30 | Unspec. Bone | Sagsai | Fitzhugh et al 2012 |
| Bayankhongor aimag, Bogd sum, Bor Ovoo HG-2 | PLD-26017 | 2023 | 23 | Human bone | Sagsai | Miyamoto 2017 |
|  |  |  |  |  |  |  |
| Khovd aimag, Uyench sum, Uliastai River I, AT-676, Kurgan 1, burial 4 (main) | OxA-X-2737-54 | 2967 | 31 | Human tooth | Sagsai | This study |
| Uvurkhangai aimag,  Khujirt sum  Maikhan Tolgoi Gr 52 | NA | 2970 | 35 | Bone | Sagsai | Bemmann et al. 2015 |
| Bayankhongor aimag, Bayanlig sum, Zamyn Buts Secondary Burial | Le-7966 | 2980 | 110 | Unspec. Bone | D-shape | Kovalev and Erdenebaatar 2009 |
|  |  |  |  |  |  |  |
| Bayankhongor aimag, Bayanlig sum, Baruun Gyalaat 2 | Le-7954 | 2900 | 50 | Unspec. Bone | D-shape | Kovalev and Erdenebaatar 2009 |
|  |  |  |  |  |  |  |
| Uvurkhangai aimag, Khujirt sum, Shorgooljiin Bulsh 3 | Not reported | 3343 | 38 | Human bone | Shape | Eruul-Erdene et al. 2015 |
|  |  |  |  |  |  |  |
| Bayankhogor aimag, Ulziit sum, Shape burial | AA108307 | 2482 | 27 | Horse tooth | Shape | ??? |
|  |  |  |  |  |  |  |
| Uverkhangai aimag, Bogd sum, Tevsh Uul 1 Burial 1 | PLD-23379 | 2706 | 20 | Human bone | Shape burial | Miyamoto and Obata 2016 |
|  |  |  |  |  |  |  |
| Uverkhangai aimag, Bogd sum, Tevsh Uul 3 Burial 1 Layer 3 | PLD-13381 | 3050 | 20 | Human bone | Shape burial | Miyamoto and Obata 2016 |
|  |  |  |  |  |  |  |
| Uverkhangai aimag, Bogd sum, Tevsh Uul 4 Burial 5 | PLD-23382 | 2912 | 20 | Human bone | Shape burial | Miyamoto and Obata 2016 |
|  |  |  |  |  |  |  |
| Uvurkhangai aimag, Khujirt sum, Khirigsuur 12.9 | Not reported | 2887 | 38 | Horse bone | Khirigsuur | Eruul-Erdene et al. 2015 |
|  |  |  |  |  |  |  |
| Khovd aimag, Mankhan sum, Berkh Mountain, AT-905 Khirigsuur 3 | OxA-36255 | 2989 | 28 | Human tooth | Khirigsuur | This study |
| Khovsgol aimag, Alag-Erdene sum, Ulaan Tolgoi, AT-617 | OxA-36233 | 2921 | 28 | Human tooth | Khirigsuur | This study |
| Dundgovi aimag, Delgertsogt sum, Baga Gazaryn Chuluu EX 08.21 | SUERC-27257 | 3050 | 30 | Human bone | Khirigsuur? | Machicek 2011 |
|  |  |  |  |  |  |  |
| Gobi Altai aimag, Tonkhil sum, Kharyn Kharaach-4 | TKA-16563 | 2982 | 43 | Human bone | Khirigsuur | Miyamoto 2017 |
|  |  |  |  |  |  |  |
| Bayankhongor aimag, Bogd sum, Bor Ovoo HG-12 | PLD-26019 | 3054 | 21 | Human bone | Khirigsuur | Miyamoto 2017 |
|  |  |  |  |  |  |  |
| Uvurkhangai aimag, Khujirt sum, Khirigsuur 12.3 | COL2032.1.1 | 2887 | 38 | Horse bone | Khirigsuur | Yeruul-Erdene et al. 2015 |
| Uvurkhangai aimag, Khujirt sum, Khirigsuur 4.32 | KIA-49219 | 2880 | 25 | Horse bone | Khirigsuur | Yeruul-Erdene et al. 2015 |
| Khuvsgul aimag, 54.22 | Not reported | 2843 | 41 | Horse bone | Khirigsuur | Frohlich et al 2009 |
| Khuvsgul aimag, S49.2 | Not reported | 2730 | 50 | Horse bone | Khirigsuur | Frohlich et al 2009 |
| Uvs aimag, Zuunkhangai sum, ZK-1-1 | AA106955 | 2963 | 31 | Horse tooth | Khirigsuur | Taylor 2017 (courtesy of J-L Houle) |
| Bayankhongor aimag, Erdenetsogt sum, Shatar Chuluu KS1 | AA106951 | 2955 | 31 | Horse tooth | Khirigsuur | Taylor 2017 |
| Khuvsgul aimag, Alag-Erdene sum, Ulaan Tolgoi KS M1 F3 tooth | B-215693 AMS | 2950 | 60 | Horse tooth | Khirigsuur | Fitzhugh and Bayarsaikhan 2009 |
| Khuvsgul, Zunii Gol, K3 F42 | B-272759 AMS | 2950 | 40 | Horse tooth | Khirigsuur | Fitzhugh and Bayarsaikhan 2009 |
| Khuvsgul aimag, Renchinlkhumbe sum, Zeerdegchingiin Khoshuu FA | AA106945 | 2934 | 31 | Horse tooth | Khirigsuur | Taylor 2017 |
| Bayan-Ulgii aimag, Tsengel sum, On Khad Khushuu | B-246613 AMS | 2930 | 40 | Horse tooth | Khirigsuur | Fitzhugh and Bayarsaikhan 2009 |
| Uvs aimag, Zuunkhangai sum, ZK-1-3 | AA106956 | 2922 | 30 | Horse tooth | Khirigsuur | Taylor 2017 (courtesy of J-L Houle) |
| Bulgan aimag, Khutag-Undur sum, Uurgiin Gol KS 64-4 | AA106952 | 2903 | 30 | Horse tooth | Khirigsuur | Taylor 2017 |
| Khuvsgul aimag, Alag-Erdene sum, Ulaan Tolgoi KS Mound 1 F2 tooth | B-215692 AMS | 2860 | 40 | Horse tooth | Khirigsuur | Fitzhugh and Bayarsaikhan 2009 |
| Uvs aimag, Zuunkhangai sum, ZK-257-4 | AA106958 | 2850 | 29 | Horse tooth | Khirigsuur | Taylor 2017 (courtesy of J-L Houle) |
| Uvs aimag, Zuunkhangai sum, ZK-257-1 | AA106957 | 2836 | 30 | Horse tooth | Khirigsuur | Taylor 2017 (courtesy of J-L Houle) |
| Arkhangai aimag, Khanuy Valley, Urt Bulagyn KYRI 22 tooth | B-222533 AMS | 2790 | 40 | Horse tooth | Khirigsuur | Fitzhugh and Bayarsaikhan 2009 |
| Arkhangai aimag, Undur-Ulaan sum, Urt Bulagyn KYRI 21 tooth | B-222532 AMS | 2780 | 50 | Horse tooth | Khirigsuur | Fitzhugh and Bayarsaikhan 2009 |
| Khuvsgul aimag, Galt sum, Nukhtiin Am M1 F1 tooth | B-240685 AMS | 2630 | 40 | Horse tooth | Khirigsuur | Fitzhugh and Bayarsaikhan 2009 |
| Arkhangai aimag, Tsatsyn Ereg, B10- ST101 | ECHo 1475 | 2845 | 20 | Indet. animal bone | Khirigsuur | Zazzo et al 2019 |
| Arkhangai aimag, Tsatsyn Ereg, B10- ST102 | ECHo 1489 | 2825 | 20 | Indet. animal bone | Khirigsuur | Zazzo et al 2019 |
| Arkhangai aimag, Tsatsyn Ereg, B10- ST103 | ECHo 1483 | 2855 | 20 | Indet. animal bone | Khirigsuur | Zazzo et al 2019 |
| Arkhangai aimag, Tsatsyn Ereg, B10- ST104 | ECHo 1485 | 2845 | 20 | Indet. animal bone | Khirigsuur | Zazzo et al 2019 |
| Arkhangai aimag, Tsatsyn Ereg, B10- ST107 | ECHo 1487 | 2805 | 20 | Indet. animal bone | Khirigsuur | Zazzo et al 2019 |
| Arkhangai aimag, Tsatsyn Ereg, B10- ST108 | ECHo 1472 | 2805 | 20 | Indet. animal bone | Khirigsuur | Zazzo et al 2019 |
| Arkhangai aimag, Tsatsyn Ereg, B10- ST109 | ECHo 1474 | 2810 | 20 | Indet. animal bone | Khirigsuur | Zazzo et al 2019 |
| Arkhangai aimag, Tsatsyn Ereg, B10- ST110 | ECHo 1478 | 2790 | 20 | Indet. animal bone | Khirigsuur | Zazzo et al 2019 |
| Arkhangai aimag, Tsatsyn Ereg, B10- ST105 | ECHo 1477 | 2845 | 20 | Indet. animal bone | Khirigsuur | Zazzo et al 2019 |
| Arkhangai aimag, Tsatsyn Ereg, B10- ST111 | ECHo 1476 | 2855 | 20 | Indet. animal bone | Khirigsuur | Zazzo et al 2019 |
| Arkhangai aimag, Tsatsyn Ereg, B10- ST112 | ECHo 1484 | 2845 | 20 | Indet. animal bone | Khirigsuur | Zazzo et al 2019 |
| Arkhangai aimag, Tsatsyn Ereg, B10- ST113 | ECHo 1480 | 2860 | 20 | Indet. animal bone | Khirigsuur | Zazzo et al 2019 |
| Arkhangai aimag, Tsatsyn Ereg, B10- ST114 | ECHo 1488 | 2785 | 20 | Indet. animal bone | Khirigsuur | Zazzo et al 2019 |
| Arkhangai aimag, Tsatsyn Ereg, B10- ST115 | ECHo 1479 | 2825 | 20 | Indet. animal bone | Khirigsuur | Zazzo et al 2019 |
| Arkhangai aimag, Tsatsyn Ereg, B10- ST116 | ECHo 1486 | 2805 | 20 | Indet. animal bone | Khirigsuur | Zazzo et al 2019 |
| Arkhangai aimag, Tsatsyn Ereg, B10- ST117 | ECHo 1482 | 2850 | 20 | Indet. animal bone | Khirigsuur | Zazzo et al 2019 |
| Arkhangai aimag, Tsatsyn Ereg, B10- ST118 | ECHo 1470 | 2770 | 20 | Indet. animal bone | Khirigsuur | Zazzo et al 2019 |
| Arkhangai aimag, Tsatsyn Ereg, B10- C476 | ECHo 1481 | 2830 | 20 | Indet. animal bone | Khirigsuur | Zazzo et al 2019 |
| Arkhangai aimag, Tsatsyn Ereg, B10- C531 | ECHo 1472 | 2840 | 20 | Indet. animal bone | Khirigsuur | Zazzo et al 2019 |
| Arkhangai aimag, Tsatsyn Ereg, B10- C1162 | ECHo 1473 | 2855 | 20 | Indet. animal bone | Khirigsuur | Zazzo et al 2019 |
| Arkhangai aimag, Tsatsyn Ereg, B10- ST1 | ECHo 1416 | 2810 | 25 | Horse bone | Khirigsuur | Zazzo et al 2019 |
| Arkhangai aimag, Tsatsyn Ereg, B10- ST 2 | ECHo 1804.1.1 | 2845 | 25 | Horse tooth | Khirigsuur | Zazzo et al 2019 |
| Arkhangai aimag, Tsatsyn Ereg, B10- ST 3 | ECHo 1418 | 2915 | 25 | Horse bone | Khirigsuur | Zazzo et al 2019 |
| Arkhangai aimag, Tsatsyn Ereg, B10- ST 5* | ECHo 1439 | 2840 | 25 | Horse tooth | Khirigsuur | Zazzo et al 2019 |
| Arkhangai aimag, Tsatsyn Ereg, B10- ST 5 | Echo 1684.1.1 | 2870 | 25 | Horse tooth | Khirigsuur | Zazzo et al 2019 |
| Arkhangai aimag, Tsatsyn Ereg, B10- ST 5* | ECHo 1438 | 2850 | 25 | Horse bone | Khirigsuur | Zazzo et al 2019 |
| Arkhangai aimag, Tsatsyn Ereg, B10- ST 8 | ECHo 1805.1.1 | 2850 | 25 | Horse tooth | Khirigsuur | Zazzo et al 2019 |
| Arkhangai aimag, Tsatsyn Ereg, B10- ST 9 | ECHo 1677.1.1 | 2840 | 25 | Horse bone | Khirigsuur | Zazzo et al 2019 |
| Arkhangai aimag, Tsatsyn Ereg, B10- ST 10 | ECHo 1422 | 2780 | 25 | Horse tooth | Khirigsuur | Zazzo et al 2019 |
| Arkhangai aimag, Tsatsyn Ereg, B10- ST 11* | ECHo 1441 | 2745 | 25 | Horse tooth | Khirigsuur | Zazzo et al 2019 |
| Arkhangai aimag, Tsatsyn Ereg, B10- ST11* | ECHo 1685.1.1 | 2805 | 25 | Horse bone | Khirigsuur | Zazzo et al 2019 |
| Arkhangai aimag, Tsatsyn Ereg, B10- ST12 | ECHo 1424 | 2835 | 20 | Horse tooth | Khirigsuur | Zazzo et al 2019 |
| Arkhangai aimag, Tsatsyn Ereg, B10- ST 14 | ECHo 1806.1.1 | 2855 | 25 | Horse tooth | Khirigsuur | Zazzo et al 2019 |
| Arkhangai aimag, Tsatsyn Ereg, B10- ST 15 | ECHo 1807.1.1 | 2875 | 25 | Horse tooth | Khirigsuur | Zazzo et al 2019 |
| Arkhangai aimag, Tsatsyn Ereg, B10- ST 16 | ECHo 1808.1.1 | 2825 | 25 | Horse tooth | Khirigsuur | Zazzo et al 2019 |
| Arkhangai aimag, Tsatsyn Ereg, B10- ST 17* | ECHo 1415 | 2805 | 25 | Horse tooth | Khirigsuur | Zazzo et al 2019 |
| Arkhangai aimag, Tsatsyn Ereg, B10- ST 17 | ECHo 1686.1.1 | 2905 | 25 | Horse tooth | Khirigsuur | Zazzo et al 2019 |
| Arkhangai aimag, Tsatsyn Ereg, B10- ST 18 | ECHo 1809.1.1 | 2860 | 25 | Horse tooth | Khirigsuur | Zazzo et al 2019 |
| Arkhangai aimag, Tsatsyn Ereg, B10- SAT 354 | ECHo 1798.1.1 | 2880 | 25 | Horse tooth | Khirigsuur | Zazzo et al 2019 |
| Arkhangai aimag, Tsatsyn Ereg, B10- SAT 354* | SacA39453 | 2845 | 30 | Horse tooth | Khirigsuur | Zazzo et al 2019 |
| Arkhangai aimag, Tsatsyn Ereg, B10- SAT 397 | ECHo 1799.1.1 | 2900 | 25 | Horse tooth | Khirigsuur | Zazzo et al 2019 |
| Arkhangai aimag, Tsatsyn Ereg, B10- SAT 415 | ECHo 1800.1.1 | 2840 | 25 | Horse tooth | Khirigsuur | Zazzo et al 2019 |
| Arkhangai aimag, Tsatsyn Ereg, B10- SAT 416 | ECHo 1801.1.1 | 2865 | 30 | Horse tooth | Khirigsuur | Zazzo et al 2019 |
| Arkhangai aimag, Tsatsyn Ereg, B10- SAT 528 | ECHo 1679.1.1 | 2815 | 25 | Horse tooth | Khirigsuur | Zazzo et al 2019 |
| Arkhangai aimag, Tsatsyn Ereg, B10- SAT 666 | ECHo 1802.1.1 | 2820 | 25 | Horse tooth | Khirigsuur | Zazzo et al 2019 |
| Arkhangai aimag, Tsatsyn Ereg, B10- SAT 732 | SacA39454 | 2885 | 30 | Horse tooth | Khirigsuur | Zazzo et al 2019 |
| Arkhangai aimag, Tsatsyn Ereg, B10- SAT 799 | ECHo 1803.1.1 | 2845 | 30 | Horse tooth | Khirigsuur | Zazzo et al 2019 |
| Arkhangai aimag, Tsatsyn Ereg, B10- SAT 810 | ECHo 1497 | 2835 | 25 | Horse tooth | Khirigsuur | Zazzo et al 2019 |
| Arkhangai aimag, Tsatsyn Ereg, B10- SAT 810 | ECHo 1681.1.1 | 2850 | 25 | Horse tooth | Khirigsuur | Zazzo et al 2019 |
| Arkhangai aimag, Tsatsyn Ereg, B10- SAT 811* | ECHo 1499 | 2805 | 20 | Horse tooth | Khirigsuur | Zazzo et al 2019 |
| Arkhangai aimag, Tsatsyn Ereg, B10- SAT 811 | ECHo 1682.1.1 | 2865 | 25 | Horse bone | Khirigsuur | Zazzo et al 2019 |
| Arkhangai aimag, Tsatsyn Ereg, B10- SAT 811* | ECHo 1683.1.1 | 2835 | 25 | Horse tooth | Khirigsuur | Zazzo et al 2019 |
| Arkhangai aimag, Tsatsyn Ereg, B10- SAT 1023 | ECHo 1796.1.1 | 2865 | 25 | Horse tooth | Khirigsuur | Zazzo et al 2019 |
| Arkhangai aimag, Tsatsyn Ereg, B10- SAT 1023* | ECHo 1797.1.1 | 2840 | 25 | Horse tooth | Khirigsuur | Zazzo et al 2019 |
| Arkhangai aimag, Tsatsyn Ereg, KTS01-S2 | ECHo 1811.1.1 | 2880 | 25 | Horse tooth | Khirigsuur | Zazzo et al 2019 |
| Arkhangai aimag, Tsatsyn Ereg, KTS01-S5 | ECHo 1814.1.1 | 2890 | 25 | Horse tooth | Khirigsuur | Zazzo et al 2019 |
| Khuvsgul aimag, Burentogtokh sum, Ulaan Ushig 3 (SC 5) | MTC-12815 | 2923 | 59 | Unspec. horse | Deer Stone | Hayashi 2013 |
| Khuvsgul aimag, Burentogtokh sum, Ulaan Ushig 1 (SC 7) | MTC-12531 | 2749 | 50 | Unspec. horse | Deer Stone | Hayashi 2013 |
| Dundgovi aimag, Baga Gazaryn Chuluu, EX 04.04 | OS-71705 | 3040 | 35 | Unspec. horse | Khirigsuur | Amartuvshin and Jargalan 2010 |
| Dundgovi aimag, Baga Gazaryn Chuluu, EX 04.04 | OS-71705 | 3060 | 35 | Unspec. horse | Khirigsuur | Amartuvshin and Jargalan 2010 |
| Khuvsgul aimag, Burentogtokh sum, Ulaan Ushig 2 (Kh1 SH-18) | MTC-12814 | 2871 | 59 | Unspec. horse | Khirigsuur | Hayashi 2013 |
| Bulgan aimag, Tarvagtai Valley, Site 2 Feature 2 | UG-18415 | 2810 | 25 | Unspec. horse | Khirigsuur | B. Jargalan, unpublished |
| Bulgan aimag, Tarvagtai Valley, Site 2 Feature 1 | UG-18414 | 2780 | 20 | Unspec. horse | Khirigsuur | B. Jargalan, unpublished |
| Khuvsgul aimag, 24 | Not reported | 3174 | 53 | Human bone | Khirigsuur | Frohlich et al 2009 |
| Khuvsgul aimag, 51 | Not reported | 3086 | 41 | Human bone | Khirigsuur | Frohlich et al 2009 |
| Khuvsgul aimag, 18 | Not reported | 3074 | 49 | Human bone | Khirigsuur | Frohlich et al 2009 |
| Khuvsgul aimag, 58 | Not reported | 3056 | 46 | Human bone | Khirigsuur | Frohlich et al 2009 |
| Khuvsgul aimag, 25 | Not reported | 3052 | 50 | Human bone | Khirigsuur | Frohlich et al 2009 |
| Khuvsgul aimag, 23 | Not reported | 3052 | 52 | Human bone | Khirigsuur | Frohlich et al 2009 |
| Khuvsgul aimag, 40 | Not reported | 3052 | 51 | Human bone | Khirigsuur | Frohlich et al 2009 |
| Khuvsgul aimag, 3 | Not reported | 3044 | 50 | Human bone | Khirigsuur | Frohlich et al 2009 |
| Khuvsgul aimag, 22 | Not reported | 3033 | 49 | Human bone | Khirigsuur | Frohlich et al 2009 |
| Khuvsgul aimag, 17 | Not reported | 3029 | 49 | Human bone | Khirigsuur | Frohlich et al 2009 |
| Khuvsgul aimag, 10 | Not reported | 2992 | 48 | Human bone | Khirigsuur | Frohlich et al 2009 |
| Khuvsgul aimag, 9 | Not reported | 2991 | 48 | Human bone | Khirigsuur | Frohlich et al 2009 |
| Khuvsgul aimag, 55 | Not reported | 2990 | 38 | Human bone | Khirigsuur | Frohlich et al 2009 |
| Khuvsgul aimag, 13 | Not reported | 2989 | 48 | Human bone | Khirigsuur | Frohlich et al 2009 |
| Khuvsgul aimag, 41 | Not reported | 2958 | 42 | Human bone | Khirigsuur | Frohlich et al 2009 |
| Khuvsgul aimag, 16 | Not reported | 2930 | 50 | Human bone | Khirigsuur | Frohlich et al 2009 |
| Khuvsgul aimag, 44 | Not reported | 2918 | 51 | Human bone | Khirigsuur | Frohlich et al 2009 |
| Khuvsgul aimag, 2 | Not reported | 2910 | 52 | Human bone | Khirigsuur | Frohlich et al 2009 |
| Khuvsgul aimag, Soyo S49 | Not reported | 2900 | 50 | Human bone | Khirigsuur | Frohlich et al 2009 |
| Khuvsgul aimag, 7 | Not reported | 2897 | 55 | Human bone | Khirigsuur | Frohlich et al 2009 |
| Khuvsgul aimag, 8 | Not reported | 2872 | 48 | Human bone | Khirigsuur | Frohlich et al 2009 |
| Khuvsgul aimag, 43 | Not reported | 2862 | 51 | Human bone | Khirigsuur | Frohlich et al 2009 |
| Khuvsgul aimag, 6 | Not reported | 2857 | 54 | Human bone | Khirigsuur | Frohlich et al 2009 |
| Khuvsgul aimag, 14 | Not reported | 2849 | 49 | Human bone | Khirigsuur | Frohlich et al 2009 |
| Khuvsgul aimag, 52 | Not reported | 2842 | 42 | Human bone | Khirigsuur | Frohlich et al 2009 |
| Khuvsgul aimag, 27 | Not reported | 2835 | 50 | Human bone | Khirigsuur | Frohlich et al 2009 |
| Khuvsgul aimag, 54 | Not reported | 2831 | 41 | Human bone | Khirigsuur | Frohlich et al 2009 |
| Khuvsgul aimag, 1 | Not reported | 2779 | 50 | Human bone | Khirigsuur | Frohlich et al 2009 |
| Uvs aimag, Zuunkhangai, SKT-B-1 | AA106959 | 3142 | 30 | Human tooth | Khirigsuur | This study |
| Bayan-Ulgii aimag, Tsengel sum, Khuiten Gol Delta 2 KS | B-334573 | 2800 | 30 | Human tooth | Khirisguur | Fitzhugh et al. 2013 |
| Bayankhongor aimag, Erdenetsogt sum, Bor Shoroonii Am KS 1 | AA106949 | 2871 | 31 | Sheep tooth | Khirigsuur | Taylor 2017 |
| Dundgovi aimag, Baga Gazaryn Chuluu, EX 07.24 | OS-68948 | 3060 | 35 | Unspec. bone | Khirigsuur | Amartuvshin and Jargalan 2010 |
| Uvurkhangai aimag, Khujirt sum, Khirigsuur 4.11 | KA-49218 | 2830 | 40 | Unspec. bone | Khirigsuur | Yeruul-Erdene et al. 2015 |
| Khovd aimag, Uyench sum, Uliastai River (lower terrace) I, AT-677, Kurgan 4 | Le-7219 | 2805 | 16 | Human tooth | Baitag | This study |
|  |  |  |  |  |  |  |
| Khovd aimag, Bulgan sum, Kheviin am | Le-7223 | 2910 | 90 | Unspec. Bone | Baitag | Kovalev and Erdenebaatar 2009 |
|  |  |  |  |  |  |  |
| Khovd aimag, Uyench sum, Uliastai Zastav II, AT-674, Kurgan 2 | OxA-36224 | 2824 | 28 | Human teeth | Baitag | This study |
| Khuvsgul aimag, Alag-Erdene sum, Ulaan Tolgoi DS4 F2 | B-193739 AMS | 2950 | 40 | Horse bone | Deer Stone | Fitzhugh and Bayarsaikhan 2009 |
| Arkhangai aimag, Ulaan Tolgoin Ar Shil, Deer Stone 62 Feature 17 | B-389401 | 2880 | 30 | Horse bone | Deer Stone | Gantulga et al. 2016 |
| Khuvsgul aimag, Alag-Erdene sum, Ulaan Tolgoi DS4 F3 | B-193740 AMS | 2810 | 40 | Horse bone | Deer Stone | Fitzhugh and Bayarsaikhan 2009 |
| Khuvsgul aimag, Alag-Erdene sum, Ulaan Tolgoi DS4 F5 | B-207205 RAD | 2790 | 70 | Horse bone | Deer Stone | Fitzhugh and Bayarsaikhan 2009 |
| Arkhangai aimag, Khavtsaliin Am, Deer Stone 40 Feature 4 | B-389402 | 2780 | 30 | Horse bone | Deer Stone | Gantulga et al. 2016 |
| Khuvsgul aimag, Alag-Erdene sum, Ulaan Tolgoi DS4 F6 | B-207206 RAD | 2740 | 70 | Horse bone | Deer Stone | Fitzhugh and Bayarsaikhan 2009 |
| Khuvsgul aimag, Alag-Erdene sum, Khyadag W DS1 F1 | B-246623 AMS | 2610 | 40 | Horse bone | Deer Stone | Fitzhugh and Bayarsaikhan 2009 |
| Khuvsgul aimag, Alag-Erdene sum, Khyadag E DS pav 7 | Not reported | 2610 | 40 | Horse tooth | Deer Stone | Taylor et al. 2017 |
| Khuvsgul aimag, Alag-Erdene sum, Khyadag E A3 F32 | Not reported | 2520 | 40 | Horse tooth | Deer Stone | Taylor et al. 2017 |
| Khuvsgul aimag, Alag-Erdene sum, Ulaan Tolgoi DS4 F1 | B-193738 AMS | 2530 | 40 | Horse bone | Deer Stone | Fitzhugh and Bayarsaikhan 2009 |
| Khuvsgul aimag, Bayanzurkh sum, Khorigiin Am F1 | AA106947 | 2438 | 33 | Horse bone | Deer Stone | Taylor 2017 |
| Arkhangai aimag, Bayantsagaanii Khundii, Deer Stone 38 Feature 1 | ECHo 1816.1.1 | 2860 | 25 | Horse tooth | Deer Stone | Zazzo et al. 2019 |
| Arkhangai aimag, Bayantsagaanii Khundii, Deer Stone 38 Feature 95 | ECHo 1818.1.1 | 2840 | 25 | Horse tooth | Deer Stone | Zazzo et al 2019 |
| Arkhangai aimag, Bayantsagaanii Khundii, Deer Stone 38 Feature 27 | ECHo 1817.1.1 | 2840 | 25 | Horse tooth | Deer Stone | Zazzo et al 2019E |
| Khuvsgul aimag, Renchinlkhumbe sum, Tstatstain Khushuu DS1 F2 | B-207207 AMS | 3000 | 40 | Horse tooth | Deer Stone | Fitzhugh and Bayarsaikhan 2009 |
| Bayankhongor aimag, Erdenetsogt sum, Bor Shoroonii Am DS F1 | AA106948 | 2977 | 30 | Horse tooth | Deer Stone | Taylor 2017 |
| Bayankhongor aimag, Erdenetsogt sum, Shatar Chuluu DS 1 | AA106950 | 2953 | 31 | Horse tooth | Deer Stone | Taylor 2017 |
| Khuvsgul aimag, Renchinlkhumbe sum, Tstatstain Khushuu DS 1 F1 | B-207208 AMS | 2920 | 40 | Horse tooth | Deer Stone | Fitzhugh and Bayarsaikhan 2009 |
| Khuvsgul aimag, Tsagaan Uul sum, Khushuutiin Gol A3 F3 | B-246618 AMS | 2910 | 40 | Horse tooth | Deer Stone | Fitzhugh and Bayarsaikhan 2009 |
| Khuvsgul aimag, Galt sum, Khushuutiin Am F18 | B-272763 AMS | 2880 | 40 | Horse tooth | Deer Stone | Fitzhugh and Bayarsaikhan 2009 |
| Khuvsgul aimag, Tumurbulag sum, Zunii Gol A1 F3 | B-272756 AMS | 2870 | 40 | Horse tooth | Deer Stone | Fitzhugh and Bayarsaikhan 2009 |
| Khuvsgul aimag, Tumurbulag sum, Zunii Gol A3 F1 | B-272758 AMS | 2860 | 40 | Horse tooth | Deer Stone | Fitzhugh and Bayarsaikhan 2009 |
| Bayankhongor aimag, Erdenetsogt sum, Shatar Chuluu DS 2 | AA106953 | 2846 | 30 | Horse tooth | Deer Stone | Taylor 2017 |
| Khuvsgul aimag, Burentogtokh sum, Ulaan Ushig 5 (Kh1 SH-11) | MTC-12817 | 2835 | 57 | Horse tooth | Deer Stone | Hayashi 2013 |
| Khuvsgul aimag, Alag-Erdene sum, Ulaan Tolgoi DS5 F2 | B-222535 AMS | 2830 | 40 | Horse tooth | Deer Stone | Fitzhugh and Bayarsaikhan 2009 |
| Khuvsgul aimag, Galt sum, Nukhtiin Am DS1/2 F1 | B-222534 AMS | 2830 | 40 | Horse tooth | Deer Stone | Fitzhugh and Bayarsaikhan 2009 |
| Khuvsgul aimag, Alag-Erdene sum, Ulaan Tolgoi DS5 F1 | B-215694 AMS | 2800 | 40 | Horse tooth | Deer Stone | Fitzhugh and Bayarsaikhan 2009 |
| Khuvsgul aimag, Renchinlkhumbe sum, Targan Nuur F1 | AA106946 | 2800 | 31 | Horse tooth | Deer Stone | Taylor 2017 |
| Khuvsgul aimag, Shin Ider sum, Tsokhiotin Am A1 DS2 F2 | B-272760 AMS | 2790 | 40 | Horse tooth | Deer Stone | Fitzhugh and Bayarsaikhan 2009 |
| Khuvsgul aimag, Tsagaan Uul sum, Khushuutiin Gol F2 | B-246617 AMS | 2750 | 40 | Horse tooth | Deer Stone | Fitzhugh and Bayarsaikhan 2009 |
| Khuvsgul aimag, Tumurbulag sum, Zunii Gol A2 DS4 | B-272757 AMS | 2710 | 40 | Horse tooth | Deer Stone | Fitzhugh and Bayarsaikhan 2009 |
| Zavkhan aimag, Telmen sum, Ogomoor DS8 f1 tooth | B-363204 | 2710 | 30 | Horse tooth | Deer Stone | Taylor 2017 |
| Khuvsgul aimag, Alag-Erdene sum, Khushuutiin Devseg F3 | B-243716 AMS | 2680 | 40 | Horse tooth | Deer Stone | Fitzhugh and Bayarsaikhan 2009 |
| Arkhangai aimag, Undur-Ulaan sum, Jargalantyn Am DS | Not reported | 2670 | 30 | Horse tooth | Deer Stone | Fitzhugh and Kortum 2012 |
| Khuvsgul aimag, Tsagaan Uul sum, Bor Hujiriin Gol A1 F2 | B-246614 AMS | 2640 | 40 | Horse tooth | Deer Stone | Fitzhugh and Bayarsaikhan 2009 |
| Zavkhan aimag, Shiluustei sum, Daagan Del F1 | B-363203 | 2620 | 30 | Horse tooth | Deer Stone | Taylor 2017 |
| Khuvsgul aimag, Alag-Erdene sum, Khushuutiin Devseg F2 | B-240688 AMS | 2450 | 40 | Horse tooth | Deer Stone | Fitzhugh and Bayarsaikhan 2009 |
| Khuvsgul aimag, Alag-Erdene sum, Khushuutiin Devseg F1 | B-243716 AMS | 2410 | 40 | Horse tooth | Deer Stone | Fitzhugh and Bayarsaikhan 2009 |
| Arkhangai aimag, Shivertiin Am, Deer Stone 33 Feature 6 | B-323805 | 2910 | 30 | Unspec. bone | Deer Stone | Gantulga et al. 2016 |
| Arkhangai aimag, Ikh Tamir, Monument 341 | B-290944 | 2810 | 40 | Unspec. bone | Deer Stone | Gantulga et al. 2016 |
| Dundgovi aimag, Delgertsogt sum, Baga Gazaryn Chuluu EX 07.07 | SUERC-27247 | 2340 | 30 | Human bone | Slab burial | Machicek 2011 |
|  |  |  |  |  |  |  |
| Dundgovi aimag, Delgertsogt sum, Baga Gazaryn Chuluu EX 07.01 | SUERC-27246 | 2745 | 30 | Human bone | Slab burial | Machicek 2011 |
|  |  |  |  |  |  |  |
| Uvurkhangai aimag, Khujirt sum, Duruvljin Bulsh 2 | Not reported | 2855 | 30 | Human bone | Slab burial | Eruul-Erdene et al. 2015 |
|  |  |  |  |  |  |  |
| Uvurkhangai aimag, Khujirt sum, Duruvljin Bulsh 13 | Not reported | 2430 | 35 | Unspec. bone | Slab burial | Eruul-Erdene et al. 2015 |
|  |  |  |  |  |  |  |
| Uvurkhangai aimag Khujirt sum, Duruvljin Bulsh 10 | Not reported | 2330 | 30 | Human bone | Slab burial | Eruul-Erdene et al. 2015 |
|  |  |  |  |  |  |  |
| Bayankhongor aimag, Bogd sum, Orog Nuur | LTL-1822A | 2866 | 55 | Unspec. bone | Slab burial | Gunchinsuren et al. 2006 |
|  |  |  |  |  |  |  |
| Khentii aimag, Delgerkhaan sum, Daram D048 | MTC-16081 | 2395 | 50 | Cattle bone | Slab burial | Miyamoto and Obata 2016 |
| Khentii aimag, Delgerkhaan sum, Daram D021-M1 | MTC-16083 | 2278 | 42 | Cattle bone | Slab burial | Miyamoto and Obata 2016 |
| Khentii aimag, Delgerkhaan sum, Daram 4-M4 burial A | MTC-16084 | 2487 | 50 | Human bone | Slab burial | Miyamoto and Obata 2016 |
| Khentii aimag, Delgerkhaan sum, Daram D010-M41 | MTC-14191 | 1924 | 41 | Cattle bone | Slab burial | Miyamoto and Obata 2016 |
| Khentii aimag, Delgerkhaan sum, Daram D012 M1 | MTC-14192 | 2368 | 37 | Horse bone | Slab burial | Miyamoto and Obata 2016 |
| Khentii aimag, Delgerkhaan sum, Daram D032 M2 | MTC-14193 | 2455 | 66 | Human bone | Slab burial | Miyamoto and Obata 2016 |
| Khentii aimag, Delgerkhaan sum, Daram D042 M1 coffin | MTC-14194 | 2350 | 46 | Horse bone | Slab burial | Miyamoto and Obata 2016 |
| Khentii aimag, Delgerkhaan sum, Daram D047 M1 coffin | MTC-14195 | 2465 | 54 | Horse bone | Slab burial | Miyamoto and Obata 2016 |
| Bayankhongor aimag, Bor Shorooonii Am Slab Burial 2 |  | 2545 | 28 | Horse tooth | Slab burial | Taylor 2018 |
| Uvurkhangai aimag, Khujirt sum, Shunkhlai Uul, AT-233, Grave 9 | OxA-36456 | 2826 | 30 | Human tooth | Slab burial | This study |
| Khentti amag, Binder sum, Bor Bulag, AT-707, Grave 2 | OxA-X-2737-55 | 2759 | 31 | Human tooth | Slab burial | This study |
| Ulaanbaatar aimag, Songinokhairkhan sum, Datsagt, AT-766, Grave 2 | OxA-36233 | 2436 | 26 | Human bone | Slab burial | This study |
| Uverkhangai  Khujirt sum  Maikhan Tolgoi  Gr 18 | NA | 2750 | 40 | Animal Bone | Slab Burial | Bemmann et al. 2015 |
| Uverkhangai  Khujirt sum  Maikhan Tolgoi  Gr 13 | NA | 2425 | 40 | Bone | Slab Burial | Bemmann et al. 2015 |
| Bulgan Aimag  Khutag Undur sum  Mukhdagiin am -1 | Beta 155036 | 2610 | 50 | Bone | Slab Burial | Turbat et al. 2003 |
| Bulgan Aimag  Khutag Undur sum  Bituugiin Tsagaan 1 | Beta 170633 | 2530 | 50 | Bone | Slab Burial | Turbat et al. 2003 |
| Bulgan Aimag  Khutag Undur sum  Mukhdagiin am -3 | Beta 170636 | 2510 | 40 | Bone | Slab Burial | Turbat et al. 2003 |
| Bulgan Aimag  Khutag Undur sum  Khariu uul-1 | Beta 170634 | 2450 | 40 | Tooth | Slab Burial | Turbat et al. 2003 |
| Bulgan Aimag  Khutag Undur sum  Kharuulin Gozgor-1 | Beta 170635 | 2370 | 40 | Bone | Slab Burial | Turbat et al. 2003 |
| Dundgovi aimag  Adaatsag sum  BGC 217 | OS-68948 | 3060 | 35 | Bone | Slab Burial | Johanneson 2016 |
| Bayan-Ulgii aimag, Tsengel sum, Khuiten Gol Delta 3 | Not reported | 3090 | 30 | Human tooth | Indeterminate mound | Fitzhugh 2012 |
| Khovd aimag, Mankhan sum, Khoit Tsenkher Cave, At-499, Stone mounds grave 11 | OxA-36229 | 3036 | 27 | Human bone | Indeterminate mound | This study |
| Khovd aimag, Mankhan sum, Khoit Tsenkher, AT-398, Grave 2 | OxA-362727 | 2828 | 28 | Human tooth | Indeterminate mound | This study |
| Bayan Ulgii aimag, Tsengel sum, Biluut 1D | Not reported | 2910 | 30 | Human tooth | Indeterminate mound | Fitzhugh et al 2012 |
| Bayan Ulgii aimag, Tsengel sum, Biluut 2-3 | Not reported | 2940 | 30 | Human bone | Indeterminate mound | Fitzhugh et al 2011 |
|  |  |  |  |  |  |  |
| Bayan Ulgii aimag, Tsengel sum, Biluut 2-4 | Not reported | 3030 | 30 | Human bone | Indeterminate mound | Fitzhugh et al 2011 |
| Sukhbaatar aimag, Delgerkh sum  CKU Burial 130 | AA100876 | 3023 | 41 | Bone | Indeterminate mound | Amartuvshin *et al*. 2015 |
| Dundgovi aimag  Adaatsag sum  Baga Mongol 07.19b | OS-68270 | 2440 | 25 | Bone | Indeterminate mound | Nelson et al. 2009 |
