# Supplementary material for: Radiocarbon dating and cultural dynamics across Mongolia’s early pastoral transition
Source: PLoS One. 2019 Nov 6;14(11):e0224241. doi: 10.1371/journal.pone.0224241 (PMC6834239; doi:10.1371/journal.pone.0224241)
Supplement: S2 Appendix — (DOCX) [file pone.0224241.s002.docx]

**S2 Appendix.** Oxcal code used in the analysis.

**Burial position – final model with trapezoid prior**

Plot()

{

Outlier_Model("RScaled",T(5),U(0,4),"r");

Sequence()

{

Boundary("Flexed Start")

{

Transition("T");

Start("S");

End("E");

};

Phase("Flexed")

{

R_Date("Bayan-Ulgii Biluut 2-4 human bone", 3030,30)

{

latitude = 48.65;

longitude = 88.31666667;

color = "blue";

};

R_Date("Bayan Ulgii aimag, Khuurai Gobi 1 bone", 4180,100)

{

latitude = 49.335333;

longitude = 88.712567;

color = "blue";

};

R_Date("Bayankhongor aimag, Shatar Chuluu Grave 5 (AT-25) human bone", 4410,31)

{

latitude = 46.30561667;

longitude = 100.7613889;

color = "blue";

};

R_Date("Bayankhongor aimag, Shatar Chuluu Grave 2 (AT-26) human tooth", 4415,31)

{

latitude = 46.30561667;

longitude = 100.7613889;

color = "blue";

};

R_Combine("Khovd aimag, Yagshiin Khuduu 1")

{

R_Date("Khovd aimag, Yagshiin Khuduu 1 human tooth (AT-590B)", 3983,17)

{

latitude = 46.12105;

longitude = 91.571833;

color = "blue";

Outlier(0.05);

};

R_Date("Khovd aimag, Yagshiin Khuduu 1 bone", 3790,120)

{

latitude = 46.12105;

longitude = 91.571833;

color = "blue";

Outlier(0.05);

};

};

R_Date("Khovd aimag, Yagshiin Khuduu 2 human bone", 3880,100)

{

latitude = 46.1253;

longitude = 91.5661;

color = "blue";

};

R_Combine("Khovd aimag, Yagshiin Khuduu 3")

{

R_Date("Kovalev and Erdenebaatar bone 1", 3770,60)

{

latitude = 46.121583;

longitude = 91.57195;

color = "blue";

Outlier(0.05);

};

R_Date("Kovalev and Erdenebaatar bone 2", 4000, 80)

{

Outlier(0.05);

};

R_Date("Kovalev and Erdenebaatar bone 3", 3800,70)

{

Outlier(0.05);

};

};

R_Date("Bayan-Ulgii aimag, Khundii Gobi Kurgan 1 burial 2 (AT-628) human bone", 4114,29)

{

latitude = 49.355;

longitude = 89.0286111;

color = "blue";

};

R_Combine("Khovd aimag, Kheviin Am 1")

{

R_Date("Kovalev and Erdenebaatar bone 1", 3800, 200)

{

latitude = 46.14475;

longitude = 91.49475;

color = "blue";

Outlier(0.05);

};

R_Date("Kovalev and Erdenebaatar bone 2", 3440, 120)

{

Outlier(0.05);

};

R_Date("Kovalev and Erdenebaatar bone 3", 3560,105)

{

Outlier(0.05);

};

};

R_Date("Khovd aimag, Kheviin Am 2 human bone", 3830,120)

{

latitude = 46.143367;

longitude = 91.4958;

color = "blue";

};

R_Date("Khovd aimag, Buural Khariin Ar bone", 4250,500)

{

latitude = 46.143367;

longitude = 91.4958;

color = "blue";

};

R_Date("Bayan-Ulgii aimag, Khuurai Gobi Kurgan 2 (AT-635)", 4034, 16)

{

latitude = 49.335333;

longitude = 88.712567;

color = "blue";

Outlier(0.05);

};

R_Date("Bayan-Ulgii aimag, Khul Uul bone", 3725,115)

{

latitude = 49.3591;

longitude = 88.713433;

color = "blue";

};

R_Date("Khovd aimag, Ulaan Goviin Uzuur 1",3310, 90)

{

latitude = 47.047374;

longitude = 91.839556;

color = "blue";

};

R_Date("Khovd aimag, Ulaan Goviin Uzuur 2 (AT-614) human tooth",3412, 32)

{

latitude = 47.047374;

longitude = 91.839556;

color = "blue";

Outlier(0.05);

};

R_Date("Khovd aimag, Khotuu Davaa 1 bone",3270, 60)

{

latitude = 47.047374;

longitude = 91.839556;

color = "blue";

};

R_Date("Khovd aimag, Artua bone",3480, 90)

{

latitude = 47.047374;

longitude = 91.839556;

color = "blue";

};

R_Date("Khovd aimag, Shar Gobi 3, Kurgan 1(AT-960) human tooth",3107, 31)

{

latitude = 47.059972;

longitude = 91.842502;

color = "blue";

};

R_Date("Khuvsgul aimag, Khogorgo 3 human tooth",3450, 50)

{

latitude = 51.412686;

longitude = 99.308339;

color = "blue";

};

Span("Span of Flexed dates");

Interval("Duration Flexed");

Sum("Flexed");

};

Boundary("Flexed End")

{

Transition("T");

Start("S");

End("E");

};

};

Sequence()

{

Boundary("Supine")

{

Transition("T");

Start("S");

End("E");

};

Phase()

{

R_Date("Uvurkhangai, Khujirt sum, Maikhan Tolgoi, Gr 13 bone ", 2425, 40)

{

latitude = 46.904218;

longitude = 102.768058;

color = "green";

};

R_Date("Bulgan Aimag, Khutag Undur sum, Mukhdagiin Am-1 bone ", 2610, 50)

{

latitude = 49.393151;

longitude = 102.700453;

color = "green";

};

R_Date("Bulgan Aimag, Khutag Undur sum, Bituugiin Tsagaan 1 bone ", 2530, 50)

{

latitude = 49.393151;

longitude = 102.700453;

color = "green";

};

R_Date("Bulgan Aimag, Khutag Undur sum, Mukhdagiin Am-3 bone ", 2510, 40)

{

latitude = 49.393151;

longitude = 102.700453;

color = "green";

};

R_Date("Bulgan Aimag, Khutag-Undur sum, Khariu Uul-1 tooth ", 2450, 40)

{

latitude = 49.393151;

longitude = 102.700453;

color = "green";

};

R_Date("Bulgan Aimag, Khutag-Undur sum, Kharuuliin Gozgor 1 bone ", 2370, 40)

{

latitude = 49.393151;

longitude = 102.700453;

color = "green";

};

R_Date("Dundgovi aimag, Adaatsag sum, BGC 217 bone ", 3060, 35)

{

latitude = 46.435446;

longitude = 105.772519;

color = "green";

};

R_Date("Uvurkhangai, Khujirt sum, Maikhan Tolgoi, Gr 52 bone ", 2970, 35)

{

latitude = 46.904218;

longitude = 102.768058;

color = "green";

};

R_Date("Bayan-Ulgii Biluut 2-3 human bone", 2940,30)

{

latitude = 48.65;

longitude = 88.31666667;

color = "green";

};

R_Date("Bayan-Ulgii Khuiten Gol Delta 2 human tooth", 2800,30)

{

latitude = 48.65567778;

longitude = 88.35843333;

color = "green";

};

R_Date("Bayan-Ulgii East Bay 4 human tooth", 3080,30)

{

latitude = 48.6361667;

longitude = 88.39525;

color = "green";

};

R_Date("Khuvsgul aimag, mound 52 human bone", 2842, 42)

{

latitude = 49.7810174;

longitude = 99.7996813;

color = "green";

};

R_Date("Khuvsgul aimag, mound 54 human bone", 2831,31)

{

latitude = 49.7810174;

longitude = 99.7996813;

color = "green";

};

R_Date("Bayankhongor aimag, Bor Ovoo HG-12 human bone", 3054,21)

{

latitude = 45.200119;

longitude = 100.765212;

color = "green";

};

R_Date("Khovd aimag, Berkh Mountain Khirigsuur 3 (AT-905) human tooth", 2989, 28)

{

latitude = 47.059972;

longitude = 91.842502;

color = "green";

};

R_Date("Khuvsgul aimag, Ulaan Tolgoi (AT-617) human tooth", 2921, 28)

{

latitude = 50.437764;

longitude = 100.125257;

color = "green";

};

R_Date("Dundgovi aimag, BGC 08-21 human bone", 3050, 30)

{

latitude = 46.203946;

longitude = 106.028742;

color = "green";

};

R_Date("Bulgan aimag, Egiin Gol, EGS 007 bone", 2610, 50)

{

latitude = 49.393374;

longitude = 102.699852;

color = "green";

};

R_Date("Bulgan aimag, Egiin Gol, EGS 361 bone", 2530, 50)

{

latitude = 49.393374;

longitude = 102.699852;

color = "green";

};

R_Date("Bulgan aimag, Egiin Gol, EGS 406 tooth", 2450, 40)

{

latitude = 49.393374;

longitude = 102.699852;

color = "green";

};

R_Date("Bulgan aimag, Egiin Gol, EGS 153 bone", 2400, 40)

{

latitude = 49.393374;

longitude = 102.699852;

color = "green";

};

R_Date("Bulgan aimag, Egiin Gol, EGS 450 bone", 2370, 40)

{

latitude = 49.393374;

longitude = 102.699852;

color = "green";

};

R_Date("Uvurkhangai aimag, Khujirt sum, Duruvljin Bulsh 2 human bone", 2855, 30)

{

latitude = 46.9593356;

longitude = 102.4401523;

color = "green";

};

R_Date("Uvurkhangai aimag, Khujirt sum, Duruvljin Bulsh 13 bone", 2430, 35)

{

latitude = 46.9593356;

longitude = 102.4401523;

color = "green";

};

R_Date("Uverkhangai aimag, Khujirt sum, Duruvljin Bulsh 10 human bone", 2230, 30)

{

latitude = 46.9593356;

longitude = 102.4401523;

color = "green";

};

R_Date("Daram, No. 4, M4 burial-A human bone", 2487, 50)

{

latitude = 47.173672;

longitude = 109.201748;

color = "green";

};

R_Date("Daram, D038-M2 human bone", 2455, 66)

{

latitude = 47.173672;

longitude = 109.201748;

color = "green";

};

R_Date("Orog Nuur bone", 2866, 55)

{

latitude = 45.200119;

longitude = 100.765212;

color = "green";

};

R_Date("Shunkhlai Uul, Grave 9 (AT-233) human tooth", 2826, 30)

{

latitude = 46.91096289;

longitude = 102.7708333;

color = "green";

};

R_Date("Bor Bulag, Grave 2 (AT-707) human tooth", 2759, 31)

{

latitude = 48.622122;

longitude = 110.620651;

color = "green";

};

R_Date("Dartsagt, Grave 2 (AT-766) human bone", 2436, 26)

{

latitude = 47.91111111;

longitude = 106.7486111;

color = "green";

};

R_Date("Baga Gazaryn Chuluu BGC, EX 07.07 human bone", 2340, 30)

{

latitude = 46.203946;

longitude = 106.082742;

color = "green";

};

R_Date("Baga Gazaryn Chuluu BGC, EX 07.01 human bone", 2745, 30)

{

latitude = 46.203946;

longitude = 106.082742;

color = "green";

};

R_Date("Khuvsgul aimag, Mound 18 human bone", 3074, 49)

{

latitude = 49.7810174;

longitude = 99.7996813;

color = "green";

};

R_Date("Khuvsgul aimag, Mound 58 human bone", 3056, 46)

{

latitude = 49.7810174;

longitude = 99.7996813;

color = "green";

};

R_Date("Khuvsgul aimag, Mound 25 human bone", 3052, 50)

{

latitude = 49.7810174;

longitude = 99.7996813;

color = "green";

};

R_Date("Khuvsgul aimag, Mound 23 human bone", 3052, 52)

{

latitude = 49.7810174;

longitude = 99.7996813;

color = "green";

};

R_Date("Khuvsgul aimag, Mound 3 human bone", 3044, 50)

{

latitude = 49.7810174;

longitude = 99.7996813;

color = "green";

};

R_Date("Khuvsgul aimag, Mound 10 human bone", 2992, 48)

{

latitude = 49.7810174;

longitude = 99.7996813;

color = "green";

};

R_Date("Khuvsgul aimag, Mound 9 human bone", 2991, 48)

{

latitude = 49.7810174;

longitude = 99.7996813;

color = "green";

};

R_Date("Khuvsgul aimag, Mound 55 human bone", 2990, 38)

{

latitude = 49.7810174;

longitude = 99.7996813;

color = "green";

};

R_Date("Khuvsgul aimag, Mound 13 human bone", 2989, 48)

{

latitude = 49.7810174;

longitude = 99.7996813;

color = "green";

};

R_Date("Khuvsgul aimag, Mound 2 human bone", 2910, 52)

{

latitude = 49.7810174;

longitude = 99.7996813;

color = "green";

};

R_Date("Khuvsgul aimag, Mound 7 human bone", 2897, 55)

{

latitude = 49.7810174;

longitude = 99.7996813;

color = "green";

};

R_Date("Khuvsgul aimag, Mound 8 human bone", 2872, 48)

{

latitude = 49.7810174;

longitude = 99.7996813;

color = "green";

};

R_Date("Khuvsgul aimag, Mound 6 human bone", 2857, 54)

{

latitude = 49.7810174;

longitude = 99.7996813;

color = "green";

};

R_Date("Khuvsgul aimag, Mound 14 human bone", 2849, 49)

{

latitude = 49.7810174;

longitude = 99.7996813;

color = "green";

};

R_Date("Khuvsgul aimag, Mound 27 human bone", 2835, 50)

{

latitude = 49.7810174;

longitude = 99.7996813;

color = "green";

};

R_Date("Bor Ovoo, HG-2 human bone", 3023, 23)

{

latitude = 45.200119;

longitude = 100.765212;

color = "green";

};

R_Date("Uliastai River I, AT-676, Kurgan 1, Burial 4 (main) human tooth", 2967, 31)

{

latitude = 45.85636;

longitude = 91.931779;

color = "green";

};

Span("Span of Supine dates");

Interval("Duration Supine");

Sum("Supine");

};

Boundary("Supine End")

{

Transition("T");

Start("S");

End("E");

};

};

Sequence()

{

Boundary("Prone Start")

{

Transition("T");

Start("S");

End("E");

};

Phase("Prone")

{

R_Date("Sukhbaatar aimag, Delgerkh sum, CKU burial 33 bone ", 3092, 52)

{

latitude = 45.80238;

longitude = 111.213634;

color = "red";

};

R_Date("Sukhbaatar aimag, Tuvshinshiree sum, DMS 657a bone ", 2930, 30)

{

latitude = 46.195161;

longitude = 111.859679;

color = "red";

};

R_Date("Sukhbaatar aimag, Tuvshinshiree sum, DMS 657c tooth ", 2880, 25)

{

latitude = 46.195161;

longitude = 111.859679;

color = "red";

};

R_Date("Sukhbaatar aimag, Delgerkh sum, CKU Burial 2-2004 ", 3230, 40)

{

latitude = 45.80238;

longitude = 111.213634;

color = "red";

};

R_Date("Sukhbaatar aimag, Delgerkh sum, CKU Burial 4-2004 ", 3170, 40)

{

latitude = 45.80238;

longitude = 111.213634;

color = "red";

};

R_Date("Sukhbaatar aimag, Delgerkh sum, CKU Burial 31 bone ", 3100, 55)

{

latitude = 45.80238;

longitude = 111.213634;

color = "red";

};

R_Date("Sukhbaatar aimag, Delgerkh sum, CKU Burial 41 bone ", 3057, 52)

{

latitude = 45.80238;

longitude = 111.213634;

color = "red";

};

R_Date("Dundgovi aimag, Adaatsag sum, Baga Mongol 07.23 bone ", 2990, 40)

{

latitude = 46.435446;

longitude = 105.772519;

color = "red";

};

R_Date("Sukhbaatar aimag, Delgerkh sum, CKU Burial 211 bone ", 3065, 41)

{

latitude = 45.80238;

longitude = 111.213634;

color = "red";

};

R_Date("Uvs aimag, Zuunkhangai SKT-B-1 human tooth", 3142, 30)

{

latitude = 49.41605556;

longitude = 95.61238889;

color = "red";

};

R_Date("Bayankhongor aimag, Zamyn Buts secondary burial bone",2980,110)

{

latitude = 44.545522;

longitude = 100.830588;

color = "red";

};

R_Date("Bayankhongor aimag, Baruun Gyalaat 2 bone",2900,50)

{

latitude = 44.545522;

longitude = 100.830588;

color = "red";

};

R_Date("Uvurkhangai aimag, Tevsh Uul No.1 Shape Burial 1 human bone",2706,20)

{

latitude = 44.738933;

longitude = 102.212205;

color = "red";

};

R_Date("Uvurkhangai aimag, Tevsh Uul No.3 Shape Burial 1 human bone",3050,20)

{

latitude = 44.738933;

longitude = 102.212205;

color = "red";

};

R_Date("Uvurkhangai aimag, Tevsh Uul No.4 Shape Burial 5 human bone",2912,20)

{

latitude = 44.738933;

longitude = 102.212205;

color = "red";

};

R_Date("Uvurkhangai aimag, Khujirt Shorgooljin Bulsh 3 human bone",3343,38)

{

latitude = 46.9593356;

longitude = 102.4401523;

color = "red";

};

R_Date("Sukhbaatar aimag, Dornod Mongol Ulaanzuukh Burial 6 human bone", 3127, 29)

{

latitude = 46.65133333;

longitude = 111.8616667;

color = "red";

};

R_Date("Sukhbaatar aimag, Dornod Mongol Ulaanzuukh Burial 2 human bone", 3115, 28)

{

latitude = 46.6515;

longitude = 111.8614444;

color = "red";

};

R_Date("Sukhbaatar aimag, Dornod Mongol Ulaanzuukh Burial A human bone", 3101, 30)

{

latitude = 46.65138889;

longitude = 111.8615556;

color = "red";

};

R_Date("Sukhbaatar aimag, Dornod Mongol Ulaanzuukh Burial B human bone", 3082, 31)

{

latitude = 46.65133333;

longitude = 111.8616111;

color = "red";

};

R_Date("Sukhbaatar aimag, Dornod Mongol Ulaanzuukh Burial C human bone", 3054, 29)

{

latitude = 46.65133333;

longitude = 111.8616944;

color = "red";

};

R_Date("Sukhbaatar aimag, Dornod Mongol Ulaanzuukh Burial D human bone", 3015, 28)

{

latitude = 46.65125;

longitude = 111.8615556;

color = "red";

};

R_Date("Sukhbaatar aimag, Dornod Mongol Ulaanzuukh Burial 3 human bone", 3006, 30)

{

latitude = 46.65144444;

longitude = 111.8615;

color = "red";

};

R_Date("Ulaanzuukh Grave 42 (AT-769) human tooth", 3215, 40)

{

latitude = 46.28406;

longitude = 111.768265;

color = "red";

};

R_Combine("Ulaanzuukh, Grave 1 (AT-824) human bone")

{

R_Date("Oxford 1", 3110, 31)

{

latitude = 46.28406;

longitude = 111.768265;

color = "red";

};

R_Date("Groningen 2", 3069, 16)

{

color = "red";

};

};

R_Date("Ulaanzuukh, Grave 1 (AT-823) human bone", 3028, 25)

{

latitude = 46.28406;

longitude = 111.768265;

color = "red";

};

R_Date("Ulaanzuukh, Grave 33 (AT-921) human bone", 3075, 27)

{

latitude = 46.28406;

longitude = 111.768265;

color = "red";

};

R_Date("Dundgovi aimag, BGC Baga Mongol EX 07.23 human bone", 2990, 40)

{

latitude = 46.28406;

longitude = 111.768265;

color = "red";

};

Span("Prone dates");

Interval("Duration Prone");

Sum("Prone");

};

Boundary("Prone End")

{

Transition("T");

Start("S");

End("E");

};

};

Sequence()

{

Boundary("Supine with knees bent Start")

{

Transition("T");

Start("S");

End("E");

};

Phase("Supine with knees bent")

{

R_Date("Dundgovi aimag, Adaatsag sum, Baga Mongol 07.19b bone ", 2440, 25)

{

latitude = 46.435446;

longitude = 105.772519;

color = "orange";

};

R_Date("Khovd aimag, Kheviin Am bone",2910, 90)

{

latitude = 46.14475;

longitude = 91.49475;

color = "orange";

};

R_Date("Khovd aimag, Uliastai River (lower terrace) I, Kurgan 4(AT-677) human tooth",2805, 16)

{

latitude = 45.85636;

longitude = 91.931779;

color = "orange";

};

R_Date("Khovd aimag, Uliastai Zastav II, Kurgan 2 (AT-674) human tooth",2824, 28)

{

latitude = 45.85636;

longitude = 91.931779;

color = "orange";

};

R_Combine("Khoit Tsenkher Cave, AT-499, Stone mounds grave 11")

{

R_Date("Oxford 1", 3036, 27)

{

latitude = 47.059972;

longitude = 91.842502;

color = "orange";

};

R_Date("Oxford 2", 2988, 29)

{

color = "orange";

};

};

R_Date("Khoit Tsenkher Cave, AT-398, Grave 2 human tooth", 2828, 28)

{

latitude = 47.059972;

longitude = 91.842502;

color = "orange";

};

Span("Span of Supine with knees bent dates");

Interval("Duration Supine with knees bent");

Sum("Supine with knees bent");

};

Boundary("Supine with knees bent End")

{

Transition("T");

Start("S");

End("E");

};

};

};

**Burial position – quality control-restricted model with trapezoid prior**

Plot()

{

Outlier_Model("RScaled",T(5),U(0,4),"r");

Sequence()

{

Boundary("Flexed Start")

{

Transition("T");

Start("S");

End("E");

};

Phase("Flexed")

{

R_Date("Bayankhongor aimag, Shatar Chuluu Grave 5 (AT-25) human bone", 4410,31)

{

latitude = 46.30561667;

longitude = 100.7613889;

color = "blue";

};

R_Date("Bayan-Ulgii aimag, Khundii Gobi Kurgan 1 burial 2 (AT-628) human bone", 4114,29)

{

latitude = 49.355;

longitude = 89.0286111;

color = "blue";

};

Span("Span of Flexed dates");

Interval("Duration Flexed");

Sum("Flexed");

};

Boundary("Flexed End")

{

Transition("T");

Start("S");

End("E");

};

};

Sequence()

{

Boundary("Supine start")

{

Transition("T");

Start("S");

End("E");

};

Phase()

{

R_Date("Dartsagt, Grave 2 (AT-766) human bone", 2436, 26)

{

latitude = 47.91111111;

longitude = 106.7486111;

color = "green";

};

Span("Span of Supine dates");

Interval("Duration Supine");

Sum("Supine");

};

Boundary("Supine End")

{

Transition("T");

Start("S");

End("E");

};

};

Sequence()

{

Boundary("Prone Start")

{

Transition("T");

Start("S");

End("E");

};

Phase("Prone")

{

R_Combine("Ulaanzuukh, Grave 1 (AT-824) human bone")

{

R_Date("Oxford 1", 3110, 31)

{

latitude = 46.28406;

longitude = 111.768265;

color = "red";

};

R_Date("Groningen 2", 3069, 16)

{

color = "red";

};

};

R_Date("Ulaanzuukh, Grave 1 (AT-823) human bone", 3028, 25)

{

latitude = 46.28406;

longitude = 111.768265;

color = "red";

};

R_Date("Ulaanzuukh, Grave 33 (AT-921) human bone", 3075, 27)

{

latitude = 46.28406;

longitude = 111.768265;

color = "red";

};

Span("Prone dates");

Interval("Duration Prone");

Sum("Prone");

};

Boundary("Prone End")

{

Transition("T");

Start("S");

End("E");

};

};

Sequence()

{

Boundary("Supine with knees bent Start")

{

Transition("T");

Start("S");

End("E");

};

Phase("Supine with knees bent")

{

R_Date("Khovd aimag, Uliastai River (lower terrace) I, Kurgan 4(AT-677) human tooth",2805, 16)

{

latitude = 45.85636;

longitude = 91.931779;

color = "orange";

};

R_Combine("Khoit Tsenkher Cave, AT-499, Stone mounds grave 11")

{

R_Date("Oxford 1", 3036, 27)

{

latitude = 47.059972;

longitude = 91.842502;

color = "orange";

};

R_Date("Oxford 2", 2988, 29)

{

color = "orange";

};

};

Span("Span of Supine with knees bent dates");

Interval("Duration Supine with knees bent");

Sum("Supine with knees bent");

};

Boundary("Supine with knees bent End")

{

Transition("T");

Start("S");

End("E");

};

};

};

**Burial traditions – final model with trapezoid prior**

Plot()

{

Outlier_Model("RScaled",T(5),U(0,4),"r");

Sequence()

{

Boundary("Afanasievo Start")

{

Transition("T");

Start("S");

End("E");

};

Phase("Afanasievo")

{

R_Date("Bayan Ulgii aimag, Khuurai Gobi 1 bone", 4180,100)

{

latitude = 49.335333;

longitude = 88.712567;

color = "black";

};

R_Date("Bayankhongor aimag, Shatar Chuluu Grave 5 (AT-25) human bone", 4410,31)

{

latitude = 46.30561667;

longitude = 100.7613889;

color = "black";

};

R_Date("Bayankhongor aimag, Shatar Chuluu Grave 2 (AT-26) human tooth", 4415,31)

{

latitude = 46.30561667;

longitude = 100.7613889;

color = "black";

};

Span("Span of Afanasievo dates");

Interval("Duration Afanasievo");

Sum("Afanasievo ");

};

Boundary("Afanasievo End")

{

Transition("T");

Start("S");

End("E");

};

};

Sequence()

{

Boundary("Chemurchek Start")

{

Transition("T");

Start("S");

End("E");

};

Phase("Chemurchek")

{

R_Combine("Khovd aimag, Yagshiin Khuduu 1")

{

R_Date("Khovd aimag, Yagshiin Khuduu 1 human tooth (AT-590B)", 3983,17)

{

latitude = 46.12105;

longitude = 91.571833;

color = "white";

Outlier(0.05);

};

R_Date("Khovd aimag, Yagshiin Khuduu 1 bone", 3790,120)

{

latitude = 46.12105;

longitude = 91.571833;

color = "white";

Outlier(0.05);

};

};

R_Date("Khovd aimag, Yagshiin Khuduu 2 human bone", 3880,100)

{

latitude = 46.1253;

longitude = 91.5661;

color = "white";

};

R_Combine("Khovd aimag, Yagshiin Khuduu 3")

{

R_Date("Kovalev and Erdenebaatar bone 1", 3770,60)

{

latitude = 46.121583;

longitude = 91.57195;

color = "white";

Outlier(0.05);

};

R_Date("Kovalev and Erdenebaatar bone 2", 4000, 80)

{

Outlier(0.05);

};

R_Date("Kovalev and Erdenebaatar bone 3", 3800,70)

{

Outlier(0.05);

};

};

R_Date("Bayan-Ulgii aimag, Khundii Gobi Kurgan 1 burial 2 (AT-628) human bone", 4114,29)

{

latitude = 49.355;

longitude = 89.0286111;

color = "white";

};

R_Combine("Khovd aimag, Kheviin Am 1")

{

R_Date("Kovalev and Erdenebaatar bone 1", 3800, 200)

{

latitude = 46.14475;

longitude = 91.49475;

color = "white";

};

R_Date("Kovalev and Erdenebaatar bone 2", 3440, 120)

{

Outlier(0.05);

};

R_Date("Kovalev and Erdenebaatar bone 3", 3560,105)

{

Outlier(0.05);

};

};

R_Date("Khovd aimag, Kheviin Am 2 human bone", 3830,120)

{

latitude = 46.143367;

longitude = 91.4958;

color = "black";

};

R_Date("Khovd aimag, Buural Khariin Ar bone", 4250,500)

{

latitude = 46.143367;

longitude = 91.4958;

color = "black";

};

Span("Span of Chemurchek dates");

Interval("Duration Chemurchek");

Sum("Chemurchek");

};

Boundary("Chemurchek End")

{

Transition("T");

Start("S");

End("E");

};

};

Sequence()

{

Boundary("Chemurchek/Afanasievo Start")

{

Transition("T");

Start("S");

End("E");

};

Phase("Chemurchek/Afanasievo ")

{

R_Date("Bayan-Ulgii aimag, Khuurai Gobi Kurgan 2 (AT-635)", 4034, 16)

{

latitude = 49.335333;

longitude = 88.712567;

color = "grey";

Outlier(0.05);

};

R_Date("Bayan-Ulgii aimag, Khundii Gobi Kurgan 1 (AT-628) human bone", 4114, 29)

{

latitude = 49.287633;

longitude = 88.876383;

color = "grey";

};

R_Date("Bayan-Ulgii aimag, Khul Uul bone", 3725,115)

{

latitude = 49.3591;

longitude = 88.713433;

color = "grey";

};

Span("Span of Chemurchek/Afanasievo dates");

Interval("Duration Chemurchek/Afanasievo ");

Sum("Chemurchek/Afanasievo");

};

Boundary("Chemurchek/Afanasievo End")

{

Transition("T");

Start("S");

End("E");

};

};

Sequence()

{

Boundary("Munkkhairkhan Start")

{

Transition("T");

Start("S");

End("E");

};

Phase("Munkkhairkhan")

{

R_Date("Khovd aimag, Ulaan Goviin Uzuur 1",3310, 90)

{

latitude = 47.047374;

longitude = 91.839556;

color = "purple";

};

R_Date("Khovd aimag, Ulaan Goviin Uzuur 2 (AT-614) human tooth",3412, 32)

{

latitude = 47.047374;

longitude = 91.839556;

color = "purple";

Outlier(0.05);

};

R_Date("Khovd aimag, Khotuu Davaa 1 bone",3270, 60)

{

latitude = 47.047374;

longitude = 91.839556;

color = "purple";

};

R_Date("Khovd aimag, Artua bone",3480, 90)

{

latitude = 47.047374;

longitude = 91.839556;

color = "purple";

};

R_Date("Khovd aimag, Shar Gobi 3, Kurgan 1(AT-960) human tooth",3107, 31)

{

latitude = 47.059972;

longitude = 91.842502;

color = "purple";

};

R_Date("Khuvsgul aimag, Khogorgo 3 human tooth",3450, 50)

{

latitude = 51.412686;

longitude = 99.308339;

color = "purple";

};

Span("Span of Munkkhairkhan dates");

Interval("Duration Munkkhairkhan");

Sum("Munkkhairkhan");

};

Boundary("Munkkhairkhan End")

{

Transition("T");

Start("S");

End("E");

};

};

Sequence()

{

Boundary("Khirigsuur mound Start")

{

Transition("T");

Start("S");

End("E");

};

Phase()

{

R_Date("Bayan-Ulgii Khuiten Gol Delta 2 human tooth", 2800,30)

{

latitude = 48.65567778;

longitude = 88.35843333;

color = "blue";

};

R_Date("Bayan-Ulgii KGD2 human tooth", 2800,30)

{

longitude = 88.35843333333334;

latitude = 48.6556777777777;

color = "blue";

};

R_Date("Khuvsgul aimag, mound 52 human bone", 2842, 42)

{

latitude = 49.7810174;

longitude = 99.7996813;

color = "blue";

};

R_Date("Khuvsgul aimag, mound 54 human bone", 2831,31)

{

latitude = 49.7810174;

longitude = 99.7996813;

color = "blue";

};

R_Date("Bayankhongor aimag, Bor Ovoo HG-12 human bone", 3054,21)

{

latitude = 45.200119;

longitude = 100.765212;

color = "blue";

};

R_Date("Khovd aimag, Berkh Mountain Khirigsuur 3 (AT-905) human tooth", 2989, 28)

{

latitude = 47.059972;

longitude = 91.842502;

color = "blue";

};

R_Date("Khuvsgul aimag, Ulaan Tolgoi (AT-617) human tooth", 2921, 28)

{

latitude = 50.437764;

longitude = 100.125257;

color = "blue";

};

R_Date("Dundgovi aimag, BGC 08-21 human bone", 3050, 30)

{

latitude = 46.203946;

longitude = 106.028742;

color = "blue";

};

Span("Span of Khirigsuur mound dates");

Interval("Duration Khirigsuur mound");

Sum("Khirigsuur mound");

};

Boundary("Khirigsuur mound End")

{

Transition("T");

Start("S");

End("E");

};

};

Sequence()

{

Boundary("D Shape Start")

{

Transition("T");

Start("S");

End("E");

};

Phase("D Shape")

{

R_Date("Bayankhongor aimag, Zamyn Buts secondary burial bone",2980,110)

{

latitude = 44.545522;

longitude = 100.830588;

color = "magenta";

};

R_Date("Bayankhongor aimag, Baruun Gyalaat 2 bone",2900,50)

{

latitude = 44.545522;

longitude = 100.830588;

color = "magenta";

};

Span("Span of D Shape dates");

Interval("Duration D Shape");

Sum("D Shape");

};

Boundary("D Shape End")

{

Transition("T");

Start("S");

End("E");

};

};

Sequence()

{

Boundary("Baitag Start")

{

Transition("T");

Start("S");

End("E");

};

Phase("Baitag")

{

R_Date("Khovd aimag, Kheviin Am bone",2910, 90)

{

latitude = 46.14475;

longitude = 91.49475;

color = "pink";

};

R_Date("Khovd aimag, Uliastai River (lower terrace) I, Kurgan 4(AT-677)",2805, 16)

{

latitude = 45.85636;

longitude = 91.931779;

color = "pink";

};

R_Date("Khovd aimag, Uliastai Zastav II, Kurgan 2 (AT-674)",2824, 28)

{

latitude = 45.85636;

longitude = 91.931779;

color = "pink";

};

Span("Span of Baitag dates");

Interval("Duration Baitag ");

Sum("Baitag");

};

Boundary("Baitag End")

{

Transition("T");

Start("S");

End("E");

};

};

Sequence()

{

Boundary("Shape Burial Start")

{

Transition("T");

Start("S");

End("E");

};

Phase("Shape Burial")

{

R_Date("Bayankhongor Ulziit Shape Burial horse tooth", 2670, 30)

{

latitude = 46.061791;

longitude = 100.829492;

color = "orange";

};

R_Date("Uvurkhangai aimag, Tevsh Uul No.1 Shape Burial 1 human bone",2706,20)

{

latitude = 44.738933;

longitude = 102.212205;

color = "orange";

};

R_Date("Uvurkhangai aimag, Tevsh Uul No.3 Shape Burial 1 human bone",3050,20)

{

latitude = 44.738933;

longitude = 102.212205;

color = "orange";

};

R_Date("Uvurkhangai aimag, Tevsh Uul No.4 Shape Burial 5 human bone",2912,20)

{

latitude = 44.738933;

longitude = 102.212205;

color = "orange";

};

R_Date("Uvurkhangai aimag, Khujirt Shorgooljin Bulsh 3 human bone",3343,38)

{

latitude = 46.9593356;

longitude = 102.4401523;

color = "orange";

};

Span("Span of Shape Burial dates");

Interval("Duration Shape Burial");

Sum("Shape Burial ");

};

Boundary("Shape Burial End")

{

Transition("T");

Start("S");

End("E");

};

};

Sequence()

{

Boundary("Ulaanzuukh Start")

{

Transition("T");

Start("S");

End("E");

};

Phase("Ulaanzuukh")

{

R_Date("Sukhbaatar aimag, Delgerekh sum, CKU burial 33 bone ", 3092, 52)

{

latitude = 45.80238;

longitude = 111.213634;

color = "red";

};

R_Date("Sukhbaatar aimag, Tuvshinshiree sum, DMS 657a bone ", 2930, 30)

{

latitude = 46.195161;

longitude = 111.859679;

color = "red";

};

R_Date("Sukhbaatar aimag, Tuvshinshiree sum, DMS 657c tooth ", 2880, 25)

{

latitude = 46.195161;

longitude = 111.859679;

color = "red";

};

R_Date("Sukhbaatar aimag, Delgerekh sum, CKU Burial 2-2004 ", 3230, 40)

{

latitude = 45.80238;

longitude = 111.213634;

color = "red";

};

R_Date("Sukhbaatar aimag, Delgerekh sum, CKU Burial 4-2004 ", 3170, 40)

{

latitude = 45.80238;

longitude = 111.213634;

color = "red";

};

R_Date("Sukhbaatar aimag, Delgerekh sum, CKU Burial 31 bone ", 3100, 55)

{

latitude = 45.80238;

longitude = 111.213634;

color = "red";

};

R_Date("Sukhbaatar aimag, Delgerekh sum, CKU Burial 41 bone ", 3057, 52)

{

latitude = 45.80238;

longitude = 111.213634;

color = "red";

};

R_Date("Dundgovi aimag, Adaatsag sum, Baga Mongol 07.23 bone ", 2990, 40)

{

latitude = 46.435446;

longitude = 105.772519;

color = "red";

};

R_Date("Sukhbaatar aimag, Delgerekh sum, CKU Burial 211 bone ", 3065, 41)

{

latitude = 45.80238;

longitude = 111.213634;

color = "red";

};

R_Date("Sukhbaatar aimag, Dornod Mongol Ulaanzuukh Burial 6 human bone", 3127, 29)

{

latitude = 46.65133333;

longitude = 111.8616667;

color = "red";

};

R_Date("Sukhbaatar aimag, Dornod Mongol Ulaanzuukh Burial 2 human bone", 3115, 28)

{

latitude = 46.6515;

longitude = 111.8614444;

color = "red";

};

R_Date("Sukhbaatar aimag, Dornod Mongol Ulaanzuukh Burial A human bone", 3101, 30)

{

latitude = 46.65138889;

longitude = 111.8615556;

color = "red";

};

R_Date("Sukhbaatar aimag, Dornod Mongol Ulaanzuukh Burial B human bone", 3082, 31)

{

latitude = 46.65133333;

longitude = 111.8616111;

color = "red";

};

R_Date("Sukhbaatar aimag, Dornod Mongol Ulaanzuukh Burial C human bone", 3054, 29)

{

latitude = 46.65133333;

longitude = 111.8616944;

color = "red";

};

R_Date("Sukhbaatar aimag, Dornod Mongol Ulaanzuukh Burial D human bone", 3015, 28)

{

latitude = 46.65125;

longitude = 111.8615556;

color = "red";

};

R_Date("Sukhbaatar aimag, Dornod Mongol Ulaanzuukh Burial 3 human bone", 3006, 30)

{

latitude = 46.65144444;

longitude = 111.8615;

color = "red";

};

R_Date("Ulaanzuukh Grave 42 (AT-769) human tooth", 3215, 40)

{

latitude = 46.28406;

longitude = 111.768265;

color = "red";

};

R_Combine("Ulaanzuukh, Grave 1 (AT-824) human bone")

{

R_Date("Oxford 1", 3110, 31)

{

latitude = 46.28406;

longitude = 111.768265;

color = "red";

};

R_Date("Groningen 2", 3069, 16)

{

color = "red";

};

};

R_Date("Ulaanzuukh, Grave 1 (AT-823) human bone", 3028, 25)

{

latitude = 46.28406;

longitude = 111.768265;

color = "red";

};

R_Date("Ulaanzuukh, Grave 33 (AT-921) human bone", 3075, 27)

{

latitude = 46.28406;

longitude = 111.768265;

color = "red";

};

R_Date("Dundgovi aimag, Baga Gazaryn Chuluu, Baga Mongol EX 07.23 human bone", 2990, 40)

{

latitude = 46.28406;

longitude = 111.768265;

color = "red";

};

Span("Span of Ulaanzuukh dates");

Interval("Duration Ulaanzuukh");

Sum("Ulaanzuukh");

};

Boundary("Ulaanzuukh End")

{

Transition("T");

Start("S");

End("E");

};

};

Sequence()

{

Boundary("Slab Burial Start")

{

Transition("T");

Start("S");

End("E");

};

Phase("Slab Burial")

{

R_Date("Uvurkhangai, Khujirt sum, Maikhan Tolgoi, Gr 13 bone ", 2425, 40)

{

latitude = 46.904218;

longitude = 102.768058;

color = "white";

};

R_Date("Bulgan Aimag, Khutag Undur sum, Mukhdagiin Am-1 bone ", 2610, 50)

{

latitude = 49.393151;

longitude = 102.700453;

color = "white";

};

R_Date("Bulgan Aimag, Khutag Undur sum, Bituugiin Tsagaan 1 bone ", 2530, 50)

{

latitude = 49.393151;

longitude = 102.700453;

color = "white";

};

R_Date("Bulgan Aimag, Khutag Undur sum, Mukhdagiin Am-3 bone ", 2510, 40)

{

latitude = 49.393151;

longitude = 102.700453;

color = "white";

};

R_Date("Bulgan Aimag, Khutag-Undur sum, Khariu Uul-1 tooth ", 2450, 40)

{

latitude = 49.393151;

longitude = 102.700453;

color = "white";

};

R_Date("Bulgan Aimag, Khutag-Undur sum, Kharuuliin Gozgor 1 bone ", 2370, 40)

{

latitude = 49.393151;

longitude = 102.700453;

color = "white";

};

R_Date("Dundgovi aimag, Adaatsag sum, BGC 217 bone ", 3060, 35)

{

latitude = 46.435446;

longitude = 105.772519;

color = "white";

};

R_Date("Bulgan aimag, Egiin Gol, EGS 007 bone", 2610, 50)

{

latitude = 49.393374;

longitude = 102.699852;

color = "white";

};

R_Date("Bulgan aimag, Egiin Gol, EGS 361 bone", 2530, 50)

{

latitude = 49.393374;

longitude = 102.699852;

color = "white";

};

R_Date("Bulgan aimag, Egiin Gol, EGS 406 tooth", 2450, 40)

{

latitude = 49.393374;

longitude = 102.699852;

color = "white";

};

R_Date("Bulgan aimag, Egiin Gol, EGS 153 bone", 2400, 40)

{

latitude = 49.393374;

longitude = 102.699852;

color = "white";

};

R_Date("Bulgan aimag, Egiin Gol, EGS 450 bone", 2370, 40)

{

latitude = 49.393374;

longitude = 102.699852;

color = "white";

};

R_Date("Uvurkhangai aimag, Khujirt sum, Duruvljin Bulsh 2 human bone", 2855, 30)

{

latitude = 46.9593356;

longitude = 102.4401523;

color = "white";

};

R_Date("Uvurkhangai aimag, Khujirt sum, Duruvljin Bulsh 13 bone", 2430, 35)

{

latitude = 46.9593356;

longitude = 102.4401523;

color = "white";

};

R_Date("Uvurkhangai aimag, Khujirt sum, Duruvljin Bulsh 10 human bone", 2230, 30)

{

latitude = 46.9593356;

longitude = 102.4401523;

color = "white";

};

R_Date("Daram, No. 4, M4 burial-A human bone", 2487, 50)

{

latitude = 47.173672;

longitude = 109.201748;

color = "white";

};

R_Date("Daram, D038-M2 human bone", 2455, 66)

{

latitude = 47.173672;

longitude = 109.201748;

color = "white";

};

R_Date("Orog Nuur bone", 2866, 55)

{

latitude = 45.200119;

longitude = 100.765212;

color = "white";

};

R_Date("Shunkhlai Uul, Grave 9 (AT-233) human tooth", 2826, 30)

{

latitude = 46.91096289;

longitude = 102.7708333;

color = "white";

};

R_Date("Bor Bulag, Grave 2 (AT-707) human tooth", 2759, 31)

{

latitude = 48.622122;

longitude = 110.620651;

color = "white";

};

R_Date("Dartsagt, Grave 2 (AT-766) human bone", 2436, 26)

{

latitude = 47.91111111;

longitude = 106.7486111;

color = "white";

};

R_Date("Baga Gazaryn Chuluu BGC, EX 07.07 human bone", 2340, 30)

{

latitude = 46.203946;

longitude = 106.082742;

color = "white";

};

R_Date("Baga Gazaryn Chuluu BGC, EX 07.01 human bone", 2745, 30)

{

latitude = 46.203946;

longitude = 106.082742;

color = "white";

};

Span("Span of Slab Burial dates");

Interval("Duration Slab Burial");

Sum("Slab Burial");

};

Boundary("Slab Burial End")

{

Transition("T");

Start("S");

End("E");

};

};

Sequence()

{

Boundary("Slab Burial animal Start")

{

Transition("T");

Start("S");

End("E");

};

Phase("Slab Burial animal")

{

R_Date("Uvurkhangai, Khujirt sum, Maikhan Tolgoi, Gr 18 animal bone ", 2750, 40)

{

latitude = 46.904218;

longitude = 102.768058;

color = "white";

};

R_Date("Bayankhongor Bor Shoroonii Am Slab Burial 2 horse tooth", 2545, 28)

{

latitude = 46.40681667;

longitude = 100.8036111;

color = "white";

};

R_Date("Arkhangai Jargalantyn Am Slab Burial horse tooth", 2670, 30)

{

latitude = 48.172222;

longitude = 101.092917;

color = "white";

};

R_Date("Daram, D048 cattle bone", 2395, 50)

{

latitude = 47.173672;

longitude = 109.201748;

color = "white";

};

R_Date("Daram, D21 M1 cattle bone", 2278, 42)

{

latitude = 47.173672;

longitude = 109.201748;

color = "white";

};

R_Date("Daram, D21 M41 cattle bone", 1924, 41)

{

latitude = 47.173672;

longitude = 109.201748;

color = "white";

};

R_Date("Daram, D12 M1 horse bone", 2368, 37)

{

latitude = 47.173672;

longitude = 109.201748;

color = "white";

};

R_Date("Daram, D42 M1 horse bone", 2350, 46)

{

latitude = 47.173672;

longitude = 109.201748;

color = "white";

};

R_Date("Daram, D47 M1 horse bone", 2465, 54)

{

latitude = 47.173672;

longitude = 109.201748;

color = "white";

};

Span("Span of Slab Burial animal dates");

Interval("Duration Slab Burial animal");

Sum("Slab Burial animal");

};

Boundary("Slab Burial animal End")

{

Transition("T");

Start("S");

End("E");

};

};

Sequence()

{

Boundary("Sagsai Start")

{

Transition("T");

Start("S");

End("E");

};

Phase("Sagsai")

{

R_Date("Uvurkhangai, Khujirt sum, Maikhan Tolgoi, Gr 52 bone", 2970, 35)

{

latitude = 46.904218;

longitude = 102.768058;

color = "black";

};

R_Date("Bayan-Ulgii East Bay 4 human tooth", 3080,30)

{

latitude = 48.6361667;

longitude = 88.39525;

color = "black";

};

R_Date("Khuvsgul aimag, Mound 18 human bone", 3074, 49)

{

latitude = 49.7810174;

longitude = 99.7996813;

color = "black";

};

R_Date("Khuvsgul aimag, Mound 58 human bone", 3056, 46)

{

latitude = 49.7810174;

longitude = 99.7996813;

color = "black";

};

R_Date("Khuvsgul aimag, Mound 25 human bone", 3052, 50)

{

latitude = 49.7810174;

longitude = 99.7996813;

color = "black";

};

R_Date("Khuvsgul aimag, Mound 23 human bone", 3052, 52)

{

latitude = 49.7810174;

longitude = 99.7996813;

color = "black";

};

R_Date("Khuvsgul aimag, Mound 3 human bone", 3044, 50)

{

latitude = 49.7810174;

longitude = 99.7996813;

color = "black";

};

R_Date("Khuvsgul aimag, Mound 10 human bone", 2992, 48)

{

latitude = 49.7810174;

longitude = 99.7996813;

color = "black";

};

R_Date("Khuvsgul aimag, Mound 9 human bone", 2991, 48)

{

latitude = 49.7810174;

longitude = 99.7996813;

color = "black";

};

R_Date("Khuvsgul aimag, Mound 55 human bone", 2990, 38)

{

latitude = 49.7810174;

longitude = 99.7996813;

color = "black";

};

R_Date("Khuvsgul aimag, Mound 13 human bone", 2989, 48)

{

latitude = 49.7810174;

longitude = 99.7996813;

color = "black";

};

R_Date("Khuvsgul aimag, Mound 2 human bone", 2910, 52)

{

latitude = 49.7810174;

longitude = 99.7996813;

color = "black";

};

R_Date("Khuvsgul aimag, Mound 7 human bone", 2897, 55)

{

latitude = 49.7810174;

longitude = 99.7996813;

color = "black";

};

R_Date("Khuvsgul aimag, Mound 8 human bone", 2872, 48)

{

latitude = 49.7810174;

longitude = 99.7996813;

color = "black";

};

R_Date("Khuvsgul aimag, Mound 6 human bone", 2857, 54)

{

latitude = 49.7810174;

longitude = 99.7996813;

color = "black";

};

R_Date("Khuvsgul aimag, Mound 14 human bone", 2849, 49)

{

latitude = 49.7810174;

longitude = 99.7996813;

color = "black";

};

R_Date("Khuvsgul aimag, Mound 27 human bone", 2835, 50)

{

latitude = 49.7810174;

longitude = 99.7996813;

color = "black";

};

R_Date("Bor Ovoo, HG-2 human bone", 3023, 23)

{

latitude = 45.200119;

longitude = 100.765212;

color = "black";

};

R_Date("Uliastai River I, AT-676, Kurgan 1, Burial 4 (main) human tooth", 2967, 31)

{

latitude = 45.85636;

longitude = 91.931779;

color = "black";

};

Span("Span of Sagsai dates");

Interval("Duration Sagsai");

Sum("Sagsai ");

};

Boundary("Sagsai End")

{

Transition("T");

Start("S");

End("E");

};

};

Sequence()

{

Boundary("Rando Start")

{

Transition("T");

Start("S");

End("E");

};

Phase("Rando")

{

R_Date("Sukhbaatar aimag, Delgerekh sum, CKU Burial 130 bone", 3023,41)

{

latitude = 45.80238;

longitude = 111.213634;

color = "yellow";

};

R_Date("Dundgovi aimag, Adaatsag sum, Baga Mongol 07.19b bone", 2440, 25)

{

latitude = 46.435446;

longitude = 105.772519;

color = "yellow";

};

R_Combine("Khoit Tsenkher Cave, AT-499, Stone mounds grave 11")

{

R_Date("Oxford 1", 3036, 27)

{

latitude = 47.059972;

longitude = 91.842502;

color = "yellow";

};

R_Date("Oxford 2", 2988, 29)

{

color = "yellow";

};

};

R_Date("Khoit Tsenkher Cave, AT-398, Grave 2", 2828, 28)

{

latitude = 47.059972;

longitude = 91.842502;

color = "yellow";

};

R_Date("Uvs aimag, Zuunkhangai SKT-B-1 human tooth", 3142, 30)

{

latitude = 49.41605556;

longitude = 95.61238889;

color = "yellow";

};

R_Date("Bayan-Ulgii Biluut 2-4 human bone", 3030,30)

{

latitude = 48.65;

longitude = 88.31666667;

color = "yellow";

};

R_Date("Bayan-Ulgii Biluut 2-3 human bone", 2940,30)

{

latitude = 48.65;

longitude = 88.31666667;

color = "yellow";

};

R_Date("Bayan-Ulgii Biluut 1D human tooth", 2910,30)

{

latitude = 48.6833333;

longitude = 88.40388889;

color = "yellow";

};

R_Date("Bayan-Ulgii Khuiten Gol Delta 3 human tooth", 3090,30)

{

latitude = 48.6307;

longitude = 88.3585667;

color = "yellow";

};

Span("Span of Rando dates");

Interval("Duration Rando");

Sum("Rando");

};

Boundary("Rando End")

{

Transition("T");

Start("S");

End("E");

};

};

Sequence()

{

Boundary("DS Start")

{

Transition("T");

Start("S");

End("E");

};

Phase("DS")

{

R_Date("Arkhangai Tsatsyn Ereg DS38 F27 tooth", 2840,25)

{

latitude = 47.981369;

longitude = 101.546887;

color = "green";

};

R_Date("Arkhangai Tsatsyn Ereg DS38 F95 tooth", 2840,25)

{

latitude = 47.981369;

longitude = 101.546887;

color = "green";

};

R_Date("Arkhangai Tsatsyn Ereg DS38 F1 Structure 1 tooth", 2860,25)

{

latitude = 47.981369;

longitude = 101.546887;

color = "green";

};

R_Date("Khuvsgul TK DS1 F2 horse tooth", 3000,40)

{

longitude = 99.3759;

latitude = 51.16903;

color = "green";

};

R_Date("Bayankhongor BSA DS1 horse tooth", 2977,30)

{

longitude = 100.80361111111111;

latitude = 46.406816666666664;

color = "green";

};

R_Date("Uvs ZK 1-1 horse tooth", 2963,31)

{

latitude = 49.325500000000005;

longitude = 95.44366666666667;

color = "green";

};

R_Date("Bayankhongor SC DS1 horse tooth", 2953,31)

{

latitude = 46.32743333333333;

longitude = 100.79448333333333;

color = "green";

};

R_Date("Khuvsgul UT DS4 F2 horse bone", 2950,40)

{

longitude = 99.80417;

latitude = 49.93178;

color = "green";

};

R_Date("Khuvsgul UU SC5 horse bone", 2923, 59)

{

longitude = 99.92836;

latitude = 49.65531;

color = "green";

};

R_Date("Khuvsgul TK DS1F1 horse tooth", 2920,40)

{

longitude = 99.3759;

latitude = 51.16903;

color = "green";

};

R_Date("Khuvsgul KG A3F3 horse tooth", 2910,40)

{

longitude = 98.59513;

latitude = 49.70435;

color = "green";

};

R_Date("Khuvsgul Khu. A F18 horse tooth", 2880,40)

{

longitude = 99.89448;

latitude = 48.7048;

color = "green";

};

R_Date("Arkhangai UTAS DS62F17 horse bone", 2880,30)

{

color = "green";

};

R_Date("Khuvsgul UU SH18 horse bone", 2871, 59)

{

longitude = 99.92836;

latitude = 49.65531;

color = "green";

};

R_Date("Khuvsgul ZG A1F3 horse tooth", 2870,40)

{

longitude = 99.84973;

latitude = 49.30937;

color = "green";

};

R_Date("Khuvsgul ZG A3F1 horse tooth", 2860,40)

{

longitude = 99.84973;

latitude = 49.30937;

color = "green";

};

R_Date("Bayankhongor SC DS2 horse tooth", 2846,30)

{

latitude = 46.32743333333333;

longitude = 100.79448333333333;

color = "green";

};

R_Date("Khuvsgul UU KH1 SH11 horse bone", 2835, 57)

{

longitude = 99.92836;

latitude = 49.65531;

color = "green";

};

R_Date("Khuvsgul UT DS5 F2 horse tooth", 2830,40)

{

longitude = 99.80417;

latitude = 49.93178;

color = "green";

};

R_Date("Khuvsgul NA DS1/2F1 horse tooth", 2830,40)

{

longitude = 99.79257;

latitude = 48.81925;

color = "green";

};

R_Date("Khuvsgul UT DS4 F3 horse bone", 2810,40)

{

longitude = 99.80417;

latitude = 49.93178;

color = "green";

};

R_Date("Khuvsgul UT DS5 F1 horse tooth", 2800,40)

{

longitude = 99.80417;

latitude = 49.93178;

color = "green";

};

R_Date("Khuvsgul TN F1 horse tooth", 2800,31)

{

longitude = 99.51171666666667;

latitude = 51.4865;

color = "green";

};

R_Date("Khuvsgul TA DS2 F2 horse tooth", 2790,40)

{

longitude = 99.31233;

latitude = 48.88922;

color = "green";

};

R_Date("Khuvsgul UT DS4 F5 horse bone", 2790,70)

{

longitude = 99.80417;

latitude = 49.93178;

color = "green";

};

R_Date("Arkhangai Khav.A DS40F4 horse bone", 2780,30)

{

color = "green";

};

R_Date("Khuvsgul KG F2 horse tooth", 2750,40)

{

longitude = 98.59513;

latitude = 49.70435;

color = "green";

};

R_Date("Khuvsgul UU SC7 horse bone", 2749, 50)

{

longitude = 99.92836;

latitude = 49.65531;

color = "green";

};

R_Date("Khuvsgul UT DS4 F6 horse bone", 2740,70)

{

longitude = 99.80417;

latitude = 49.93178;

color = "green";

};

R_Date("Khuvsgul ZG A2 DS4 horse tooth", 2710,40)

{

longitude = 99.84973;

latitude = 49.30937;

color = "green";

};

R_Date("Zavkhan UM DS8F1 horse tooth", 2710,30)

{

longitude = 97.4564;

latitude = 48.5163;

color = "green";

};

R_Date("Khuvsgul KDF3 horse tooth", 2680,40)

{

longitude = 100.0607;

latitude = 49.91718;

color = "green";

};

R_Date("Arkhangai JA DS horse tooth", 2670,30)

{

longitude = 101.092917;

latitude = 48.172222;

color = "green";

};

R_Date("Arkhangai BK DS38F1 horse bone", 2660,30)

{

color = "green";

};

R_Date("Khuvsgul BKG F2 DS horse tooth", 2640, 40)

{

longitude = 98.2971;

latitude = 49.73782;

color = "green";

};

R_Date("Zavkhan DD f1 horse tooth", 2620,30)

{

longitude = 97.16444;

latitude = 47.22835;

color = "green";

};

R_Date("Khuvsgul KW DSF1 horse bone", 2610,40)

{

longitude = 99.8991;

latitude = 49.8146;

color = "green";

};

R_Date("Arkhangai BK DS38F95 horse bone", 2580,30)

{

color = "green";

};

R_Date("Khuvsgul UT DS4F1 horse bone", 2530,40)

{

longitude = 99.80417;

latitude = 49.93178;

color = "green";

};

R_Date("Khuvsgul KDF2 horse tooth", 2450,40)

{

longitude = 100.0607;

latitude = 49.91718;

color = "green";

};

R_Date("Khuvsgul Kho. A F1 horse tooth", 2438,33)

{

longitude = 99.12455;

latitude = 50.84653333333333;

color = "green";

};

R_Date("Khuvsgul KDF1 horse tooth", 2410,40)

{

longitude = 100.0607;

latitude = 49.91718;

color = "green";

};

Span("Span of DS dates");

Interval("Duration DS");

Sum("DS");

};

Boundary("DS End")

{

Transition("T");

Start("S");

End("E");

};

};

Sequence()

{

Boundary("Khirigsuur satellite Start")

{

Transition("T");

Start("S");

End("E");

};

Phase()

{

R_Date("Arkhangai Tsatsyn Ereg B10 SAT666 horse tooth", 2820,35)

{

latitude = 47.981369;

longitude = 101.546887;

color = "blue";

};

R_Date("Arkhangai Tsatsyn Ereg B10 KTS01-S2 horse tooth", 2880,25)

{

latitude = 47.981369;

longitude = 101.546887;

color = "blue";

};

R_Date("Arkhangai Tsatsyn Ereg B10 KTS01-S5 horse tooth", 2890,25)

{

latitude = 47.981369;

longitude = 101.546887;

color = "blue";

};

R_Date("Arkhangai Tsatsyn Ereg B10 Structure 111 animal bone", 2855,20)

{

latitude = 47.981369;

longitude = 101.546887;

color = "blue";

};

R_Date("Arkhangai Tsatsyn Ereg B10 Structure 118 animal bone", 2770,20)

{

latitude = 47.981369;

longitude = 101.546887;

color = "blue";

};

R_Combine("Arkhangai Tsatsyn Ereg B10 Structure 5 horse tooth")

{

R_Date("ECHo 1", 2840, 25)

{

latitude = 47.981369;

longitude = 101.546887;

color = "blue";

};

R_Date("ECHo 2", 2870, 25)

{

color = "blue";

};

R_Date("ECHo 3", 2850, 25)

{

color = "blue";

};

};

R_Combine("Arkhangai Tsatsyn Ereg B10 Structure 11 horse tooth")

{

R_Date("ECHo 1", 2805, 25)

{

latitude = 47.981369;

longitude = 101.546887;

color = "blue";

};

R_Date("ECHo 2", 2745, 25)

{

color = "blue";

};

};

R_Date("Dundgobi BGC EX 07.24 animal bone", 3060,40)

{

latitude = 46.1811607;

longitude = 106.0449664;

color = "blue";

};

R_Date("Bayankhongor BSA KS 1 sheep tooth", 2871,31)

{

color = "blue";

latitude = 46.40682;

longitude = 100.8036;

};

R_Date("Uvurkhangai KS 4.11 animal bone", 2830,40)

{

color = "blue";

};

R_Date("Bulgan Tarvagtai 2-1 indet", 2780,20)

{

longitude = 103.2333;

latitude = 49.7684;

color = "blue";

};

R_Date("Khuvsgul M49.2 horse bone", 2730,50)

{

latitude = 49.7810174;

longitude = 99.7996813;

color = "blue";

};

R_Date("Khuvsgul NA M1F1 horse tooth", 2630, 40)

{

longitude = 99.79257;

latitude = 48.81925;

color = "blue";

};

R_Date("Bulgan Tarvagtai 2-2 indet", 2810,25)

{

longitude = 103.2333;

latitude = 49.7684;

color = "blue";

};

R_Date("Khuvsgul M54.22 horse bone", 2843,41)

{

latitude = 49.7810174;

longitude = 99.7996813;

color = "blue";

};

R_Date("Uvs ZK 257-1 horse tooth", 2836,30)

{

latitude = 49.29002777777777;

longitude = 95.50216666666667;

color = "blue";

};

R_Date("Khuvsgul UT M1F2 horse tooth", 2860,40)

{

longitude = 99.8039;

latitude = 49.93083;

color = "blue";

};

R_Date("Uvs ZK 257-4 horse tooth", 2850,29)

{

latitude = 49.29036111111111;

longitude = 95.50241666666666;

color = "blue";

};

R_Date("Uvurkhangai, Khujirt, KS4.32 horse bone", 2880, 25)

{

longitude = 102.4402;

latitude = 46.95934;

color = "blue";

};

R_Date("Uvs ZK 1-3 horse tooth", 2922,30)

{

latitude = 49.325583333333334;

longitude = 95.44227777777778;

color = "blue";

};

R_Date("Bulgan UG KS64-4 horse tooth", 2903,30)

{

longitude = 103.22675;

latitude = 49.59444444444445;

color = "blue";

};

R_Date("Uvurkhangai, Khujirt, KS12.9 horse bone", 2887, 38)

{

longitude = 102.4402;

latitude = 46.95934;

color = "blue";

};

R_Date("Khuvsgul ZG K3F42 horse tooth", 2950,40)

{

longitude = 99.84973;

latitude = 49.30937;

color = "blue";

};

R_Date("Khuvsgul UTM1F3 horse tooth", 2950,60)

{

longitude = 99.8039;

latitude = 49.93083;

color = "blue";

};

R_Date("Khuvsgul ZK FA horse tooth", 2934,31)

{

latitude = 51.42135;

longitude = 99.3646;

color = "blue";

};

R_Date("Dundgobi BGC EX 04.04 horse bone", 3040,40)

{

latitude = 46.1811607;

longitude = 106.0449664;

color = "blue";

};

R_Date("Bayankhongor SC KS1 horse tooth", 2955,31)

{

latitude = 46.32743333333333;

longitude = 100.79448333333333;

color = "blue";

};

R_Date("Bayan-Ulgii OK horse tooth", 2930,40)

{

longitude = 88.6054;

latitude = 48.60656;

color = "blue";

};

R_Date("Arkhangai UB K1-22 horse tooth", 2790,40)

{

longitude = 101.0584;

latitude = 48.09243;

color = "blue";

};

R_Date("Arkhangai UB K1-21 horse tooth", 2780,50)

{

longitude = 101.0578;

latitude = 48.09245;

color = "blue";

};

Span("Span of Khirigsuur satellite dates");

Interval("Duration Khirigsuur satellite");

Sum("Khirigsuur satellite");

};

Boundary("Khirigsuur satellite End")

{

Transition("T");

Start("S");

End("E");

};

};

};

**Burial traditions – quality-control restricted model with trapezoid prior**

Plot()

{

Outlier_Model("RScaled",T(5),U(0,4),"r");

Sequence()

{

Boundary("Afanasievo Start")

{

Transition("T");

Start("S");

End("E");

};

Phase("Afanasievo")

{

R_Date("Bayankhongor aimag, Shatar Chuluu Grave 5 (AT-25) human bone", 4410,31)

{

latitude = 46.30561667;

longitude = 100.7613889;

color = "black";

};

Span("Span of Afanasievo dates");

Interval("Duration Afanasievo");

Sum("Afanasievo ");

};

Boundary("Afanasievo End")

{

Transition("T");

Start("S");

End("E");

};

};

Sequence()

{

Boundary("Chemurchek Start")

{

Transition("T");

Start("S");

End("E");

};

Phase("Chemurchek")

{

R_Date("Bayan-Ulgii aimag, Khundii Gobi Kurgan 1 burial 2 (AT-628) human bone", 4114,29)

{

latitude = 49.355;

longitude = 89.0286111;

color = "white";

};

Span("Span of Chemurchek dates");

Interval("Duration Chemurchek");

Sum("Chemurchek");

};

Boundary("Chemurchek End")

{

Transition("T");

Start("S");

End("E");

};

};

Sequence()

{

Boundary("Khirigsuur satellite Start")

{

Transition("T");

Start("S");

End("E");

};

Phase()

{

R_Date("Arkhangai Tsatsyn Ereg B10 ST101 animal bone", 2845,20)

{

latitude = 47.981369;

longitude = 101.546887;

color = "blue";

};

R_Date("Arkhangai Tsatsyn Ereg B10 ST102 animal bone ", 2825,20)

{

latitude = 47.981369;

longitude = 101.546887;

color = "blue";

};

R_Date("Arkhangai Tsatsyn Ereg B10 ST103 animal bone", 2855,20)

{

latitude = 47.981369;

longitude = 101.546887;

color = "blue";

};

R_Date("Arkhangai Tsatsyn Ereg B10 ST104 animal bone", 2845,20)

{

latitude = 47.981369;

longitude = 101.546887;

color = "blue";

};

R_Date("Arkhangai Tsatsyn Ereg B10 ST107 animal bone", 2805,20)

{

latitude = 47.981369;

longitude = 101.546887;

color = "blue";

};

R_Date("Arkhangai Tsatsyn Ereg B10 ST108 animal bone", 2805,20)

{

latitude = 47.981369;

longitude = 101.546887;

color = "blue";

};

R_Date("Arkhangai Tsatsyn Ereg B10 ST109 animal bone", 2810,20)

{

latitude = 47.981369;

longitude = 101.546887;

color = "blue";

};

R_Date("Arkhangai Tsatsyn Ereg B10 ST110 animal bone", 2790,20)

{

latitude = 47.981369;

longitude = 101.546887;

color = "blue";

};

R_Date("Arkhangai Tsatsyn Ereg B10 ST105 animal bone", 2845,20)

{

latitude = 47.981369;

longitude = 101.546887;

color = "blue";

};

R_Date("Arkhangai Tsatsyn Ereg B10 ST111 animal bone", 2855,20)

{

latitude = 47.981369;

longitude = 101.546887;

color = "blue";

};

R_Date("Arkhangai Tsatsyn Ereg B10 ST112 animal bone", 2845,20)

{

latitude = 47.981369;

longitude = 101.546887;

color = "blue";

};

R_Date("Arkhangai Tsatsyn Ereg B10 ST113 animal bone", 2860,20)

{

latitude = 47.981369;

longitude = 101.546887;

color = "blue";

};

R_Date("Arkhangai Tsatsyn Ereg B10 ST114 animal bone", 2785,20)

{

latitude = 47.981369;

longitude = 101.546887;

color = "blue";

};

R_Date("Arkhangai Tsatsyn Ereg B10 ST115 animal bone", 2825,20)

{

latitude = 47.981369;

longitude = 101.546887;

color = "blue";

};

R_Date("Arkhangai Tsatsyn Ereg B10 ST116 animal bone", 2805,20)

{

latitude = 47.981369;

longitude = 101.546887;

color = "blue";

};

R_Date("Arkhangai Tsatsyn Ereg B10 ST117 animal bone", 2850,20)

{

latitude = 47.981369;

longitude = 101.546887;

color = "blue";

};

R_Date("Arkhangai Tsatsyn Ereg B10 ST118 animal bone", 2770,20)

{

latitude = 47.981369;

longitude = 101.546887;

color = "blue";

};

R_Date("Arkhangai Tsatsyn Ereg B10 C476 animal bone", 2830,20)

{

latitude = 47.981369;

longitude = 101.546887;

color = "blue";

};

R_Date("Arkhangai Tsatsyn Ereg B10 C531 animal bone", 2840,20)

{

latitude = 47.981369;

longitude = 101.546887;

color = "blue";

};

R_Date("Arkhangai Tsatsyn Ereg B10 C1162 animal bone", 2855,20)

{

latitude = 47.981369;

longitude = 101.546887;

color = "blue";

};

R_Date("Arkhangai Tsatsyn Ereg B10 ST1 horse bone", 2810,25)

{

latitude = 47.981369;

longitude = 101.546887;

color = "blue";

};

R_Date("Arkhangai Tsatsyn Ereg B10 ST2 horse tooth", 2845,25)

{

latitude = 47.981369;

longitude = 101.546887;

color = "blue";

};

R_Date("Arkhangai Tsatsyn Ereg B10 ST3 horse bone", 2915,25)

{

latitude = 47.981369;

longitude = 101.546887;

color = "blue";

};

R_Combine("Arkhangai Tsatsyn Ereg B10 ST5 horse tooth")

{

R_Date("ECHo 1", 2840, 25)

{

latitude = 47.981369;

longitude = 101.546887;

color = "blue";

};

R_Date("ECHo 2", 2870, 25)

{

color = "blue";

};

R_Date("ECHo 3", 2850, 25)

{

color = "blue";

};

};

R_Date("Arkhangai Tsatsyn Ereg B10 ST8 horse tooth", 2850,25)

{

latitude = 47.981369;

longitude = 101.546887;

color = "blue";

};

R_Date("Arkhangai Tsatsyn Ereg B10 ST9 horse bone", 2840,25)

{

latitude = 47.981369;

longitude = 101.546887;

color = "blue";

};

R_Date("Arkhangai Tsatsyn Ereg B10 ST10 horse tooth", 2780,25)

{

latitude = 47.981369;

longitude = 101.546887;

color = "blue";

};

R_Combine("Arkhangai Tsatsyn Ereg B10 ST11 horse tooth")

{

R_Date("ECHo 1", 2805, 25)

{

latitude = 47.981369;

longitude = 101.546887;

color = "blue";

};

R_Date("ECHo 2", 2745, 25)

{

color = "blue";

};

};

R_Date("Arkhangai Tsatsyn Ereg B10 ST12 horse bone", 2850,25)

{

latitude = 47.981369;

longitude = 101.546887;

color = "blue";

};

R_Date("Arkhangai Tsatsyn Ereg B10 ST4 horse tooth", 2855,25)

{

latitude = 47.981369;

longitude = 101.546887;

color = "blue";

};

R_Date("Arkhangai Tsatsyn Ereg B10 ST15 horse tooth", 2875,25)

{

latitude = 47.981369;

longitude = 101.546887;

color = "blue";

};

R_Date("Arkhangai Tsatsyn Ereg B10 ST18 horse tooth", 2860,25)

{

latitude = 47.981369;

longitude = 101.546887;

color = "blue";

};

R_Combine("Arkhangai Tsatsyn Ereg B10 SAT354 horse tooth")

{

R_Date("ECHo 1", 2880, 25)

{

latitude = 47.981369;

longitude = 101.546887;

color = "blue";

};

R_Date("ECHo 2", 2845, 30)

{

color = "blue";

};

};

R_Date("Arkhangai Tsatsyn Ereg B10 SAT397 horse tooth", 2900,25)

{

latitude = 47.981369;

longitude = 101.546887;

color = "blue";

};

R_Date("Arkhangai Tsatsyn Ereg B10 SAT415 horse tooth", 2840,25)

{

latitude = 47.981369;

longitude = 101.546887;

color = "blue";

};

R_Date("Arkhangai Tsatsyn Ereg B10 SAT416 horse tooth", 2865,30)

{

latitude = 47.981369;

longitude = 101.546887;

color = "blue";

};

R_Date("Arkhangai Tsatsyn Ereg B10 SAT528 horse tooth", 2815,25)

{

latitude = 47.981369;

longitude = 101.546887;

color = "blue";

};

R_Date("Arkhangai Tsatsyn Ereg B10 SAT666 horse tooth", 2820,35)

{

latitude = 47.981369;

longitude = 101.546887;

color = "blue";

};

R_Date("Arkhangai Tsatsyn Ereg B10 SAT732 horse tooth", 2885,30)

{

latitude = 47.981369;

longitude = 101.546887;

color = "blue";

};

R_Date("Arkhangai Tsatsyn Ereg B10 SAT799 horse tooth", 2845,30)

{

latitude = 47.981369;

longitude = 101.546887;

color = "blue";

};

R_Date("Arkhangai Tsatsyn Ereg B10 SAT810 horse tooth", 2850,25)

{

latitude = 47.981369;

longitude = 101.546887;

color = "blue";

};

R_Combine("Arkhangai Tsatsyn Ereg B10 SAT 811 horse tooth")

{

R_Date("ECHo 1", 2850, 25)

{

latitude = 47.981369;

longitude = 101.546887;

color = "blue";

};

R_Date("ECHo 2", 2805, 25)

{

color = "blue";

};

R_Date("ECHo 3", 2865, 25)

{

color = "blue";

};

};

R_Combine("Arkhangai Tsatsyn Ereg B10 SAT 1023 horse tooth")

{

R_Date("ECHo 1", 2865, 25)

{

latitude = 47.981369;

longitude = 101.546887;

color = "blue";

};

R_Date("ECHo 2", 2840, 25)

{

color = "blue";

};

};

R_Date("Arkhangai Tsatsyn Ereg B10 KTS01-S2 horse tooth", 2880,25)

{

latitude = 47.981369;

longitude = 101.546887;

color = "blue";

};

R_Date("Arkhangai Tsatsyn Ereg B10 KTS01-S5 horse tooth", 2890,25)

{

latitude = 47.981369;

longitude = 101.546887;

color = "blue";

};

R_Date("Arkhangai Tsatsyn Ereg B10 ST16 horse tooth", 2825, 25)

{

latitude = 47.981369;

longitude = 101.546887;

color = "blue";

};

R_Combine("Arkhangai Tsatsyn Ereg B10 ST17 horse tooth")

{

R_Date("ECHo 1", 2905, 25)

{

latitude = 47.981369;

longitude = 101.546887;

color = "blue";

};

R_Date("ECHo 2", 2805, 25)

{

color = "blue";

};

};

Span("Span of Khirigsuur satellite dates");

Interval("Duration Khirigsuur satellite");

Sum("Khirigsuur satellite");

};

Boundary("Khirigsuur satellite End")

{

Transition("T");

Start("S");

End("E");

};

};

Sequence()

{

Boundary("Baitag Start")

{

Transition("T");

Start("S");

End("E");

};

Phase("Baitag")

{

R_Date("Khovd aimag, Uliastai River (lower terrace) I, Kurgan 4(AT-677)",2805, 16)

{

latitude = 45.85636;

longitude = 91.931779;

color = "pink";

};

Span("Span of Baitag dates");

Interval("Duration Baitag ");

Sum("Baitag");

};

Boundary("Baitag End")

{

Transition("T");

Start("S");

End("E");

};

};

Sequence()

{

Boundary("Ulaanzuukh Start")

{

Transition("T");

Start("S");

End("E");

};

Phase("Ulaanzuukh")

{

R_Combine("Ulaanzuukh, Grave 1 (AT-824) human bone")

{

R_Date("Oxford 1", 3110, 31)

{

latitude = 46.28406;

longitude = 111.768265;

color = "red";

};

R_Date("Groningen 2", 3069, 16)

{

color = "red";

};

};

R_Date("Ulaanzuukh, Grave 1 (AT-823) human bone", 3028, 25)

{

latitude = 46.28406;

longitude = 111.768265;

color = "red";

};

R_Date("Ulaanzuukh, Grave 33 (AT-921) human bone", 3075, 27)

{

latitude = 46.28406;

longitude = 111.768265;

color = "red";

};

Span("Span of Ulaanzuukh dates");

Interval("Duration Ulaanzuukh");

Sum("Ulaanzuukh");

};

Boundary("Ulaanzuukh End")

{

Transition("T");

Start("S");

End("E");

};

};

Sequence()

{

Boundary("Slab Burial Start")

{

Transition("T");

Start("S");

End("E");

};

Phase("Slab Burial")

{

R_Date("Dartsagt, Grave 2 (AT-766) human bone", 2436, 26)

{

latitude = 47.91111111;

longitude = 106.7486111;

color = "white";

};

Span("Span of Slab Burial dates");

Interval("Duration Slab Burial");

Sum("Slab Burial");

};

Boundary("Slab Burial End")

{

Transition("T");

Start("S");

End("E");

};

};

Sequence()

{

Boundary("Rando Start")

{

Transition("T");

Start("S");

End("E");

};

Phase("Rando")

{

R_Combine("Khoit Tsenkher Cave, AT-499, Stone mounds grave 11")

{

R_Date("Oxford 1", 3036, 27)

{

latitude = 47.059972;

longitude = 91.842502;

color = "yellow";

};

R_Date("Oxford 2", 2988, 29)

{

color = "yellow";

};

};

Span("Span of Rando dates");

Interval("Duration Rando");

Sum("Rando");

};

Boundary("Rando End")

{

Transition("T");

Start("S");

End("E");

};

};

Sequence()

{

Boundary("DS Start")

{

Transition("T");

Start("S");

End("E");

};

Phase("DS")

{

R_Date("Arkhangai Tsatsyn Ereg DS38 F27 tooth", 2840,25)

{

latitude = 47.981369;

longitude = 101.546887;

color = "green";

};

R_Date("Arkhangai Tsatsyn Ereg DS38 F95 tooth", 2840,25)

{

latitude = 47.981369;

longitude = 101.546887;

color = "green";

};

R_Date("Arkhangai Tsatsyn Ereg DS38 F1 ST1 tooth", 2860,25)

{

latitude = 47.981369;

longitude = 101.546887;

color = "green";

};

Span("Span of DS dates");

Interval("Duration DS");

Sum("DS");

};

Boundary("DS End")

{

Transition("T");

Start("S");

End("E");

};

};

};
