# Supplementary material for: Radiocarbon dating and cultural dynamics across Mongolia’s early pastoral transition
Source: PLoS One. 2019 Nov 6;14(11):e0224241. doi: 10.1371/journal.pone.0224241 (PMC6834239; doi:10.1371/journal.pone.0224241)
Supplement: S1 Table — Specimens highlighted in dark gray fail both of the QC criteria recommended by Zazzo et al (2019), while those highlighted in light gray fail have a collagen yield below 5% but a C/N ratio of less than 3.3. (DOCX) [file pone.0224241.s003.docx]

S1 Table. New radiocarbon dates produced through this study. Specimens highlighted in dark gray meet both of the QC criteria recommended by Zazzo et al (2019), while those highlighted in light gray fail have a collagen yield below 5% but a C/N ratio of less than 3.3.

| **Reference** | **Location** | **^14^C Date Ref** | **^14^C Date (BP)** | **σ** | **Material** | **Monument type** | **Previous dates on this specimen?** | **%Yield** | **C/N ratio** |
| --- | --- | --- | --- | --- | --- | --- | --- | --- | --- |
| AT-25 | Bayankhongor aimag, Erdentsogt sum, Shatar Chuluu, Grave 5 | OxA-36221 | 4410 | 31 | Human bone (rib) | Afanasievo |  | 17.8 | 3.267 |
| AT-26 | Bayankhongor aimag, Erdentsogt sum, Shatar Chuluu, Grave 2 | OxA-36222 | 4415 | 31 | Human tooth | Afanasievo |  | 2 | 3.273 |
| AT-635 | Bayan-Ulgii aimag, Ulaankhus sum, Khuurai Gobi, Kurgan 2 | OxA-43603 | FAIL - low collagen |  | Human tooth | Afanasievo | X |  |  |
| AT-635 | Bayan-Ulgii aimag, Ulaankhus sum, Khuurai Gobi, Kurgan 2 | GrM-12938 | 4034 | 16 | Human tooth | Afanasievo | X | 4.9 | 3.2 |
| AT-628 | Bayan-Ulgii aimag, Ulaankhus sum, Khundii Gobi, Kurgan 1, Burial 2 | OxA-36230 | 4114 | 29 | Human bone (rib) | Chemurchek/Afanasievo | X | 11.7 | 3.253 |
| AT-590B | Khovd aimag, Bulgan sum, Yagshiin Khuduu, Grave 1 | P-43597 | FAIL - low collagen |  | Human tooth | Chemurchek | X |  |  |
| AT-590B | Khovd aimag, Bulgan sum, Yagshiin Khuduu, Grave 1 | GrM-12984 | 3983 | 17 | Human tooth | Chemurchek | X | 3.7 | 3.2 |
| AT-614 | Khovd aimag, Munkkhairkhan sum, Ulaan Goviin Uzuur, Grave 2 | OxA-X-2737-53 | 3421 | 32 | Human tooth | Munkhairkhan | X | 1.6 | 3.394 |
| AT-960 | Khovd aimag, Munkhkhairkhan sum, Shar Gobi 3, Kurgan 1 | OxA-36455 | 3107 | 31 | Human tooth | Munkhairkhan |  | 1.1 | 3.205 |
| AT-861 | Zavkhan aimag, Bayantes sum, Khukh Khosbuunii Boom | P-43601 | FAIL-low collagen |  | Human tooth | Munkhairkhan |  | - | - |
| AT-769 | Sukhbaatar aimag, Tuvshinshiree sum, Ulaanzuukh, Grave 42 | OxA-36459 | 3215 | 40 | Human tooth | Ulaanzuukh |  | 0.8 | 3.319 |
| AT-824 | Sukhbaatar aimag, Tuvshinshiree sum, Ulaanzuukh, Grave 1 | OxA-36460 | 3110 | 31 | Human bone (rib) | Ulaanzuukh |  | 1 | 3.237 |
| AT-824 | Sukhbaatar aimag, Tuvshinshiree sum, Ulaanzuukh, Grave 1 | GrM-12986 | 3069 | 16 | Human bone (rib) | Ulaanzuukh |  | 11.4 | 3.2 |
| AT-921 | Sukhbaatar aimag, Tuvshinshiree sum, Ulaanzuukh, Grave 33 | OxA-36232 | 3075 | 27 | Human bone (rib) | Ulaanzuukh |  | 16.4 | 3.198 |
| AT-823 | Sukhbaatar aimag, Tuvshinshiree sum, Ulaanzuukh, Grave 2 | OxA-36231 | 3028 | 25 | Humane bone (rib) | Ulaanzuukh | X | 13.8 | 3.217 |
| AT-923 | Sukhbaatar aimag, Tuvshinshiree sum, Ulaanzuukh | P-43607 | FAIL-low collagen |  | Human Bone (rib) | Ulaanzuukh |  | - | - |
| AT-920 | Sukhbaatar aimag, Tuvshinshiree sum, Ulaanzuukh | P-43612 | FAIL-low collagen |  | Human bone (femur) | Ulaanzuukh |  | - | - |
| AT-499 | Khovd aimag, Mankhan sum, Khoit Tsenkher, Stone mounds grave 11 | OxA-36229 | 3036 | 27 | Human bone (petrous) | Indeterminate Mound |  | 12.8 | 3.266 |
| AT-499 | Khovd aimag, Mankhan sum, Khoit Tsenkher, Stone mounds grave 11 | OxA-36228 | 2988 | 29 | Human Bone (petrous) | Indeterminate Mound |  | 11.7 | 3.294 |
| AT-398 | Khovd aimag, Mankhan sum, Khoit Tsenkher Cave, Grave 2 | OxA-36227 | 2828 | 28 | Human tooth | Indeterminate Mound |  | 2.4 | 3.283 |
| AT-676 | Khovd aimag, Uyench sum, Uliastai Zastav,Kurgan 1, Burial 4 | OxA-X-2737-54 | 2967 | 31 | Human tooth | Sagsai |  | 1.6 | 3.481 |
| AT-617 | Khuvsgul aimag, Alag-Erdene sum, Erkhel/Ulaan Tolgoi | OxA-36226 | 2921 | 28 | Human tooth | Khirigsuur |  | 2.2 | 3.213 |
| AT-905 | Khovd aimag, Mankhan sum, Berkh Mountain, Khirigsuur 3 | OxA-36225 | 2989 | 28 | Human tooth | Khirigsuur |  | 4.2 | 3.447 |
| AT-674 | Khovd aimag, Uyench sum, Uliastai Zastav, Kurgan 2 | OxA-36224 | 2842 | 28 | Human tooth | Baitag |  | 2.7 | 3.293 |
| AT-677 | Khovd aimag, Uyench sum, Uliastai River (lower terrace) I, Kurgan 4 | GrM-12982 | 2805 | 16 | Human tooth | Baitag |  | 8.8 | 3.2 |
| AT-233 | Uvurkhangai aimag, Khujirt sum, Shunkhlai Mountain, Grave 9 | OxA-36456 | 2826 | 30 | Human tooth | Slab Burial |  | 1 | 3.225 |
| AT-707 | Khentii aimag, Binder sum, Bor Bulag, Grave 2 | OxA-X-2737-55 | 2759 | 31 | Human tooth | Slab Burial |  | 1.2 | 3.498 |
| AT-766 | Ulaanbaatar, Songinokhairkhan district, Dartsagt, Grave 2 | OxA-36233 | 2436 | 26 | Human bone | Slab Burial |  | 12.9 | 3.206 |
