# Supplementary material for: Radiocarbon dating and cultural dynamics across Mongolia’s early pastoral transition
Source: PLoS One. 2019 Nov 6;14(11):e0224241. doi: 10.1371/journal.pone.0224241 (PMC6834239; doi:10.1371/journal.pone.0224241)
Supplement: S2 Table — (DOCX) [file pone.0224241.s004.docx]

S2 Table A. Model output for start boundaries, end boundaries, and average dates (“sum”) for each culture unit analyzed in this study, sorted according to median values.

| **Name** | **1 sigma cal. range (BCE)** | | **2 sigma cal. range (BCE)** | | **median (BCE)** | **Agreement** |
| --- | --- | --- | --- | --- | --- | --- |
| Afanasievo Start | -3341 | -2954 | -4414 | -2909 | -3169 | 95.1 |
| Afanasievo Sum |  |  |  |  | -2987 | 98.8 |
| Chemurchek Start | -2923 | -2576 | -3337 | -2436 | -2762 | 98.2 |
| Afanasievo End | -3023 | -2525 | -3271 | -1392 | -2726 | 92.9 |
| Chemurchek/Afanasievo Sum |  |  |  |  | -2529 | 36.9 |
| Chemurchek Sum |  |  |  |  | -2380 | 99.9 |
| Chemurchek/Afanasievo End | -2600 | -1631 | -2601 | 128 | -1988 | 13.3 |
| Chemurchek End | -2136 | -1755 | -2283 | -1380 | -1917 | 97.8 |
| Munkkhairkhan Start | -1959 | -1724 | -2255 | -1651 | -1862 | 99.3 |
| Shape Burial Start | -1989 | -1453 | -2747 | -1266 | -1748 | 99.6 |
| Munkkhairkhan Sum |  |  |  |  | -1657 | 100 |
| Indeterminate Mound Start | -1561 | -1365 | -1805 | -1286 | -1474 | 99.6 |
| Ulaanzuukh Start | -1501 | -1434 | -1550 | -1398 | -1469 | 99.9 |
| D Shape Start | -1732 | -1031 | -3474 | -993 | -1413 | 98.8 |
| Khirigsuur mound Start | -1430 | -1265 | -1581 | -1173 | -1352 | 99.7 |
| Munkkhairkhan End | -1471 | -1244 | -1571 | -982 | -1344 | 99.5 |
| Sagsai Start | -1377 | -1285 | -1435 | -1247 | -1332 | 94.6 |
| Ulaanzuukh Sum |  |  |  |  | -1329 | 100 |
| Indeterminate Mound Sum |  |  |  |  | -1228 | 100 |
| Sagsai Sum |  |  |  |  | -1212 | 94.5 |
| Khirigsuur satellite Start | -1223 | -1136 | -1282 | -1102 | -1183 | 99.8 |
| Ulaanzuukh End | -1221 | -1124 | -1258 | -1058 | -1169 | 99.8 |
| DS Start | -1206 | -1123 | -1248 | -1081 | -1164 | 99.9 |
| D Shape Sum |  |  |  |  | -1120 | 99.9 |
| Shape Burial Sum |  |  |  |  | -1097 | 100 |
| Khirigsuur mound Sum |  |  |  |  | -1074 | 100 |
| Slab Burial Start | -1170 | -943 | -1313 | -869 | -1069 | 99.7 |
| Sagsai End | -1126 | -1007 | -1184 | -947 | -1068 | 95.3 |
| Baitag Start | -1145 | -940 | -1722 | -917 | -1055 | 98.8 |
| Khirigsuur satellite Sum |  |  |  |  | -1027 | 100 |
| Baitag Sum |  |  |  |  | -972 | 99.8 |
| Slab Burial animal Start | -1077 | -830 | -1363 | -687 | -958 | 99.6 |
| DS Sum |  |  |  |  | -957 | 100 |
| Baitag End | -991 | -844 | -1027 | -313 | -907 | 99 |
| Khirigsuur mound End | -970 | -831 | -1041 | -666 | -895 | 99.8 |
| Khirigsuur satellite End | -917 | -853 | -950 | -806 | -884 | 99.8 |
| D Shape End | -1203 | -549 | -1247 | 1108 | -862 | 98.9 |
| DS End | -773 | -688 | -835 | -651 | -733 | 99.7 |
| Indeterminate Mound End | -909 | -584 | -1013 | -321 | -713 | 99.5 |
| Slab Burial Sum |  |  |  |  | -657 | 100 |
| Shape Burial End | -886 | -419 | -1048 | 350 | -645 | 99.6 |
| Slab Burial animal Sum |  |  |  |  | -514 | 100 |
| Slab Burial End | -396 | -286 | -459 | -191 | -341 | 99.8 |
| Slab Burial animal End | -176 | 196 | -348 | 454 | 32 | 99.5 |
