# Supplementary material for: Radiocarbon dating and cultural dynamics across Mongolia’s early pastoral transition
Source: PLoS One. 2019 Nov 6;14(11):e0224241. doi: 10.1371/journal.pone.0224241 (PMC6834239; doi:10.1371/journal.pone.0224241)
Supplement: S3 Table — (DOCX) [file pone.0224241.s005.docx]

S3 Table. Model output for start boundaries, end boundaries, and average dates (“sum”) for each burial position unit analyzed in this study, sorted according to median values.

| **Name** | **1 sigma cal. range (BCE)** | | **2 sigma cal. range (BCE)** | | **median (BCE)** | **Agreement** |
| --- | --- | --- | --- | --- | --- | --- |
| Flexed Start | -3235 | -2865 | -3444 | -2554 | -3050 | 99.1 |
| Flexed Sum |  |  |  |  | -2205 |  |
| Prone Start | -1567 | -1482 | -1625 | -1446 | -1528 | 99.9 |
| Supine Start | -1418 | -1343 | -1459 | -1303 | -1379 | 99.8 |
| Supine with knees bent Start | -1454 | -1170 | -1844 | -1046 | -1325 | 99.5 |
| Prone Sum |  |  |  |  | -1319 |  |
| Flexed End | -1445 | -1128 | -1736 | -929 | -1294 | 99.3 |
| Prone End | -1111 | -996 | -1157 | -907 | -1048 | 99.8 |
| Supine Sum |  |  |  |  | -1015 |  |
| Supine with knees bent Sum |  |  |  |  | -980 |  |
| Supine with knees bent End | -792 | -446 | -916 | -52 | -606 | 99.5 |
| Supine End | -721 | -326 | -766 | -301 | -516 | 99.5 |
